# Evaluation of the mechanisms of intron loss and gain in the social amoebae *Dictyostelium*

**DOI:** 10.1186/s12862-015-0567-y

**Published:** 2015-12-18

**Authors:** Ming-Yue Ma, Xun-Ru Che, Andrea Porceddu, Deng-Ke Niu

**Affiliations:** MOE Key Laboratory for Biodiversity Science and Ecological Engineering, College of Life Sciences, Beijing Normal University, Beijing, 100875 China; Beijing Key Laboratory of Gene Resource and Molecular Development, College of Life Sciences, Beijing Normal University, Beijing, 100875 China; The High School Affiliated to Renmin University of China, Beijing, 100080 China; Department of Agricultural Sciences, University of Sassari, Viale Italia, 39, 07100 Sassari, Italy

**Keywords:** *Dictyostelium discoideum*, *Dictyostelium purpureum*, Intron gain, Reverse transcriptase, GC content, Imprecise intron losses

## Abstract

**Background:**

Spliceosomal introns are a common feature of eukaryotic genomes. To approach a comprehensive understanding of intron evolution on Earth, studies should look beyond repeatedly studied groups such as animals, plants, and fungi. The slime mold *Dictyostelium* belongs to a supergroup of eukaryotes not covered in previous studies.

**Results:**

We found 441 precise intron losses in *Dictyostelium discoideum* and 202 precise intron losses in *Dictyostelium purpureum*. Consistent with these observations, *Dictyostelium discoideum* was found to have significantly more copies of reverse transcriptase genes than *Dictyostelium purpureum*. We also found that the lost introns are significantly further from the 5′ end of genes than the conserved introns. Adjacent introns were prone to be lost simultaneously in *Dictyostelium discoideum*. In both *Dictyostelium* species, the exonic sequences flanking lost introns were found to have a significantly higher GC content than those flanking conserved introns. Together, these observations support a reverse-transcription model of intron loss in which intron losses were caused by gene conversion between genomic DNA and cDNA reverse transcribed from mature mRNA. We also identified two imprecise intron losses in *Dictyostelium discoideum* that may have resulted from genomic deletions. Ninety-eight putative intron gains were also observed. Consistent with previous studies of other lineages, the source sequences were found in only a small number of cases, with only two instances of intron gain identified in *Dictyostelium discoideum*.

**Conclusions:**

Although they diverged very early from animals and fungi, *Dictyostelium* species have similar mechanisms of intron loss.

**Electronic supplementary material:**

The online version of this article (doi:10.1186/s12862-015-0567-y) contains supplementary material, which is available to authorized users.

## Background

With the exception of the relics of certain endosymbiotic nuclei [1], all eukaryotic genomes contain spliceosomal introns. Evidence also suggests that eukaryotic genes transferred from organelles or prokaryotes have generally experienced a high rate of intron insertion subsequent to the transfer [2–7]. The existence of spliceosomal introns is a common feature of eukaryotic nuclear genomes. However, previous studies indicated that the dynamic changes in introns vary greatly among eukaryotic lineages [8–14]. Thus, a model that successfully explains the mechanisms of intron loss or gain in some eukaryotic lineages may be inadequate for other lineages [15, 16]. Three models have been proposed for the mechanism of intron loss [17–20]. The first is the reverse transcription model, also termed mRNA-mediated intron loss, in which introns are deleted from the genome by recombination between the genomic DNA and cDNA reverse transcribed from spliced mRNA. In this model, the introns are precisely deleted and adjacent introns tend to be lost simultaneously. As the binding of reverse transcriptase and RNA template is unstable, the reverse transcription process frequently aborts, thus producing incomplete cDNA molecules. Recombination of these cDNAs with genomic DNA would cause a preferential loss of introns from the 3′ end of genes. A long intron is expected to disturb the *in vivo* alignment of homologous regions between the cDNA and the genomic DNA and therefore be lost at a lower frequency than a short intron. The second model of intron loss is simple genomic deletion. In this model, each individual intron is lost independently and without bias with respect to its position within a gene. In this model, introns might occasionally be lost precisely but are typically accompanied by insertions and/or deletions in the flanking exonic sequences. The final model is one in which introns are lost during non-homologous end joining (NHEJ) repair of DNA double-strand breaks. As the repair process generally requires microhomology between the break sites, this model predicts that there should be short direct repeats at the two ends of the lost intron. Besides this, the same predictions are shared between the NHEJ repair model and the genomic deletion model. The first model has been widely supported by studies that are carried on animals, fungi, and plants [12, 13, 16, 21–25]. However, the pattern of intron losses observed in *Arabidopsis thaliana* was different from that predicted by the first model but consistent with the third model in which introns are lost during DNA double-strand break repair [19, 26]. Short direct repeats at the splice sites of lost introns have been detected in plants and invertebrates [25–27], supporting the third model. However, another prediction of the third model, imprecise intron loss, has not been observed to have a high frequency in most eukaryotic lineages [28].

For a comprehensive understanding of intron evolution on Earth, studies should cover all major eukaryotic lineages. However, a considerable bias exists toward a limited number of model organisms in animals, plants, and fungi [9, 12, 16, 21, 23, 24, 26, 27, 29–36], which belong to two (Opisthokonta and Archaeplastida) of the five supergroups of eukaryotes according to the recent consensus phylogenetic tree of eukaryotes [37, 38]. Limited by the biased distribution of sequenced genomes [37], very few studies have been carried out in the most diverse kingdom, Protozoa [11, 39–45]. Even in these few studies, the research materials were heavily biased toward *Plasmodium*, a model lineage of the supergroup SAR (stramenopiles, alveolates, and Rhizaria) [37, 38]. The slime mold *Dictyostelium* belongs to another supergroup of eukaryotes, Amoebozoa [37, 38]. *Dictyostelium* can form differentiated multicellular structures by aggregating thousands of solitary amoebae in response to starvation [46]. The most prominent member, *Dictyostelium discoideum*, has been used as a model organism to study multicellularity, cell differentiation, signal transduction, cell migration, and development for many years [47]. At the genomic level, *Dictyostelium* has two characteristics which make them helpful for the further study on intron evolution. The first one is the enrichment of simple sequence repeats (SSRs) in the *Dictyostelium* genomes [48, 49]. According to the NHEJ repair model of intron loss [19], these SSRs, if exist at the splice sites, could mediated intron losses. The second special characteristic of the *Dictyostelium* species is the 16 documented new genes gained from bacteria by horizontal gene transfer (HGT) after their divergence from plants and animals, but prior to the divergence among themselves [48, 49]. We hope that these new genes might give some implications on the mechanism of intron gain. In the present study, we surveyed the intron losses and gains in both *Dictyostelium discoideum* and *Dictyostelium purpureum* and explored the mechanisms underlying these variations.

## Results and discussion

**Fig. 1:**
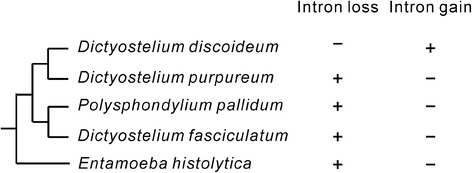
Distinguishing intron loss and gain in *Dictyostelium discoideum* and *Dictyostelium purpureum*. The phylogenetic tree was adapted from DictyBase [70] and is scaled according to phylogenetic distances. Dollo parsimony was used to distinguish intron loss and gain. Examples of intron loss and gain in *Dictyostelium discoideum* are shown using “+” and “−” represent the presence and the absence of an intron in a given position, respectively

By comparing the orthologous genes among *Dictyostelium discoideum*, *Dictyostelium purpureum*, *Polysphondylium pallidum*, *Dictyostelium fasciculatum*, and *Entamoeba histolytica* (Fig. 1), we found 441 precise intron losses, two imprecise intron losses, and 40 putative intron gains in *Dictyostelium discoideum*, and 202 precise intron losses and 58 putative intron gains in *Dictyostelium purpureum* (Additional file 1: Table S1). We use the term “putative” to describe the observed intron gains because false-positive gains are very likely when limited numbers of outgroups are used [31]. We performed Gene Ontology (GO) analysis to examine whether the intron losses and gains are enriched in some special groups of genes or not. Intron loss genes are significantly enriched in 98 GO categories and putative intron gain genes are significantly enriched in 97 GO categories (*P* < 0.01, Additional file 2: Table S2). They share 46 common GO categories. From these GO enrichments, we could not see any implications on the mechanisms of intron loss and gain.

### Molecular mechanisms of intron losses in *Dictyostelium*

**Fig. 2:**
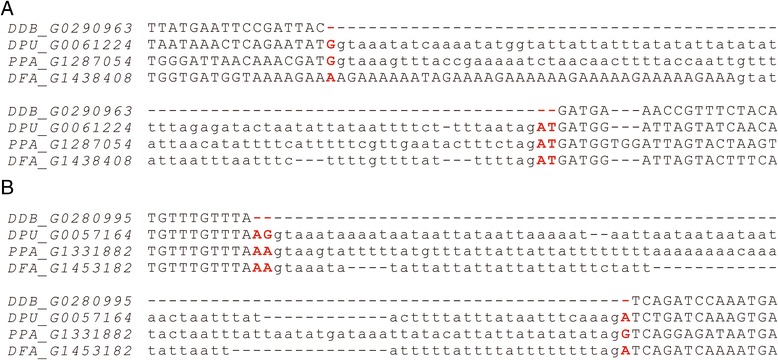
Imprecise intron losses in *Dictyostelium discoideum*. **a** Gene *DDB_G0290963*. **b** Gene *DDB_G0280995*. Abbreviations: *DDB*, *Dictyostelium discoideum*; *DPU*, *Dictyostelium purpureum*; *PPA*, *P. pallidum*; *DFA*, *Dictyostelium fasciculatum*

In the two *Dictyostelium* species, the frequency of imprecise intron loss is also very low: only two cases of imprecise intron loss were observed (compared with 643 cases of precise intron loss). Along with each of the two intron losses, three flanking nucleotides of coding sequences were deleted (Fig. 2). No short direct repeats were observed at the two boundaries of the lost introns. These two observations support the genomic deletion model. It is also possible that deletion of the 3-bp coding sequence occurred independently of precise intron loss events. All other cases of intron losses observed in *Dictyostelium discoideum* and *Dictyostelium purpureum* were not accompanied by an insertion or deletion in flanking exonic sequences. In some cases, we observed short direct repeats at the boundaries of the lost introns. The frequency of lost introns that have short direct repeats at their boundaries depends on the size of the queried repeats. Regardless of their size, their frequency does not differ significantly from the frequency of short direct repeats at the boundaries of conserved introns (χ^2^ tests, *P* > 0.1). Although SSRs are abundant in *Dictyostelium* genomes [48, 49], they unlikely facilitate intron losses. Our results do not support the NHEJ repair model of intron loss [19]. As the detection of imprecise intron losses generally depends on the quality of alignments, only intron losses positioned at well-aligned regions are readily observed. These two mechanisms may be underestimated when the genomes being compared have been divergent for a long time.

**Fig. 3:**
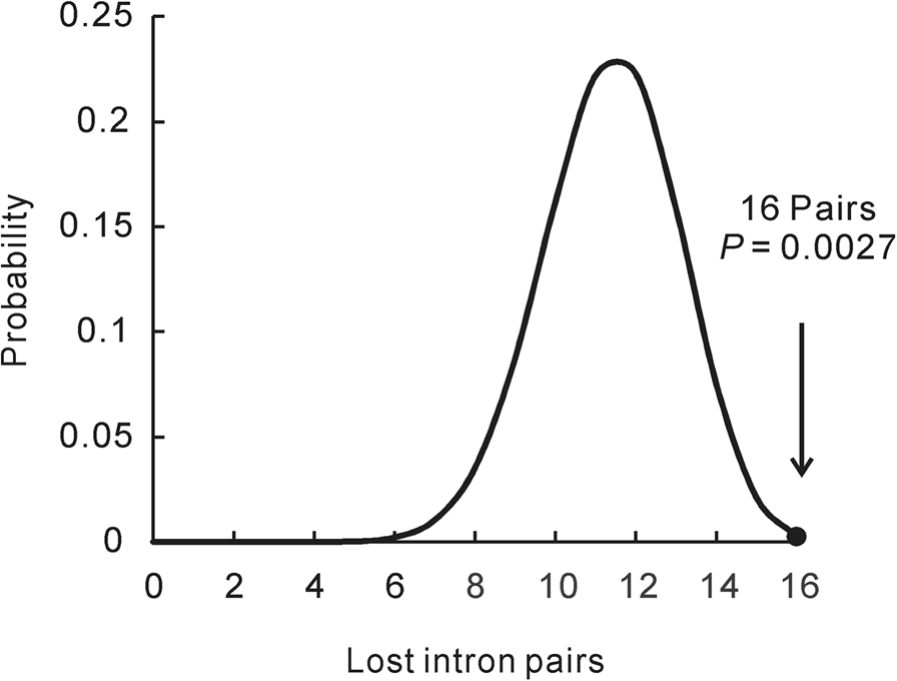
The probability distributions of the loss of adjacent intron pairs in *Dictyostelium discoideum*. The X-axis represents the pairs of adjacent intron losses, and the Y-axis represents their probability of appearance when each intron is lost independently. The observed pattern, 16 pairs of adjacent intron loss, has a very low probability of occurrence via the independent loss of each intron

We next investigated whether the precise intron losses in *Dictyostelium* support the reverse-transcription model. Among the 441 precise intron losses in *Dictyostelium discoideum* and the 202 losses in *Dictyostelium purpureum*, we identified 31 and three pairs of adjacent intron losses, respectively. Here, three adjacent introns were considered as two pairs. The losses of adjacent introns might be either due to the simultaneous loss of adjacent introns or independent losses of different introns happen to be neighboring in position. For this reason, we performed *in silico* resampling analysis by randomly drawing 441 introns from the pool of both the 441 lost introns and the 15,510 extant introns of *Dictyostelium discoideum*. This resampling has been repeated for a total of 10,000 times and an occurrence of 31 or more pairs of adjacent introns was never observed (*P* = 0). In *Dictyostelium purpureum*, 202 introns were re-sampled for 10,000 times and three or more pairs of adjacent introns were obtained in 1,038 times (*P* = 0.1). Similar results were observed in re-sampling analyses that replaced the extant introns by conserved introns. Furthermore, we calculated the probability distribution of the losses of adjacent introns with the assumption that each intron was lost independently [21]. A probability of 0.0027 was obtained for *Dictyostelium discoideum*, which indicates that adjacent introns tend to be lost simultaneously (Fig. 3). Because of the limited number of adjacent intron losses in *Dictyostelium purpureum*, the probability was not calculated. Simultaneous losses of adjacent introns, rather than independent losses of different introns, account for the frequency of adjacent intron losses we observed in *Dictyostelium discoideum*.

**Fig. 4:**
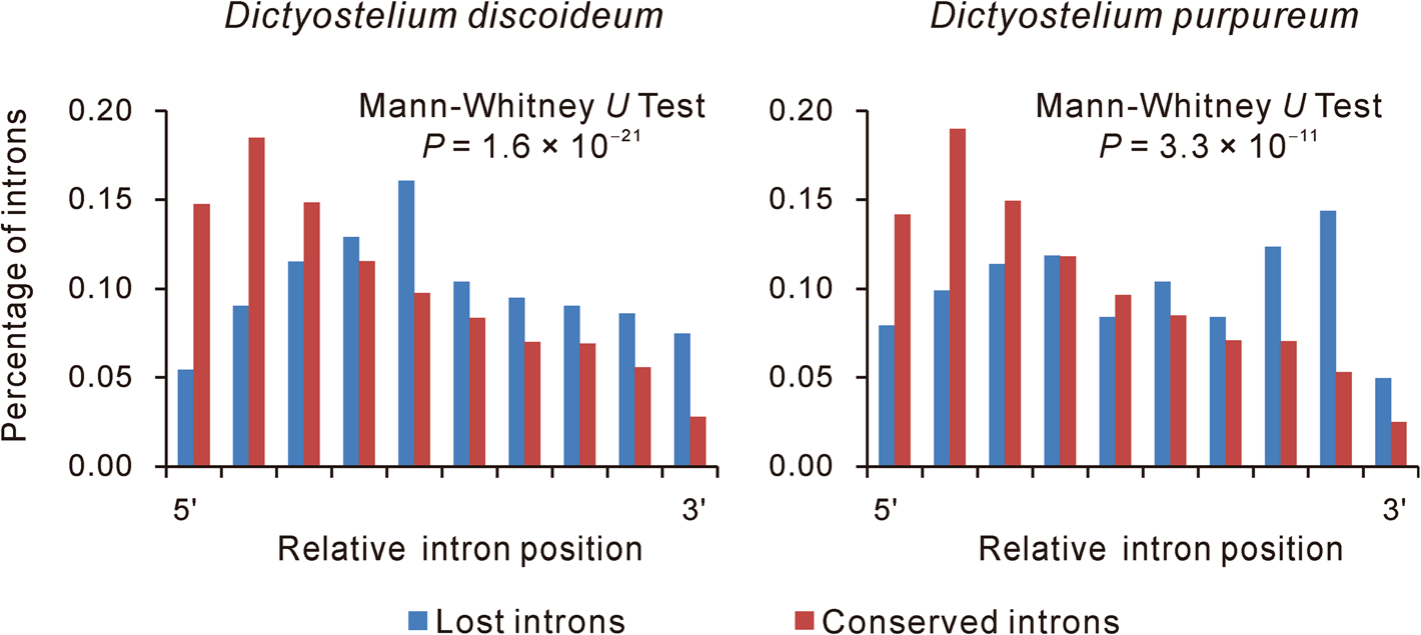
Distribution of lost introns compared with conserved introns. The X-axis represents the relative position of the introns, and the Y-axis represents their percentage of introns. The relative position of each intron, either conserved or lost, within its host gene was calculated as the sequence length of the mRNA upstream of the intron divided by the full length of the mRNA. As the data are not normally distributed, we used nonparametric analyses in our comparisons of the data

Previous surveys of the *Dictyostelium discoideum* genome showed that its extant introns are significantly biased to the 5′ end of genes [50, 51]. We confirmed this observation in *Dictyostelium discoideum* and observed a similar pattern in *Dictyostelium purpureum* (Wilcoxon signed rank test, *P* = 0 for both species). As the extant introns are biased to the 5′ end of genes, most of the 3′-most introns will be in the middle or at 5′ side of genes. The absolute positions of lost introns will not be at the 3′ end of genes. Therefore, we compared the positions of the lost introns and the conserved introns. As shown in Fig. 4, the lost introns are significantly further from the 5′ end of genes than the conserved introns.

We also found that the lost introns are significantly shorter than the conserved introns (Mann–Whitney *U* test, *P* = 0.004 and 0.033, respectively, for *Dictyostelium discoideum* and *Dictyostelium purpureum*). However, their differences are very small (median size: 76 bp vs. 77 bp in *Dictyostelium discoideum* and 100 bp vs. 105 bp in *Dictyostelium purpureum*; mean size: 81 bp vs. 94 bp in *Dictyostelium discoideum* and 113 bp vs. 125 bp in *Dictyostelium purpureum*). Compared with vertebrates, the introns of *Dictyostelium discoideum* and *Dictyostelium purpureum* are very short. Therefore, we are cautious in interpreting these size differences as evidence of the reverse-transcription model.

### Different intron loss rates associated with the abundance of retrotransposons

We observed a large difference in the rate of intron loss between these two species; whereas *Dictyostelium discoideum* was found to have lost 443 introns, *Dictyostelium purpureum* lost only 202 introns. χ^2^ tests showed that this difference is significant, regardless of whether the extant introns were represented by all the annotated introns (number of annotated introns: 15,510 in *Dictyostelium discoideum* and 18,412 in *Dictyostelium purpureum*, *P* = 10^−30^) or only the conserved introns (*P* = 3 × 10^−20^). The genomes of these two species are similar in size, with sizes of 34 and 33 Mb, respectively [49]. Therefore, the difference in intron loss rate is not likely driven by different forces acting on genome size. Two possible explanations based on the reverse-transcription model were tested.

The first possibility is that the introns of *Dictyostelium discoideum* are generally shorter; thus, the genomic DNAs are more suitable substrates of the recombination process than those of *Dictyostelium purpureum*. Whereas *Dictyostelium discoideum* has a mean intron length shorter than that of *Dictyostelium purpureum* (132.55 vs. 162.26 bp, respectively), globally, its introns are significantly longer than those of *Dictyostelium purpureum* (median intron sizes: 104 bp vs. 78 bp, respectively; Additional file 3: Figure S1). *Dictyostelium purpureum* has a longer mean intron length because of its small proportion of extraordinarily large introns.

The second possibility is that *Dictyostelium discoideum* has a higher reverse transcriptase activity and, as a consequence, produces more substrates for the recombination process than *Dictyostelium purpureum*. Following Coulombe-Huntington and Majewski [12], we estimated the activities of reverse transcriptase in these two species based on the abundance of reverse transcriptase genes in the two *Dictyostelium* genomes. From the NCBI Protein database [52], we retrieved sequences of 147 reverse transcriptases for *Dictyostelium discoideum* and ten reverse transcriptases for *Dictyostelium purpureum*. The sequences of the retrieved reverse transcriptases were used as queries to search against all annotated proteins of the two species using BLASTP with an E value threshold of 10^−10^. The copy number of reverse transcriptase genes in the genome of *Dictyostelium discoideum* is approximately 70 times that of *Dictyostelium purpureum*, indicating that reverse transcription is more frequent in *Dictyostelium discoideum* than in *Dictyostelium purpureum* (Table 1). It should be noted that the copies of reverse transcriptase genes include both active reverse transcriptase genes and evolutionary relics of reverse transcriptase genes that have exapted to serve other functions. We believe that including the relics more accurately reflects the activity of reverse transcriptases in the evolutionary history when the introns were lost. As the genome of *Dictyostelium discoideum* has been studied more extensively than that of *Dictyostelium purpureum*, more genes might have been annotated in *Dictyostelium discoideum*. However, the difference of the abundance of the reverse transcriptases seems to be too striking to be accounted for by the annotation bias. In addition, we found that *Dictyostelium discoideum* has a greater number of retrotransposons than *Dictyostelium purpureum* (Table 1). The detection of retrotransposons did not depend on the quality of the genome annotation. The abundance of retrotransposons also indicates a higher reverse transcriptase activity in *Dictyostelium discoideum* than *Dictyostelium purpureum*.

**Table 1:** Copy numbers of reverse transcriptases and retrotransposons

	Reverse transcriptases	Retrotransposons		
		LINEs	SINEs	LTR elements
*Dictyostelium discoideum*	3,402	252	88	3
*Dictyostelium purpureum*	48	93	57	0

LINEs, long interspersed nuclear elements; SINEs, short interspersed nuclear elements; LTR, long terminal repeat. All retrotransposons were detected using RepeatMasker (version open-4.0.0, default mode, and RepBase update 20140131, RM database version 20140131)

It is also possible that intron losses are not strictly neutral [32, 53] and that a difference in the efficiency of natural selection contributed to the observed different rates of intron loss. For example, if intron losses were primarily beneficial, they would be more likely to be fixed in species with a large effective population size. In contrast, if they were slightly deleterious and were fixed by genetic drift, the species with a small effective population size should have lost more introns. The long length of time separating the divergence of the two species, 400 million years, suggests that synonymous substitutions may have become saturated and are impossible to be estimated accurately [49]. Therefore, *d*_N_/*d*_S_, the common method used to estimate the efficiency of natural selection and genetic drift [54, 55], is not applicable in this study. Some researchers believe that introns and other repetitive sequences are slightly deleterious; their abundance, therefore, should be negatively correlated with effective population size [56]. According to this hypothesis, the abundance of introns and the abundance of repetitive sequences should be positively correlated. The genome of *Dictyostelium discoideum* was found to have fewer introns, a shorter total intron length, and more intron losses than that of *Dictyostelium purpureum* (Additional file 3: Table S3). Unexpectedly, however, *Dictyostelium discoideum* has more repetitive sequences than *Dictyostelium purpureum*, even if retrotransposons are withheld from the comparison (Additional file 3: Table S3). Although this evidence is not strong, it indicates that the intron losses in *Dictyostelium* were unlikely to have been driven by the putative selective forces experienced by repetitive sequences. Future polymorphism data of *Dictyostelium* will be essential to infer the contribution of natural selection to the frequency of intron loss.

### High GC contents around lost introns: evidence for biased gene conversion

The above evidence, in addition to data from numerous previous studies, supports the model that an intron can be deleted during a recombination event occurring between genomic DNA and intronless cDNA [12, 16, 21, 23, 24]. However, the details of such a recombination process have seldom been explored [57]; therefore, we cannot ascertain whether this was a gene conversion process or a reciprocal crossover recombination process, although the former has often been used in the description of the reverse-transcription model [21, 29, 58–60].

**Fig. 5:**
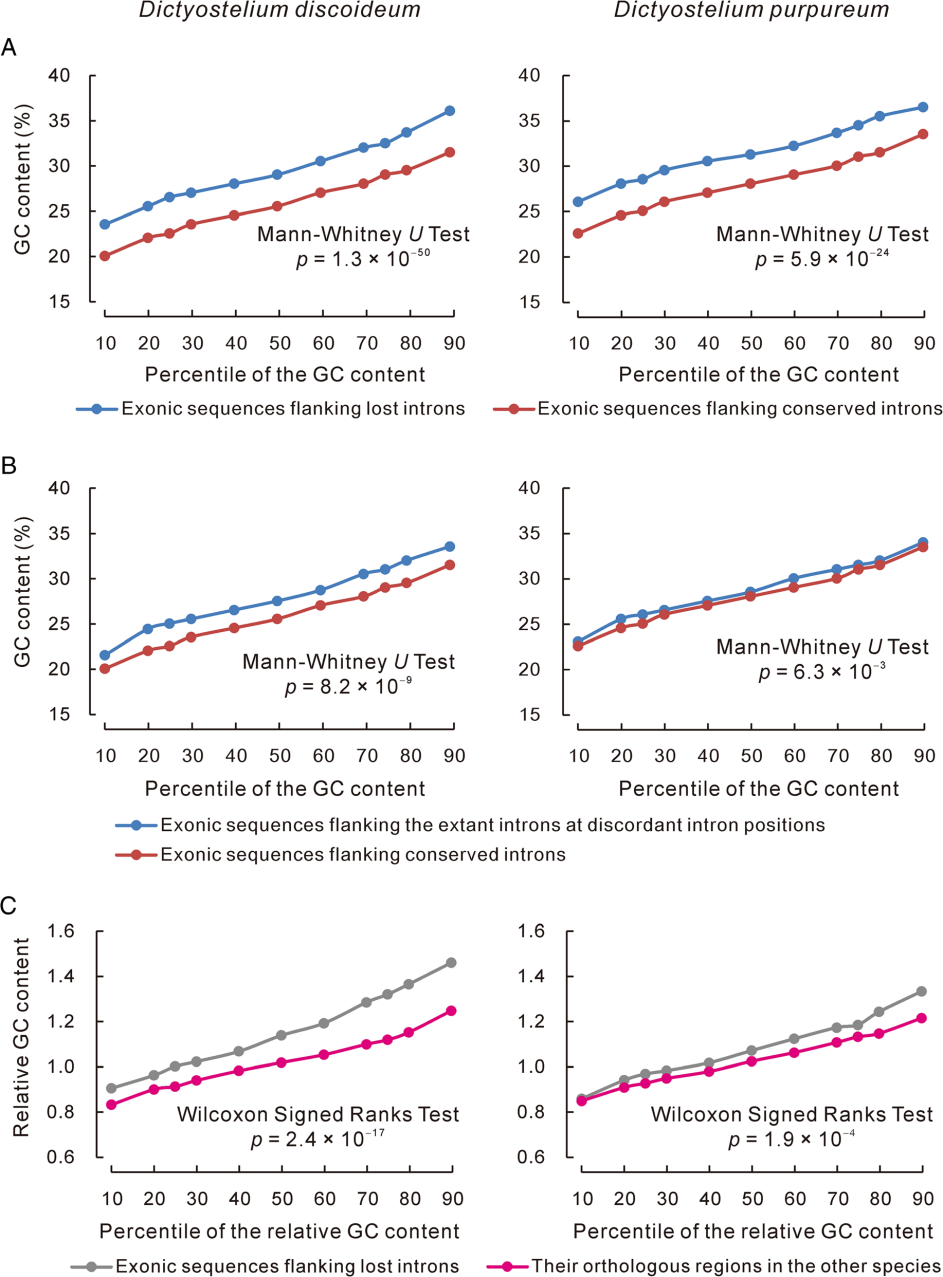
High GC content surrounding discordant intron positions. The difference in the distribution of GC or relative GC content between the two compared items could be perceived by comparing each percentile. **a** Exonic sequences flanking lost introns have significantly higher GC contents than the exonic sequences flanking conserved introns of the same species. **b** Exonic sequences flanking the extant introns at discordant intron positions have significantly higher GC contents than the conserved introns of the same species. **c** At discordant intron positions, exonic sequences flanking lost introns have significantly higher relative GC contents than the exonic sequences flanking extant introns of the other species. The relative GC content surrounding a discordant intron position was defined as the ratio of the GC content surrounding the position divided by the median value of the GC content surrounding the conserved introns of the same gene. A total of 407 lost intron positions in *Dictyostelium discoideum*, 178 lost intron positions in *Dictyostelium purpureum*, and 5,724 conserved intron positions were surveyed. The numbers of intron-lost genes surveyed were 201 and 109 for *Dictyostelium discoideum* and *Dictyostelium purpureum*, respectively. As the data are not normally distributed, we used nonparametric analyses in our comparisons of the data

It is widely accepted that gene conversion is asymmetrical. At G/C:A/T heterozygous sites, gene conversion tends to produce homozygous G/C more frequently than A/T [61, 62]. The GC-biased characteristic of gene conversion provides a new opportunity to test whether gene conversions were involved in the intron loss events. We first compared the GC content between the exonic sequences flanking conserved introns and those flanking lost introns. As shown in Fig. 5a, the exonic sequences flanking lost introns have significantly higher GC contents than those flanking conserved introns in both *Dictyostelium discoideum* and *Dictyostelium purpureum*.

Furthermore, the high GC content of the exonic sequences flanking lost introns can be explained in two ways. The first explanation is that intron losses preferentially occurred at gene conversion hotspots, where GC contents are higher regardless of whether intron losses have occurred. If this is the case, the exonic regions flanking the extant introns of discordant positions should also have higher GC contents than the exonic sequences flanking conserved introns. For example, gene *DDB_G0293580* of *Dictyostelium discoideum* has lost its second intron while its orthologous gene in *Dictyostelium purpureum* retained it, i.e. second intron of gene *DPU_G0070640*. If the intron was lost because of its presence at a gene conversion hotspot, the exonic sequence flanking the second intron of gene *DPU_G0070640* is expected to have higher GC content. As shown in Fig. 5b, this prediction has also been proved in both *Dictyostelium discoideum* and *Dictyostelium purpureum*. The second explanation is that the intron losses have increased the GC contents of nearby exonic sequences via biased gene conversion. If this is the case, the exonic sequences flanking lost introns should have higher GC contents than their orthologous regions flanking the unique introns. The global GC content differs significantly, however, between *Dictyostelium discoideum* and *Dictyostelium purpureum* (all coding sequences were compared, with median values of 28.35 % for *Dictyostelium discoideum* and 30.83 % for *Dictyostelium purpureum*, Mann–Whitney *U* test, *P =* 0). Therefore, we compared the GC content relative to the exonic sequences flanking conserved introns rather than the absolute values. As shown in Fig. 5c, the exonic sequences flanking lost introns have significantly higher relative GC contents than their orthologous regions. If gene conversion were to increase the GC content of nearby exonic sequences, there should be differences between the two participants of the gene conversion process. The source of nucleotide difference between cDNA and the genomic DNA might result from either transcription errors, reverse transcription errors [63], or even DNA replication errors if the gene conversion occurred after integration of the cDNA into a chromosome.

In the above comparisons, the GC contents of 100-bp regions from each side of the discordant and conserved intron positions were surveyed. Adjusting the surveyed sequence length to 50 bp and 200 bp yielded similar but less robust results. Longer sequences indicate that more nucleotides surveyed are likely to lie beyond the conversion tracts [64]. Counting the GC content only at 4-fold degenerate sites gave similar but slightly weaker results. Although 4-fold degenerate sites are more accurate in revealing the mutation rate, their limited numbers in coding sequences maximizes the stochastic noise in the calculation of the GC contents.

Because *Dictyostelium discoideum* and *Dictyostelium purpureum* diverged approximately 400 million years ago (Mya), it is possible that the changes in GC contents and the intron loss were independent from each other but are correlated by chance. For this reason, we analyzed the relationship between GC content and intron loss in six species that diverged from their sister species more recently: *Arabidopsis thaliana* (diverged from *Arabidopsis lyrata* 13 Mya [25]), *Caenorhabditis briggsae* (diverged from *Caenorhabditis remanei* 17.2 Mya [65]), *C. remanei* (diverged from *C. briggsae* 17.2 Mya [65]), *Rattus norvegicus* (diverged from *Mus musculus* 22.6 Mya [65]), *Brassica rapa* (diverged from *Thellungiella parvula* 30.8 Mya [25]), and *Drosophila willistoni* (diverged from *Drosophila melanogaster* 47.6 Mya [65]). The intron losses of *A. thaliana*, *R. norvegicus*, *B. rapa*, and *Drosophila willistoni* were obtained from previous publications [13, 16, 25, 28, 32, 66]. The number of intron losses in *A. lyrata*, *M. musculus*, *T. parvula* and *Drosophila melanogaster* are less than 50 in all cases. These results are not included in our analysis to minimize the stochastic noise. The intron losses of *C. remanei* and *C. briggsae* were identified in this study. The pattern of higher GC contents in the exonic sequences flanking lost introns have been confirmed in *A. thaliana*, *B. rapa*, *Drosophila willistoni*, *C. remanei* and *C. briggsae* (Additional file 3: Table S4). However, in *R. norvegicus*, the exonic sequences flanking lost introns do not have significantly higher GC contents than those flanking conserved introns. We observed that *R. norvegicus* has the smallest number of intron losses among the species analyzed in the present study (Additional file 3: Table S4). We suspect that stochastic noise might have covered the pattern in this particular species. In duplicating the patterns shown in Fig. 5b and c, more conflicting results were obtained among the six species (Additional file 3: Table S5-S6). In summary, we found a correlation between intron loss and higher GC contents of nearby exonic sequences. Introns were lost preferentially from GC-rich regions, which is a characteristic of frequent gene conversions [62].

### Intron gains occur less frequently than intron losses, and most are putative

**Fig. 6:**
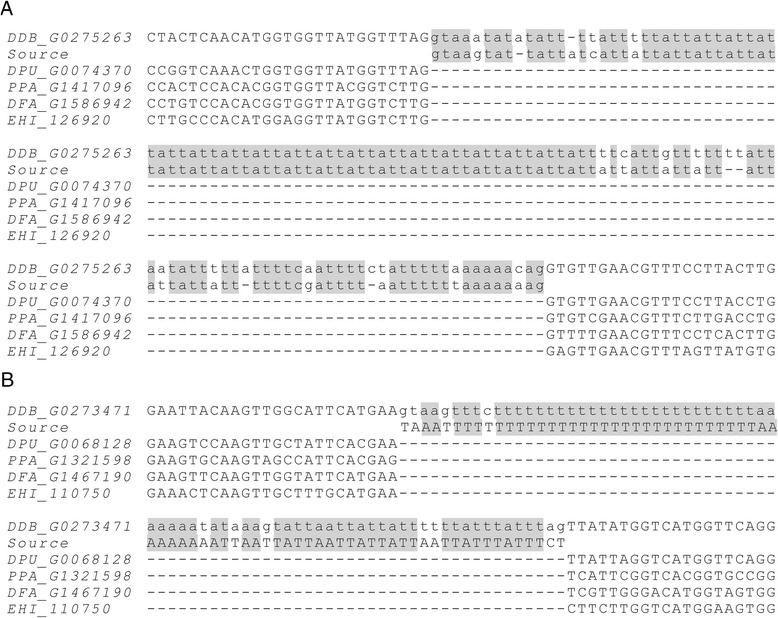
Two intron gains identified in *Dictyostelium discoideum*. **a** The second intron of gene *DDB_G0275263* was newly gained. The source sequence is the 5′ end of the third intron of gene *DDB_G0276095*. **b** The fourth intron of gene *DDB_G0273471* was newly gained. The source sequence is the 3′ end of the coding sequence and a 64 bp unannotated 3′ untranslated region of gene *DDB_G0267666.* Sequences upstream and downstream of the source sequences do not match the upstream and downstream exonic sequences of the novel introns, respectively. Therefore, these sequences are excluded from this figure. Abbreviations: *DDB*, *Dictyostelium discoideum*; *DPU*, *Dictyostelium purpureum*; *PPA*, *P. pallidum*; *DFA*, *Dictyostelium fasciculatum*; *EHI*, *E. histolytica*

Referring to previous publications reporting that recurrent intron losses are common in eukaryotic evolution [31, 58], we used a stringent criterion for the detection of putative intron gains: only the intron gains supported by all outgroup species were retained; that is, a unique intron in *Dictyostelium discoideum* was defined as a putative intron gain only when the absence of the intron at the position was confirmed in *Dictyostelium purpureum*, *P. pallidum*, *Dictyostelium fasciculatum*, and *E. histolytica*. In this way, 40 putative intron gains were detected in *Dictyostelium discoideum* and 58 in *Dictyostelium purpureum.* As noted by Logsdon et al. [67], a strong case for intron gain must be supported by a clear phylogeny and an identified source element of the gained intron. Sequences of the putatively gained introns were used as queries to search against the two *Dictyostelium* genomes and further against the nucleotide collection of NCBI using BLAST. The BLAST results were filtered with an E-value threshold of 10^−10^, a coverage threshold of 80 %, and a similarity threshold of 0.85. Source sequences have been found for only two new introns in *Dictyostelium discoideum*: the second intron of gene *DDB_G0275263* and the fourth intron of gene *DDB_G0273471* (Fig. 6). The source sequence of the second intron of gene *DDB_G0275263* is the 5′ end of the third intron of gene *DDB_G0276095* (transcript: *DDB0233372*), from its 5′ splice site (GT) to a position located 37 bp upstream of its 3′ end. We investigated whether this region is an alternatively spliced intron of gene *DDB_G0276095* using the transcriptome of *Dictyostelium discoideum*. No RNA-Seq reads could be mapped across the source region of gene *DDB_G0276095*. It seems that a cryptic 3′ splice site of the parental intron has been activated in the new intron. The source sequence of the fourth intron of gene *DDB_G0273471* is the 3′ end of the coding sequence and a 64 bp unannotated untranslated region of gene *DDB_G0267666* (transcript: *DDB0231999*). No short direct repeats exist at the boundaries of the two new introns, indicating that they were unlikely to have been gained during the repair of DNA double-strand breaks [68]. The source sequences are neither transposable elements, entire introns of the same (or other) genes, nor upstream or downstream sequences of the same genes. Therefore, none of the previously proposed models for intron gains [15] could account for the two cases we observed.

The proposed models of intron gains [15] clearly demonstrate that the source sequences are the key to identifying the mechanisms of intron gain. As *Dictyostelium discoideum* and *Dictyostelium purpureum* diverged 400 million years ago [49], the sequences of some early gained introns might have diverged from their source sequences to an extent too great to be detectable. However, if the intron gain rate remained steady throughout evolution, we would expect to find 12 instances of source sequences for the recently gained introns, e.g., within 50 million years. The difficulty in the identification of the source sequences of intron gains is common among studies, regardless of whether the studied species are distantly or closely related. For example, although *Drosophila persimilis* and *Drosophila pseudoobscura* diverged only two Mya, researchers failed to identify the source sequences for six of the seven introns gained after their divergence [13]. Even more astonishingly, researchers failed to identify the source sequences of 20 introns among 21 new introns that were gained only in certain local populations of *Daphnia pulex* [68]. One possibility is that some or even most of the “intron gains” are in fact those intron losses that were misidentified due to incomplete information on the phylogenetic background, as shown for *Caenorhabditis elegans* [31]. Due to the specialty of *Dictyostelium* genomes, we are confident that there are intron gains. By HGT, *Dictyostelium* had gained 16 genes from bacteria. As bacterial genes are definitely intronless, all the introns in these 16 genes were gained after the divergence of the Amoebozoa from the plants and animals. Within these genes, nine introns have been annotated in *Dictyostelium discoideum* and 19 introns have been annotated in *Dictyostelium purpureum*. Among them, there are seven pairs of introns at orthologous positions and 14 introns at discordant positions. The sequences of all these introns were used as queries to search against the two *Dictyostelium* genomes and further against the nucleotide collection of NCBI using BLAST. The BLAST results were filtered with an E-value threshold of 10^−10^, a coverage threshold of 80 %, and a similarity threshold of 0.85. Putative source sequences for two pairs of orthologous introns had been obtained. However, these source sequences have been rejected after alignment of them with the intron sequences and manual scrutiny. For the difficulty in finding intron sources, another possibility is that exogenous sequences such as viruses have contributed sequences for most intron gains but have not yet been covered in any genome sequencing projects [15]. In the future, surveying the metagenomic sequence data of the environmental samples obtained from the natural habitats of organisms with intron gains might lead to the identification of source sequences for additional intron gains and consequently reveal the mechanism of intron gain.

## Conclusions

*Dictyostelium* belongs to a supergroup, Amoebozoa, which diverged from animals and fungi very early in the evolutionary history of eukaryotes. In spite of this ancient divergence, our results indicate that its mechanism of intron loss is similar to that of animals and fungi. Most introns were lost in the process of gene conversion between the genomic DNA and cDNA reverse transcribed from mature mRNA.

## Methods

The genome sequences and annotation files of *Dictyostelium discoideum*, *Dictyostelium purpureum*, *P. pallidum*, and *Dictyostelium fasciculatum* were downloaded from DictyBase [69, 70] in March 2014, and those of *E. histolytica* (HM1IMSS, version 3.1) were downloaded from the AmoebaDB [71, 72]. We discarded genes with obvious annotation errors, such as those that did not contain coding sequences that were composed of multiples of three nucleotides or those that appeared to conflict with their protein sequences.

Using the BLAST reciprocal best hits with an E-value threshold of 10^−10^ and an identity threshold of 0.25, 7,503 pairs of one-to-one orthologous proteins were detected between *Dictyostelium discoideum* and *Dictyostelium purpureum*. Each pair of orthologous genes were independently aligned using ClustalW and MUSCLE [73, 74] with their default parameters. Sequences surrounding intron positions with low-quality alignments were discarded. Low quality was defined as a similarity between *Dictyostelium discoideum* and *Dictyostelium purpureum* within 45 bp at each side of less than 0.5, which is the first quartile of the similarities of all the aligned orthologous mRNAs. The consistent results obtained using ClustalW and MUSCLE were retained, including 6,432 conservative intron positions in 4,150 genes and 2,058 discordant intron positions in 1,605 groups of orthologous genes, with 679 unique introns in *Dictyostelium discoideum* and 1,379 unique introns in *Dictyostelium purpureum*. The orthologous genes in *P. pallidum*, *Dictyostelium fasciculatum*, and *E. histolytica* were detected and aligned using the same methods.

In most genome annotations, some errors are inevitable. The mis-annotation of exonic segments as introns would result in false-positive results of intron gain if the mis-annotated segments happened to be new insertions. Similarly, if a mis-annotated segment were deleted in another species, the simple deletion would be mis-recognized as an intron loss. Therefore, we re-annotated the introns at discordant positions using the transcriptome data of *Dictyostelium discoideum* (SRP023109), *Dictyostelium purpureum* (SRP001567), *P. pallidum* (SRP004023) and *E. histolytica* (SRP017935), which were downloaded from the Sequence Read Archive of NCBI [75]. The RNA-Seq reads were mapped to the genomes using TopHat version 2.0.5 with its default parameters [76]. Finally, we obtained 1,420 discordant intron positions in 1,170 groups of orthologous of genes.

The loss and gain of introns were distinguished using Dollop (version 3.69) [77]. An intron loss was defined when the total number of intron-presence-absence changes were minimized. An intron gain was defined only when the intron is definitely absent from the orthologous positions of all the outgroup species (Fig. 1). A precise loss was defined as an intron loss that did not cause any insertion and/or deletion in the flanking exonic sequences while an imprecise intron loss was companied by insertion and/or deletion in the flanking exonic sequences. Loss of adjacent introns were defined as the loss of two or more neighboring introns.

The version numbers of the genome sequences and source databases of *Arabidopsis thaliana*, *Arabidopsis lyrata*, *Brassica rapa*, *Thellungiella parvula*, *Drosophila willistoni*, *Drosophila melanogaster*, *Caenorhabditis briggsae*, *Caenorhabditis remanei*, *Caenorhabditis elegans*, *Caenorhabditis japonica*, *Rattus norvegicus*, *Mus musculus* are reported in Additional file 3: Table S7. The lost and conserved introns of *Brassica rapa*, *Arabidopsis thaliana* and *Rattus norvegicus* were retrieved from references [16, 25, 28, 32, 66], and the lost introns of *Drosophila willistoni* were those published in [13]. We updated the intron loss data with the latest versions of the genomes of *Drosophila willistoni* and *Drosophila melanogaster*.

Using the BLAST reciprocal best hits, 7,744 pairs of one-to-one orthologous proteins were detected between *Drosophila willistoni* and *Drosophila melanogaster*, and 12,613 between *C. briggsae* and *C. remanei*. Only hits with an E value below 10^−10^ and with identity higher than 25 % were considered. Each pair of orthologous genes was aligned using ClustalW and MUSCLE [4, 5] with their default parameters. Intron sites with no gap in the 10 bp alignment adjacent position (on both sides) were considered as “conserved” introns. In this way, we found 25,532 conserved introns between *Drosophila willistoni* and *Drosophila melanogaster* and 54,871 conserved introns between *C. briggsae* and *C. remanei*, which were used for further study.

Sequences surrounding discordant intron positions with low-quality alignments were discarded. The low quality was based on a similarity between *C. briggsae* and *C. remanei* within 45 bp at each side of less than 0.57, which is the first quartile of the similarities of all aligned orthologous mRNAs. Therefore, we obtained 4,827 discordant introns in 3,625 genes between the two *Caenorhabditis* species. We used *C. elegans* and *C. japonica* as related species to predict lost introns, only those introns existing in both *C. elegans* and *C. japonica* were used for further study. We then used the transcriptome data of *C. briggsae* (SRP034522), *C. remanei* (SRP040962) and *C. elegans* (SRP000401) to re-annotate the introns at discordant intron positions. Finally, we obtained 1,225 and 664 cases of intron losses in 1,026 and 572 genes of *C. briggsae* and *C. remanei*, respectively.

The threshold value of the similarity of coding sequences between *Drosophila willistoni* and *Drosophila melanogaster* was established as 0.5, which was lower than the first quartile (0.64). This value was used to best locate the corresponding lost introns found by Yenerall et al. [13]; in this manner, we identified 1,440 discordant intron sites. By corresponding to old versions of lost intron data, we found 93 cases of intron losses within 89 genes when using the latest genome information for *Drosophila willistoni*.

GO enrichment analysis was performed using GOTermFinder [72]. The GO annotations of *Dictyostelium discoideum* were used as the background dataset in this study. The total number of genes used in calculating the background distribution of GO terms was 12,098, and the threshold *P*-value of 0.01 was used in the identification of specific enrichments. The GO annotations are not been assigned to the genes of *Dictyostelium purpureum*. So they were represented by their orthologs in *Dictyostelium discoideum* in GO enrichment analysis. The 443 lost introns in *Dictyostelium discoideum* and 202 lost introns in *Dictyostelium purpureum* were mapped to 586 genes of *Dictyostelium discoideum*. These 586 intron-lost genes were compared with whole background sets. In same way, the putative gained introns of these two species were mapped to 86 intron-gained genes of *Dictyostelium discoideum*, which were compared with background sets for GO enrichments.

As almost all the data are not normally distributed, we used nonparametric analyses, like Mann–Whitney *U* test, in our comparisons of the data.

## Availability of supporting data

All supporting data are included as additional files in the form of Additional files 1, 2 and 3.

## Additional files

Additional file 1: Table S1. Presence and absence of introns at the discordant positions among the five genomes studied. (DOC 1161 kb) Additional file 2: Table S2. Results of gene ontology enrichment analysis. (DOC 203 kb) Additional file 3: Figure S1. Comparison of the intron sizes between Dictyostelium *discoideum* and Dictyostelium *purpureum*. All the introns annotated in these two genomes were compared, including 15,510 in Dictyostelium *discoideum* and 18,412 in Dictyostelium *purpureum*; Table S3. The abundance of repetitive sequences and introns in Dictyostelium *discoideum* and Dictyostelium *purpureum*. Table S4. Exonic sequences flanking lost introns have higher GC contents than those flanking conserved introns in most previously studied organisms; Table S5. At discordant intron positions, the relative GC content of exonic sequences flanking lost introns compared with the exonic sequences flanking extant introns of the sister species; Table S6. The GC content of exonic sequences flanking extant introns at discordant intron positions compared with those flanking conserved intron sites of the same species; Table S7. Version numbers and source databases of the plant and animal genomes used in this study. (DOC 540 kb)

## Abbreviations

GO: Gene Ontology
HGT: Horizontal gene transfer
Mya: Million years ago
NHEJ: Non-homologous end joining
SSR: Simple sequence repeat
