# Supplementary material for: Evaluation of the mechanisms of intron loss and gain in the social amoebae Dictyostelium
Source: BMC Evol Biol. 2015 Dec 18;15:286. doi: 10.1186/s12862-015-0567-y (PMC4683709; doi:10.1186/s12862-015-0567-y)
Supplement: Additional file 1: Table S1. — Presence and absence of introns at the discordant positions among the five genomes studied. (DOC 1161 kb) [file 12862_2015_567_MOESM1_ESM.doc]

Additional File 1 for

**Evaluation of the mechanisms of intron loss and gain in the social amoebae *Dictyostelium***

Ming-Yue Ma, Xun-Ru Che, Andrea Porceddu, and Deng-Ke Niu

Table S1. Presence and absence of introns at the discordant positions. Present, +; absent, -; uncertain, ?

| **Target transcript** | **Position (Nth base)** | ***D. discoideum*** |  | ***D. purpureum*** |  | ***P. pallidum*** |  | ***D. fasciculatum*** |  | ***E. histolytica*** |  |
| --- | --- | --- | --- | --- | --- | --- | --- | --- | --- | --- | --- |
| **The precise intron losses in *D. discoideum*** | | | | | | | | | | | |
| DDB0185052 | 1061 | DDB_G0276153 | - | DPU_G0067954 | + | PPA_G1320416 | + | DFA_G1526464 | - | EHI_004640 | - |
| DDB0185066 | 372 | DDB_G0275617 | - | DPU_G0072830 | + | PPA_G1354750 | - | DFA_G1522852 | + | EHI_112050 | - |
| DDB0185066 | 633 | DDB_G0275617 | - | DPU_G0072830 | + | PPA_G1354750 | ? | DFA_G1522852 | + | EHI_112050 | - |
| DDB0185096 | 2011 | DDB_G0276355 | - | DPU_G0067414 | + | PPA_G1364794 | + | DFA_G1482620 | + | ? | ? |
| DDB0185098 | 1295 | DDB_G0276143 | - | DPU_G0069672 | + | PPA_G1320980 | + | ? | ? | ? | ? |
| DDB0185175 | 175 | DDB_G0292558 | - | DPU_G0051394 | + | PPA_G1404564 | + | ? | ? | ? | ? |
| DDB0185194 | 2823 | DDB_G0274101 | - | DPU_G0063058 | + | PPA_G1403124 | + | DFA_G1472382 | - | ? | ? |
| DDB0185195 | 4503 | DDB_G0272032 | - | DPU_G0055640 | + | PPA_G1413328 | + | DFA_G1442540 | - | ? | ? |
| DDB0185198 | 4707 | DDB_G0274605 | - | DPU_G0068714 | + | PPA_G1366820 | + | ? | ? | ? | ? |
| DDB0185207 | 1720 | DDB_G0277401 | - | DPU_G0062362 | + | PPA_G1397308 | + | ? | ? | EHI_189850 | - |
| DDB0185212 | 592 | DDB_G0275449 | - | DPU_G0055780 | + | PPA_G1302156 | + | DFA_G1499168 | + | EHI_005070 | - |
| DDB0191172 | 426 | DDB_G0269246 | - | DPU_G0061100 | + | PPA_G1279610 | + | DFA_G1449686 | - | EHI_024420 | - |
| DDB0191203 | 766 | DDB_G0269174 | - | DPU_G0051726 | + | PPA_G1372330 | + | DFA_G1569794 | + | ? | ? |
| DDB0191251 | 221 | DDB_G0285433 | - | DPU_G0057216 | + | PPA_G1433040 | + | DFA_G1566212 | + | EHI_104470 | - |
| DDB0191263 | 298 | DDB_G0268618 | - | DPU_G0066348 | + | PPA_G1396038 | + | DFA_G1583900 | + | EHI_188070 | - |
| DDB0191272 | 105 | DDB_G0267414 | - | DPU_G0065074 | + | PPA_G1347770 | + | DFA_G1506526 | + | ? | ? |
| DDB0191272 | 2169 | DDB_G0267414 | - | DPU_G0065074 | + | PPA_G1347770 | + | DFA_G1506526 | - | ? | ? |
| DDB0191290 | 420 | DDB_G0267420 | - | DPU_G0054104 | + | ? | ? | DFA_G1536038 | + | ? | ? |
| DDB0191292 | 541 | DDB_G0267386 | - | DPU_G0061952 | + | PPA_G1381762 | + | DFA_G1557210 | + | ? | ? |
| DDB0191295 | 7280 | DDB_G0267472 | - | DPU_G0059300 | + | PPA_G1405688 | - | DFA_G1563900 | + | ? | ? |
| DDB0191309 | 3114 | DDB_G0275009 | - | DPU_G0075774 | + | PPA_G1425504 | + | DFA_G1578302 | - | ? | ? |
| DDB0191358 | 2211 | DDB_G0290901 | - | DPU_G0057172 | + | PPA_G1325556 | + | DFA_G1514844 | + | ? | ? |
| DDB0191363 | 80 | DDB_G0288373 | - | DPU_G0066994 | + | PPA_G1429206 | + | DFA_G1589480 | - | EHI_164510 | - |
| DDB0191386 | 894 | DDB_G0283621 | - | DPU_G0069566 | + | PPA_G1434702 | - | DFA_G1553882 | + | EHI_158240 | - |
| DDB0191388 | 2556 | DDB_G0284045 | - | DPU_G0059448 | + | PPA_G1367888 | + | DFA_G1488250 | + | ? | ? |
| DDB0191419 | 1622 | DDB_G0284473 | - | DPU_G0059150 | + | PPA_G1412362 | + | DFA_G1548146 | - | EHI_092600 | - |
| DDB0191425 | 3924 | DDB_G0269244 | - | DPU_G0061786 | + | PPA_G1269732 | + | DFA_G1581752 | + | ? | ? |
| DDB0191435 | 328 | DDB_G0289003 | - | DPU_G0064398 | + | PPA_G1398498 | + | DFA_G1494966 | + | EHI_177320 | - |
| DDB0191436 | 496 | DDB_G0289179 | - | DPU_G0058604 | + | ? | ? | DFA_G1479188 | + | EHI_177540 | - |
| DDB0191437 | 982 | DDB_G0287585 | - | DPU_G0060430 | + | PPA_G1423808 | + | DFA_G1545378 | - | EHI_035800 | - |
| DDB0191440 | 66 | DDB_G0287693 | - | DPU_G0067232 | + | PPA_G1307458 | + | DFA_G1577588 | + | EHI_147470 | - |
| DDB0191444 | 348 | DDB_G0286355 | - | DPU_G0056248 | + | PPA_G1283408 | + | DFA_G1525736 | + | EHI_110180 | - |
| DDB0191474 | 5188 | DDB_G0283081 | - | DPU_G0071394 | + | PPA_G1353930 | + | DFA_G1436658 | - | EHI_006130 | - |
| DDB0191475 | 703 | DDB_G0284039 | - | DPU_G0055452 | + | PPA_G1402018 | + | DFA_G1525540 | + | ? | ? |
| DDB0201558 | 628 | DDB_G0267454 | - | DPU_G0068816 | + | PPA_G1296078 | + | DFA_G1444474 | + | ? | ? |
| DDB0201559 | 44 | DDB_G0290963 | - | DPU_G0061224 | + | PPA_G1287054 | + | DFA_G1438408 | + | EHI_140230 | - |
| DDB0201639 | 531 | DDB_G0283603 | - | DPU_G0052764 | + | PPA_G1429040 | + | DFA_G1518160 | + | ? | ? |
| DDB0201656 | 364 | DDB_G0292736 | - | DPU_G0055284 | + | PPA_G1368214 | + | DFA_G1508448 | + | ? | ? |
| DDB0214811 | 740 | DDB_G0277911 | - | DPU_G0071604 | + | PPA_G1394132 | + | DFA_G1482096 | - | ? | ? |
| DDB0214817 | 907 | DDB_G0279911 | - | DPU_G0068716 | + | PPA_G1349374 | + | ? | ? | ? | ? |
| DDB0214819 | 1413 | DDB_G0271096 | - | DPU_G0062994 | + | PPA_G1293670 | - | DFA_G1484938 | + | EHI_064700 | - |
| DDB0214821 | 1096 | DDB_G0281337 | - | DPU_G0055130 | + | PPA_G1299930 | + | DFA_G1479652 | + | ? | ? |
| DDB0214828 | 2573 | DDB_G0278737 | - | DPU_G0052914 | + | PPA_G1347052 | + | DFA_G1477190 | - | ? | ? |
| DDB0214828 | 3073 | DDB_G0278737 | - | DPU_G0052914 | + | PPA_G1347052 | + | DFA_G1477190 | + | ? | ? |
| DDB0214834 | 2351 | DDB_G0279417 | - | DPU_G0065240 | + | PPA_G1357414 | + | DFA_G1545078 | + | ? | ? |
| DDB0214888 | 797 | DDB_G0282819 | - | DPU_G0056438 | + | PPA_G1394212 | + | ? | ? | EHI_039350 | - |
| DDB0214907 | 830 | DDB_G0279737 | - | DPU_G0062156 | + | PPA_G1356480 | + | DFA_G1446336 | - | ? | ? |
| DDB0214956 | 535 | DDB_G0282363 | - | DPU_G0066466 | + | PPA_G1332018 | + | DFA_G1518330 | + | EHI_086080 | - |
| DDB0214958 | 1117 | DDB_G0280041 | - | DPU_G0069348 | + | PPA_G1321792 | + | DFA_G1540906 | - | ? | ? |
| DDB0214985 | 1423 | DDB_G0277865 | - | DPU_G0059552 | + | PPA_G1394868 | + | DFA_G1486258 | + | ? | ? |
| DDB0215362 | 676 | DDB_G0293528 | - | DPU_G0056324 | + | ? | ? | DFA_G1563178 | + | EHI_177400 | - |
| DDB0215368 | 1421 | DDB_G0293008 | - | DPU_G0062754 | + | ? | ? | DFA_G1525614 | + | ? | ? |
| DDB0215368 | 639 | DDB_G0293008 | - | DPU_G0062754 | + | ? | ? | DFA_G1525614 | + | ? | ? |
| DDB0215377 | 346 | DDB_G0280401 | - | DPU_G0054850 | + | PPA_G1380124 | + | DFA_G1566740 | + | ? | ? |
| DDB0216241 | 1256 | DDB_G0278457 | - | DPU_G0064200 | + | ? | ? | DFA_G1591596 | + | EHI_002790 | - |
| DDB0216286 | 84 | DDB_G0291362 | - | DPU_G0051878 | + | PPA_G1434128 | + | DFA_G1588800 | + | ? | ? |
| DDB0216288 | 1060 | DDB_G0282339 | - | DPU_G0064478 | + | PPA_G1298360 | + | DFA_G1567564 | + | ? | ? |
| DDB0216293 | 419 | DDB_G0277199 | - | DPU_G0055966 | + | PPA_G1346012 | + | DFA_G1477054 | + | EHI_125350 | - |
| DDB0216299 | 54 | DDB_G0278241 | - | DPU_G0071684 | + | PPA_G1408698 | + | DFA_G1445804 | + | ? | ? |
| DDB0216308 | 83 | DDB_G0271550 | - | DPU_G0059860 | + | PPA_G1326014 | + | ? | ? | ? | ? |
| DDB0216316 | 834 | DDB_G0271514 | - | DPU_G0064310 | + | PPA_G1430664 | - | DFA_G1511452 | + | ? | ? |
| DDB0216317 | 199 | DDB_G0287707 | - | DPU_G0058478 | + | PPA_G1406370 | + | DFA_G1490970 | + | EHI_053450 | - |
| DDB0216321 | 1380 | DDB_G0278367 | - | DPU_G0072286 | + | PPA_G1368796 | + | DFA_G1526090 | + | ? | ? |
| DDB0216323 | 1239 | DDB_G0281895 | - | DPU_G0058454 | + | PPA_G1332204 | + | ? | ? | ? | ? |
| DDB0216377 | 1988 | DDB_G0274593 | - | DPU_G0074488 | + | PPA_G1354800 | + | DFA_G1444624 | + | ? | ? |
| DDB0216387 | 1199 | DDB_G0290859 | - | DPU_G0053304 | + | PPA_G1287810 | + | DFA_G1439606 | + | ? | ? |
| DDB0216387 | 939 | DDB_G0290859 | - | DPU_G0053304 | + | PPA_G1287810 | + | DFA_G1439606 | + | ? | ? |
| DDB0216421 | 766 | DDB_G0269778 | - | DPU_G0063408 | + | PPA_G1302778 | + | DFA_G1539678 | + | EHI_093570 | - |
| DDB0220000 | 2117 | DDB_G0275289 | - | DPU_G0075776 | + | PPA_G1296140 | + | DFA_G1578284 | + | ? | ? |
| DDB0220010 | 1047 | DDB_G0279405 | - | DPU_G0063552 | + | PPA_G1368468 | + | ? | ? | ? | ? |
| DDB0220010 | 1360 | DDB_G0279405 | - | DPU_G0063552 | + | PPA_G1368468 | + | ? | ? | ? | ? |
| DDB0220033 | 5022 | DDB_G0278965 | - | DPU_G0073834 | + | PPA_G1391384 | + | ? | ? | ? | ? |
| DDB0220086 | 1662 | DDB_G0288705 | - | DPU_G0056250 | + | PPA_G1346986 | + | DFA_G1540258 | + | ? | ? |
| DDB0220500 | 4250 | DDB_G0275391 | - | DPU_G0075852 | + | PPA_G1421814 | - | DFA_G1447782 | + | ? | ? |
| DDB0220679 | 1077 | DDB_G0278929 | - | DPU_G0067268 | + | PPA_G1273090 | + | DFA_G1532564 | + | EHI_152110 | - |
| DDB0220704 | 4063 | DDB_G0281521 | - | DPU_G0074216 | + | PPA_G1407810 | + | DFA_G1583734 | + | ? | ? |
| DDB0220706 | 561 | DDB_G0284411 | - | DPU_G0055002 | + | PPA_G1367172 | + | DFA_G1569362 | + | ? | ? |
| DDB0229364 | 494 | DDB_G0277165 | - | DPU_G0060118 | + | PPA_G1398176 | + | DFA_G1500204 | - | EHI_110010 | - |
| DDB0229365 | 260 | DDB_G0268760 | - | DPU_G0062272 | + | PPA_G1376450 | + | DFA_G1576732 | + | ? | ? |
| DDB0229377 | 90 | DDB_G0289069 | - | DPU_G0056396 | + | PPA_G1385176 | + | DFA_G1467410 | + | ? | ? |
| DDB0229382 | 63 | DDB_G0267962 | - | DPU_G0070418 | + | PPA_G1419186 | + | DFA_G1555136 | - | ? | ? |
| DDB0229406 | 132 | DDB_G0291738 | - | DPU_G0064676 | + | PPA_G1318102 | + | ? | ? | ? | ? |
| DDB0229408 | 823 | DDB_G0273865 | - | DPU_G0065958 | + | PPA_G1301998 | + | DFA_G1517340 | - | ? | ? |
| DDB0229410 | 1594 | DDB_G0274409 | - | DPU_G0074510 | + | PPA_G1388314 | - | DFA_G1519038 | + | ? | ? |
| DDB0229410 | 1657 | DDB_G0274409 | - | DPU_G0074510 | + | PPA_G1388314 | + | DFA_G1519038 | - | ? | ? |
| DDB0229428 | 662 | DDB_G0273721 | - | DPU_G0064748 | + | PPA_G1423054 | + | DFA_G1441702 | + | EHI_108670 | - |
| DDB0229847 | 6399 | DDB_G0267686 | - | DPU_G0058284 | + | PPA_G1286482 | + | DFA_G1551560 | + | ? | ? |
| DDB0229866 | 508 | DDB_G0271402 | - | DPU_G0069936 | + | PPA_G1277178 | + | DFA_G1544472 | - | ? | ? |
| DDB0229939 | 1062 | DDB_G0285463 | - | DPU_G0063812 | + | PPA_G1361630 | + | DFA_G1537418 | + | ? | ? |
| DDB0229963 | 1965 | DDB_G0272254 | - | DPU_G0056114 | + | PPA_G1338262 | + | DFA_G1579932 | + | ? | ? |
| DDB0229967 | 1874 | DDB_G0287853 | - | DPU_G0052150 | + | PPA_G1273304 | + | DFA_G1458096 | - | ? | ? |
| DDB0229980 | 716 | DDB_G0277381 | - | DPU_G0073460 | + | PPA_G1325018 | + | DFA_G1437106 | - | ? | ? |
| DDB0230019 | 1757 | DDB_G0278521 | - | DPU_G0063252 | + | PPA_G1277668 | + | DFA_G1547338 | + | ? | ? |
| DDB0230038 | 1971 | DDB_G0280131 | - | DPU_G0051656 | + | PPA_G1314220 | + | DFA_G1566026 | + | ? | ? |
| DDB0230038 | 2206 | DDB_G0280131 | - | DPU_G0051656 | + | PPA_G1314220 | ? | DFA_G1566026 | + | ? | ? |
| DDB0230053 | 930 | DDB_G0269950 | - | DPU_G0062622 | + | PPA_G1388844 | + | DFA_G1485160 | - | ? | ? |
| DDB0230069 | 1097 | DDB_G0279211 | - | DPU_G0067610 | + | PPA_G1310718 | + | DFA_G1449170 | + | ? | ? |
| DDB0230086 | 704 | DDB_G0288145 | - | DPU_G0066692 | + | PPA_G1376706 | - | DFA_G1566054 | + | ? | ? |
| DDB0230112 | 482 | DDB_G0293580 | - | DPU_G0070640 | + | PPA_G1400294 | + | DFA_G1543036 | - | EHI_140240 | - |
| DDB0230127 | 546 | DDB_G0283151 | - | DPU_G0061752 | + | PPA_G1393818 | + | ? | ? | ? | ? |
| DDB0230134 | 390 | DDB_G0292632 | - | DPU_G0073802 | + | PPA_G1307912 | + | DFA_G1481020 | + | ? | ? |
| DDB0230139 | 174 | DDB_G0278283 | - | DPU_G0060198 | + | PPA_G1292682 | - | DFA_G1552478 | + | ? | ? |
| DDB0230152 | 413 | DDB_G0283741 | - | DPU_G0059156 | + | PPA_G1268752 | - | DFA_G1441188 | + | EHI_152570 | - |
| DDB0230195 | 366 | DDB_G0281797 | - | DPU_G0066882 | + | PPA_G1353568 | + | DFA_G1523634 | + | ? | ? |
| DDB0230204 | 183 | DDB_G0287735 | - | DPU_G0075448 | + | ? | ? | DFA_G1567094 | + | ? | ? |
| DDB0230210 | 1582 | DDB_G0277245 | - | DPU_G0070526 | + | PPA_G1306766 | + | ? | ? | EHI_126080 | - |
| DDB0230997 | 733 | DDB_G0272360 | - | DPU_G0065044 | + | PPA_G1317124 | + | DFA_G1591238 | - | ? | ? |
| DDB0231036 | 533 | DDB_G0281663 | - | DPU_G0052892 | + | PPA_G1385072 | + | DFA_G1436088 | + | ? | ? |
| DDB0231090 | 2868 | DDB_G0278321 | - | DPU_G0059212 | + | PPA_G1372428 | + | DFA_G1521382 | - | EHI_011790 | - |
| DDB0231151 | 477 | DDB_G0286389 | - | DPU_G0056874 | + | PPA_G1430466 | + | ? | ? | EHI_167050 | - |
| DDB0231199 | 4271 | DDB_G0282895 | - | DPU_G0066116 | + | PPA_G1286024 | + | DFA_G1445076 | + | EHI_006870 | - |
| DDB0231220 | 1715 | DDB_G0284747 | - | DPU_G0057342 | + | PPA_G1291016 | + | DFA_G1575946 | + | EHI_135130 | - |
| DDB0231246 | 468 | DDB_G0274567 | - | DPU_G0061486 | + | PPA_G1304628 | + | DFA_G1584604 | - | ? | ? |
| DDB0231334 | 845 | DDB_G0268100 | - | DPU_G0074272 | + | PPA_G1389476 | + | ? | ? | ? | ? |
| DDB0231371 | 1265 | DDB_G0272224 | - | DPU_G0055670 | + | PPA_G1322310 | + | DFA_G1528904 | ? | ? | ? |
| DDB0231371 | 783 | DDB_G0272224 | - | DPU_G0055670 | + | PPA_G1322310 | + | DFA_G1528904 | + | ? | ? |
| DDB0231380 | 636 | DDB_G0277455 | - | DPU_G0059798 | + | PPA_G1395470 | + | DFA_G1475536 | + | ? | ? |
| DDB0231417 | 275 | DDB_G0284697 | - | DPU_G0058596 | + | PPA_G1354278 | + | DFA_G1508008 | + | ? | ? |
| DDB0231469 | 259 | DDB_G0270658 | - | DPU_G0066066 | + | PPA_G1327496 | + | DFA_G1574758 | + | ? | ? |
| DDB0231503 | 120 | DDB_G0270028 | - | DPU_G0066574 | + | PPA_G1324168 | + | DFA_G1568574 | - | ? | ? |
| DDB0231508 | 2020 | DDB_G0277949 | - | DPU_G0055620 | + | PPA_G1294258 | + | DFA_G1576672 | + | ? | ? |
| DDB0231508 | 2397 | DDB_G0277949 | - | DPU_G0055620 | + | PPA_G1294258 | + | DFA_G1576672 | ? | ? | ? |
| DDB0231514 | 609 | DDB_G0282611 | - | DPU_G0069956 | + | PPA_G1294528 | + | DFA_G1589210 | + | ? | ? |
| DDB0231594 | 852 | DDB_G0291440 | - | DPU_G0058096 | + | PPA_G1396698 | + | DFA_G1594606 | + | ? | ? |
| DDB0231612 | 369 | DDB_G0278259 | - | DPU_G0054962 | + | PPA_G1395458 | + | DFA_G1590092 | + | ? | ? |
| DDB0231616 | 135 | DDB_G0287231 | - | DPU_G0053730 | + | PPA_G1366300 | + | ? | ? | ? | ? |
| DDB0231723 | 2045 | DDB_G0291095 | - | DPU_G0061448 | + | ? | ? | DFA_G1493322 | + | ? | ? |
| DDB0231789 | 1228 | DDB_G0275711 | - | DPU_G0059912 | + | PPA_G1330862 | + | DFA_G1451822 | + | ? | ? |
| DDB0231827 | 158 | DDB_G0290185 | - | DPU_G0054366 | + | PPA_G1407292 | + | ? | ? | ? | ? |
| DDB0231840 | 1066 | DDB_G0293768 | - | DPU_G0062344 | + | PPA_G1289004 | + | ? | ? | ? | ? |
| DDB0232002 | 328 | DDB_G0286303 | - | DPU_G0068350 | + | PPA_G1351444 | - | DFA_G1454662 | + | ? | ? |
| DDB0232003 | 152 | DDB_G0282529 | - | DPU_G0056500 | + | PPA_G1359282 | + | DFA_G1449798 | + | ? | ? |
| DDB0232050 | 965 | DDB_G0282265 | - | DPU_G0059498 | + | PPA_G1285102 | + | DFA_G1487760 | - | EHI_125660 | - |
| DDB0232063 | 85 | DDB_G0290123 | - | DPU_G0055554 | + | PPA_G1333870 | + | DFA_G1475232 | + | ? | ? |
| DDB0232074 | 566 | DDB_G0267500 | - | DPU_G0074416 | + | PPA_G1426400 | + | DFA_G1575932 | - | ? | ? |
| DDB0232111 | 4467 | DDB_G0291536 | - | DPU_G0069738 | + | PPA_G1388606 | + | DFA_G1529816 | + | ? | ? |
| DDB0232114 | 508 | DDB_G0275815 | - | DPU_G0070940 | + | PPA_G1345994 | + | DFA_G1437772 | - | ? | ? |
| DDB0232144 | 2143 | DDB_G0280481 | - | DPU_G0060186 | + | PPA_G1309518 | + | DFA_G1574462 | + | ? | ? |
| DDB0232216 | 858 | DDB_G0293706 | - | DPU_G0062702 | + | PPA_G1397050 | + | ? | ? | EHI_148520 | - |
| DDB0232220 | 786 | DDB_G0276103 | - | DPU_G0067808 | + | PPA_G1418230 | + | DFA_G1474254 | + | ? | ? |
| DDB0232221 | 2688 | DDB_G0278545 | - | DPU_G0075678 | + | PPA_G1411652 | + | DFA_G1554388 | + | ? | ? |
| DDB0232233 | 47 | DDB_G0286027 | - | DPU_G0072214 | + | PPA_G1301188 | + | DFA_G1519588 | + | EHI_167150 | - |
| DDB0232247 | 405 | DDB_G0289033 | - | DPU_G0071250 | + | PPA_G1329848 | - | DFA_G1581632 | + | ? | ? |
| DDB0232248 | 1572 | DDB_G0288031 | - | DPU_G0053124 | + | PPA_G1320068 | - | DFA_G1440198 | + | ? | ? |
| DDB0232272 | 762 | DDB_G0273727 | - | DPU_G0065010 | + | PPA_G1396782 | + | DFA_G1496992 | + | ? | ? |
| DDB0232356 | 1243 | DDB_G0275623 | - | DPU_G0052972 | + | PPA_G1379994 | + | DFA_G1555280 | + | EHI_187720 | - |
| DDB0232360 | 1178 | DDB_G0275999 | - | DPU_G0067204 | + | PPA_G1347892 | + | DFA_G1568492 | + | ? | ? |
| DDB0232960 | 2985 | DDB_G0281683 | - | DPU_G0066398 | + | PPA_G1304078 | + | DFA_G1483564 | - | ? | ? |
| DDB0232977 | 1799 | DDB_G0287953 | - | DPU_G0075406 | + | PPA_G1377446 | - | DFA_G1449278 | + | EHI_049680 | - |
| DDB0232994 | 1848 | DDB_G0281315 | - | DPU_G0064864 | + | ? | ? | DFA_G1508594 | + | EHI_182600 | - |
| DDB0233049 | 831 | DDB_G0284345 | - | DPU_G0068498 | + | PPA_G1269478 | + | DFA_G1438614 | + | ? | ? |
| DDB0233070 | 166 | DDB_G0285901 | - | DPU_G0062328 | + | PPA_G1309050 | + | DFA_G1437038 | + | ? | ? |
| DDB0233070 | 1913 | DDB_G0285901 | - | DPU_G0062328 | + | PPA_G1309050 | + | DFA_G1437038 | + | ? | ? |
| DDB0233070 | 787 | DDB_G0285901 | - | DPU_G0062328 | + | PPA_G1309050 | + | DFA_G1437038 | - | ? | ? |
| DDB0233098 | 637 | DDB_G0293588 | - | DPU_G0072922 | + | PPA_G1425402 | + | DFA_G1526178 | + | ? | ? |
| DDB0233173 | 1127 | DDB_G0269424 | - | DPU_G0060288 | + | ? | ? | DFA_G1536940 | + | ? | ? |
| DDB0233205 | 778 | DDB_G0277231 | - | DPU_G0059254 | + | PPA_G1319670 | - | DFA_G1552016 | + | ? | ? |
| DDB0233285 | 2105 | DDB_G0274775 | - | DPU_G0070350 | + | PPA_G1409948 | + | ? | ? | EHI_040420 | - |
| DDB0233302 | 932 | DDB_G0292904 | - | DPU_G0054794 | + | PPA_G1286234 | + | DFA_G1550882 | - | ? | ? |
| DDB0233316 | 2427 | DDB_G0278147 | - | DPU_G0055058 | + | PPA_G1302388 | - | DFA_G1458230 | + | ? | ? |
| DDB0233339 | 3864 | DDB_G0280209 | - | DPU_G0075700 | + | PPA_G1342478 | + | DFA_G1477110 | + | ? | ? |
| DDB0233373 | 1107 | DDB_G0276251 | - | DPU_G0055074 | + | ? | ? | DFA_G1587494 | + | ? | ? |
| DDB0233401 | 418 | DDB_G0291015 | - | DPU_G0061236 | + | PPA_G1426268 | + | DFA_G1480414 | + | ? | ? |
| DDB0233422 | 1063 | DDB_G0274555 | - | DPU_G0061496 | + | PPA_G1371372 | - | DFA_G1474964 | + | ? | ? |
| DDB0233432 | 2229 | DDB_G0288501 | - | DPU_G0066788 | + | PPA_G1272524 | + | DFA_G1455944 | - | ? | ? |
| DDB0233434 | 1386 | DDB_G0286219 | - | DPU_G0053196 | + | PPA_G1390728 | + | DFA_G1575046 | + | EHI_178810 | - |
| DDB0233442 | 2698 | DDB_G0288873 | - | DPU_G0058242 | + | PPA_G1433412 | + | DFA_G1547462 | + | EHI_103660 | - |
| DDB0233447 | 600 | DDB_G0283661 | - | DPU_G0058654 | + | PPA_G1384880 | - | DFA_G1566198 | + | EHI_175030 | - |
| DDB0233466 | 1343 | DDB_G0290493 | - | DPU_G0064668 | + | PPA_G1401640 | + | DFA_G1456786 | + | ? | ? |
| DDB0233467 | 442 | DDB_G0284739 | - | DPU_G0053642 | + | PPA_G1416358 | + | DFA_G1472094 | - | ? | ? |
| DDB0233475 | 1404 | DDB_G0291085 | - | DPU_G0059622 | + | PPA_G1335178 | + | DFA_G1593452 | + | ? | ? |
| DDB0233493 | 1647 | DDB_G0274889 | - | DPU_G0066958 | + | PPA_G1330230 | + | DFA_G1467700 | - | ? | ? |
| DDB0233494 | 2631 | DDB_G0284501 | - | DPU_G0059456 | + | PPA_G1319452 | + | DFA_G1579586 | + | ? | ? |
| DDB0233495 | 3319 | DDB_G0278417 | - | DPU_G0057146 | + | PPA_G1394042 | + | DFA_G1575292 | + | ? | ? |
| DDB0233506 | 988 | DDB_G0270750 | - | DPU_G0060812 | + | PPA_G1392284 | - | DFA_G1588134 | + | ? | ? |
| DDB0233537 | 1294 | DDB_G0278353 | - | DPU_G0072330 | + | PPA_G1268736 | + | DFA_G1516328 | + | ? | ? |
| DDB0233542 | 1316 | DDB_G0269054 | - | DPU_G0066288 | + | PPA_G1389304 | + | DFA_G1591010 | + | ? | ? |
| DDB0233550 | 449 | DDB_G0291734 | - | DPU_G0057274 | + | PPA_G1384934 | + | DFA_G1578838 | + | ? | ? |
| DDB0233558 | 1282 | DDB_G0291063 | - | DPU_G0053310 | + | PPA_G1403522 | + | DFA_G1463696 | - | ? | ? |
| DDB0233620 | 442 | DDB_G0270866 | - | DPU_G0060242 | + | ? | ? | DFA_G1471680 | + | ? | ? |
| DDB0233621 | 3134 | DDB_G0275239 | - | DPU_G0053332 | + | PPA_G1342974 | - | DFA_G1445640 | + | ? | ? |
| DDB0233622 | 6108 | DDB_G0293084 | - | DPU_G0056004 | + | PPA_G1347700 | + | DFA_G1571226 | + | ? | ? |
| DDB0233624 | 3777 | DDB_G0268888 | - | DPU_G0070224 | + | PPA_G1298100 | + | DFA_G1524378 | + | ? | ? |
| DDB0233624 | 4374 | DDB_G0268888 | - | DPU_G0070224 | + | PPA_G1298100 | + | DFA_G1524378 | + | ? | ? |
| DDB0233625 | 2200 | DDB_G0284725 | - | DPU_G0061656 | + | PPA_G1371208 | - | DFA_G1489848 | + | ? | ? |
| DDB0233625 | 2422 | DDB_G0284725 | - | DPU_G0061656 | + | PPA_G1371208 | - | DFA_G1489848 | + | ? | ? |
| DDB0233627 | 1465 | DDB_G0292004 | - | DPU_G0060636 | + | PPA_G1420610 | + | DFA_G1584106 | + | ? | ? |
| DDB0233651 | 457 | DDB_G0290307 | - | DPU_G0068270 | + | ? | ? | DFA_G1565234 | + | ? | ? |
| DDB0233657 | 419 | DDB_G0286695 | - | DPU_G0056534 | + | PPA_G1386512 | + | DFA_G1557692 | + | ? | ? |
| DDB0233685 | 326 | DDB_G0289447 | - | DPU_G0056360 | + | PPA_G1327656 | + | DFA_G1557536 | + | ? | ? |
| DDB0233707 | 1904 | DDB_G0286645 | - | DPU_G0055184 | + | PPA_G1338892 | + | DFA_G1562604 | + | ? | ? |
| DDB0233725 | 1511 | DDB_G0283057 | - | DPU_G0067310 | + | PPA_G1349598 | + | DFA_G1546290 | - | ? | ? |
| DDB0233763 | 2257 | DDB_G0271750 | - | DPU_G0053944 | + | PPA_G1355966 | + | DFA_G1595042 | + | ? | ? |
| DDB0233768 | 6403 | DDB_G0291115 | - | DPU_G0062418 | + | PPA_G1380970 | + | DFA_G1596638 | - | ? | ? |
| DDB0233777 | 191 | DDB_G0284403 | - | DPU_G0070692 | + | PPA_G1373512 | + | DFA_G1462142 | + | EHI_081770 | - |
| DDB0233777 | 259 | DDB_G0284403 | - | DPU_G0070692 | + | PPA_G1373512 | + | DFA_G1462142 | - | EHI_081770 | - |
| DDB0233815 | 611 | DDB_G0290757 | - | DPU_G0057208 | + | PPA_G1328876 | + | DFA_G1517482 | + | ? | ? |
| DDB0233831 | 715 | DDB_G0267536 | - | DPU_G0053562 | + | PPA_G1298146 | - | DFA_G1566752 | + | ? | ? |
| DDB0233834 | 391 | DDB_G0287741 | - | DPU_G0075270 | + | PPA_G1389668 | + | DFA_G1461808 | + | ? | ? |
| DDB0233837 | 208 | DDB_G0287737 | - | DPU_G0069122 | + | ? | ? | DFA_G1445274 | + | ? | ? |
| DDB0233871 | 630 | DDB_G0283899 | - | DPU_G0068110 | + | PPA_G1365252 | - | DFA_G1565750 | + | ? | ? |
| DDB0233872 | 1044 | DDB_G0293654 | - | DPU_G0056322 | + | PPA_G1425202 | - | DFA_G1470994 | + | ? | ? |
| DDB0233888 | 708 | DDB_G0293646 | - | DPU_G0062352 | + | ? | ? | DFA_G1483014 | + | ? | ? |
| DDB0233891 | 872 | DDB_G0288807 | - | DPU_G0064094 | + | PPA_G1353882 | + | DFA_G1543588 | + | ? | ? |
| DDB0233958 | 936 | DDB_G0282593 | - | DPU_G0058506 | + | PPA_G1310468 | + | DFA_G1586002 | - | ? | ? |
| DDB0233962 | 2754 | DDB_G0284833 | - | DPU_G0057344 | + | PPA_G1349572 | + | DFA_G1530936 | + | ? | ? |
| DDB0233963 | 2884 | DDB_G0287881 | - | DPU_G0065644 | + | PPA_G1424822 | + | DFA_G1595170 | + | ? | ? |
| DDB0233971 | 862 | DDB_G0279991 | - | DPU_G0059426 | + | PPA_G1287758 | + | DFA_G1585880 | + | ? | ? |
| DDB0234023 | 614 | DDB_G0285295 | - | DPU_G0069854 | + | PPA_G1363774 | + | DFA_G1498628 | + | ? | ? |
| DDB0234042 | 6891 | DDB_G0275861 | - | DPU_G0060994 | + | PPA_G1392578 | + | ? | ? | ? | ? |
| DDB0234057 | 356 | DDB_G0287993 | - | DPU_G0059120 | + | PPA_G1404844 | + | DFA_G1553920 | - | ? | ? |
| DDB0234073 | 2626 | DDB_G0285393 | - | DPU_G0071620 | + | PPA_G1383106 | + | ? | ? | ? | ? |
| DDB0234111 | 1123 | DDB_G0276261 | - | DPU_G0072518 | + | PPA_G1426874 | + | DFA_G1464440 | + | ? | ? |
| DDB0234129 | 510 | DDB_G0294631 | - | DPU_G0070042 | + | PPA_G1294212 | + | ? | ? | ? | ? |
| DDB0234135 | 452 | DDB_G0278141 | - | DPU_G0068280 | + | PPA_G1379340 | + | DFA_G1472908 | + | ? | ? |
| DDB0234137 | 395 | DDB_G0270754 | - | DPU_G0075638 | + | PPA_G1291218 | + | DFA_G1505784 | - | ? | ? |
| DDB0234144 | 1174 | DDB_G0286669 | - | DPU_G0051538 | + | PPA_G1294124 | - | DFA_G1457132 | + | ? | ? |
| DDB0234147 | 1361 | DDB_G0282397 | - | DPU_G0068156 | + | PPA_G1341666 | + | DFA_G1516468 | + | ? | ? |
| DDB0234155 | 1876 | DDB_G0270990 | - | DPU_G0067394 | + | PPA_G1348418 | + | DFA_G1471236 | - | ? | ? |
| DDB0234170 | 156 | DDB_G0267766 | - | DPU_G0061966 | + | PPA_G1362522 | + | DFA_G1591612 | - | ? | ? |
| DDB0234205 | 3980 | DDB_G0286545 | - | DPU_G0057916 | + | PPA_G1321416 | + | DFA_G1485946 | + | ? | ? |
| DDB0234206 | 1331 | DDB_G0270396 | - | DPU_G0057394 | + | PPA_G1360106 | + | DFA_G1477344 | - | ? | ? |
| DDB0234229 | 2197 | DDB_G0293772 | - | DPU_G0062348 | + | PPA_G1311246 | + | DFA_G1510370 | + | ? | ? |
| DDB0234230 | 1497 | DDB_G0293496 | - | DPU_G0061364 | + | PPA_G1414062 | + | DFA_G1492782 | + | ? | ? |
| DDB0234230 | 523 | DDB_G0293496 | - | DPU_G0061364 | + | PPA_G1414062 | + | DFA_G1492782 | + | ? | ? |
| DDB0234230 | 767 | DDB_G0293496 | - | DPU_G0061364 | + | PPA_G1414062 | + | DFA_G1492782 | + | ? | ? |
| DDB0234234 | 807 | DDB_G0281641 | - | DPU_G0054808 | + | PPA_G1284448 | + | ? | ? | ? | ? |
| DDB0234260 | 1858 | DDB_G0292436 | - | DPU_G0052216 | + | PPA_G1412584 | + | DFA_G1575700 | - | ? | ? |
| DDB0234262 | 1246 | DDB_G0269948 | - | DPU_G0062624 | + | PPA_G1388862 | + | DFA_G1489414 | - | ? | ? |
| DDB0234303 | 1675 | DDB_G0269914 | - | DPU_G0060740 | + | PPA_G1302068 | + | DFA_G1580408 | - | ? | ? |
| DDB0235141 | 465 | DDB_G0274331 | - | DPU_G0073080 | + | PPA_G1417280 | - | DFA_G1472580 | + | EHI_056390 | - |
| DDB0235141 | 584 | DDB_G0274331 | - | DPU_G0073080 | + | PPA_G1417280 | + | DFA_G1472580 | + | EHI_056390 | - |
| DDB0235161 | 3254 | DDB_G0291093 | - | DPU_G0061222 | + | PPA_G1379698 | + | DFA_G1442176 | - | ? | ? |
| DDB0235223 | 988 | DDB_G0286111 | - | DPU_G0058886 | + | PPA_G1380724 | - | DFA_G1474578 | + | EHI_031230 | - |
| DDB0235226 | 694 | DDB_G0285461 | - | DPU_G0057352 | + | PPA_G1298424 | + | DFA_G1444092 | + | EHI_092280 | - |
| DDB0235237 | 3406 | DDB_G0269502 | - | DPU_G0072490 | + | PPA_G1286688 | + | DFA_G1472760 | + | ? | ? |
| DDB0235243 | 2163 | DDB_G0291163 | - | DPU_G0061196 | + | PPA_G1325450 | + | DFA_G1518790 | + | ? | ? |
| DDB0235245 | 802 | DDB_G0277955 | - | DPU_G0055614 | + | PPA_G1277770 | + | DFA_G1576526 | + | ? | ? |
| DDB0235281 | 1444 | DDB_G0292746 | - | DPU_G0055292 | + | PPA_G1394688 | - | DFA_G1499538 | + | EHI_073330 | - |
| DDB0235299 | 280 | DDB_G0283663 | - | DPU_G0058652 | + | PPA_G1400198 | + | ? | ? | ? | ? |
| DDB0235319 | 552 | DDB_G0289735 | - | DPU_G0055108 | + | ? | ? | DFA_G1583014 | + | ? | ? |
| DDB0235386 | 1194 | DDB_G0278063 | - | DPU_G0072346 | + | PPA_G1358142 | + | DFA_G1436000 | - | ? | ? |
| DDB0235386 | 1381 | DDB_G0278063 | - | DPU_G0072346 | + | PPA_G1358142 | ? | DFA_G1436000 | + | ? | ? |
| DDB0235401 | 1431 | DDB_G0269896 | - | DPU_G0057420 | + | PPA_G1314882 | + | DFA_G1471542 | + | ? | ? |
| DDB0235401 | 1785 | DDB_G0269896 | - | DPU_G0057420 | + | PPA_G1314882 | + | DFA_G1471542 | + | ? | ? |
| DDB0235401 | 3187 | DDB_G0269896 | - | DPU_G0057420 | + | PPA_G1314882 | + | DFA_G1471542 | ? | ? | ? |
| DDB0237493 | 2834 | DDB_G0279371 | - | DPU_G0067380 | + | PPA_G1383052 | + | ? | ? | EHI_138370 | - |
| DDB0237517 | 1993 | DDB_G0269694 | - | DPU_G0059928 | + | PPA_G1306392 | - | DFA_G1495300 | + | ? | ? |
| DDB0237525 | 1819 | DDB_G0271398 | - | DPU_G0064240 | + | PPA_G1337058 | - | DFA_G1511630 | + | ? | ? |
| DDB0237525 | 5138 | DDB_G0271398 | - | DPU_G0064240 | + | PPA_G1337058 | + | DFA_G1511630 | + | ? | ? |
| DDB0237531 | 932 | DDB_G0291832 | - | DPU_G0053992 | + | PPA_G1388628 | + | DFA_G1529786 | + | ? | ? |
| DDB0237549 | 1614 | DDB_G0267748 | - | DPU_G0058862 | + | PPA_G1347624 | + | DFA_G1557446 | + | EHI_049900 | - |
| DDB0237556 | 3087 | DDB_G0274785 | - | DPU_G0065512 | + | PPA_G1401514 | + | DFA_G1458132 | + | ? | ? |
| DDB0237583 | 1744 | DDB_G0284379 | - | DPU_G0052698 | + | PPA_G1355278 | + | DFA_G1559938 | + | ? | ? |
| DDB0237585 | 2549 | DDB_G0283119 | - | DPU_G0059184 | + | PPA_G1393662 | + | DFA_G1575166 | - | ? | ? |
| DDB0237606 | 1133 | DDB_G0271310 | - | DPU_G0074382 | + | PPA_G1431982 | + | DFA_G1486714 | - | ? | ? |
| DDB0237634 | 1200 | DDB_G0289461 | - | DPU_G0064612 | + | ? | ? | DFA_G1564918 | + | ? | ? |
| DDB0237644 | 1278 | DDB_G0268810 | - | DPU_G0053864 | + | PPA_G1305894 | + | DFA_G1457358 | - | ? | ? |
| DDB0237697 | 1257 | DDB_G0284381 | - | DPU_G0052700 | + | PPA_G1409604 | + | DFA_G1547164 | + | ? | ? |
| DDB0237710 | 563 | DDB_G0288647 | - | DPU_G0061856 | + | PPA_G1344930 | + | DFA_G1577854 | + | ? | ? |
| DDB0237713 | 810 | DDB_G0274585 | - | DPU_G0074492 | + | PPA_G1354834 | + | DFA_G1444612 | + | ? | ? |
| DDB0237719 | 125 | DDB_G0267594 | - | DPU_G0053546 | + | PPA_G1269514 | + | DFA_G1536654 | + | EHI_113900 | - |
| DDB0237749 | 1691 | DDB_G0285445 | - | DPU_G0060232 | + | PPA_G1411208 | + | ? | ? | ? | ? |
| DDB0237791 | 615 | DDB_G0276359 | - | DPU_G0070574 | + | PPA_G1400898 | + | DFA_G1562616 | + | EHI_197020 | - |
| DDB0237852 | 2753 | DDB_G0281163 | - | DPU_G0062902 | + | PPA_G1347214 | + | DFA_G1589938 | - | ? | ? |
| DDB0238039 | 340 | DDB_G0282195 | - | DPU_G0067492 | + | ? | ? | DFA_G1552370 | + | ? | ? |
| DDB0238043 | 462 | DDB_G0281589 | - | DPU_G0068316 | + | PPA_G1353592 | + | DFA_G1523610 | + | ? | ? |
| DDB0238056 | 1246 | DDB_G0284223 | - | DPU_G0068268 | + | PPA_G1409670 | + | DFA_G1476694 | - | ? | ? |
| DDB0238079 | 1216 | DDB_G0277113 | - | DPU_G0073476 | + | PPA_G1409506 | + | ? | ? | ? | ? |
| DDB0238083 | 741 | DDB_G0285143 | - | DPU_G0073606 | + | PPA_G1275968 | - | DFA_G1451126 | + | ? | ? |
| DDB0238106 | 301 | DDB_G0272668 | - | DPU_G0071794 | + | PPA_G1281454 | + | ? | ? | ? | ? |
| DDB0238180 | 1342 | DDB_G0269686 | - | DPU_G0070242 | + | PPA_G1383498 | + | DFA_G1491312 | - | ? | ? |
| DDB0238316 | 2223 | DDB_G0291396 | - | DPU_G0058692 | + | PPA_G1366752 | + | DFA_G1446718 | + | ? | ? |
| DDB0238322 | 252 | DDB_G0290083 | - | DPU_G0075880 | + | ? | ? | DFA_G1524472 | + | ? | ? |
| DDB0238370 | 1191 | DDB_G0270906 | - | DPU_G0070272 | + | PPA_G1428018 | + | ? | ? | ? | ? |
| DDB0238673 | 740 | DDB_G0270776 | - | DPU_G0060056 | + | PPA_G1400586 | + | DFA_G1478504 | + | ? | ? |
| DDB0238768 | 471 | DDB_G0291201 | - | DPU_G0062390 | + | PPA_G1322332 | + | DFA_G1527528 | - | ? | ? |
| DDB0238831 | 894 | DDB_G0273189 | - | DPU_G0056094 | + | PPA_G1297694 | - | DFA_G1571122 | + | ? | ? |
| DDB0238859 | 1232 | DDB_G0295687 | - | DPU_G0060904 | + | PPA_G1403948 | + | DFA_G1471816 | + | ? | ? |
| DDB0252606 | 493 | DDB_G0295781 | - | DPU_G0072086 | + | PPA_G1344946 | - | DFA_G1503108 | + | ? | ? |
| DDB0252812 | 1842 | DDB_G0295717 | - | DPU_G0058590 | + | PPA_G1343632 | + | DFA_G1558058 | + | ? | ? |
| DDB0252861 | 4488 | DDB_G0286969 | - | DPU_G0070784 | + | PPA_G1314544 | + | DFA_G1466302 | + | ? | ? |
| DDB0266346 | 110 | DDB_G0287203 | - | DPU_G0074892 | + | ? | ? | DFA_G1537400 | + | ? | ? |
| DDB0266387 | 81 | DDB_G0282887 | - | DPU_G0062834 | + | PPA_G1274540 | + | ? | ? | EHI_122820 | - |
| DDB0266389 | 1697 | DDB_G0289013 | - | DPU_G0064396 | + | PPA_G1311050 | + | DFA_G1581642 | - | ? | ? |
| DDB0266392 | 276 | DDB_G0292868 | - | DPU_G0075216 | + | PPA_G1340760 | + | DFA_G1565210 | + | EHI_177450 | - |
| DDB0266417 | 146 | DDB_G0288729 | - | DPU_G0068606 | + | PPA_G1305210 | - | DFA_G1544434 | + | ? | ? |
| DDB0266452 | 409 | DDB_G0292074 | - | DPU_G0052072 | + | PPA_G1420422 | + | ? | ? | ? | ? |
| DDB0266496 | 1266 | DDB_G0289423 | - | DPU_G0060402 | + | PPA_G1379410 | + | ? | ? | ? | ? |
| DDB0266619 | 901 | DDB_G0295827 | - | DPU_G0051126 | + | PPA_G1392544 | + | ? | ? | ? | ? |
| DDB0266633 | 436 | DDB_G0271706 | - | DPU_G0057858 | + | PPA_G1278450 | + | DFA_G1442010 | - | ? | ? |
| DDB0266676 | 208 | DDB_G0283683 | - | DPU_G0052810 | + | PPA_G1317692 | + | ? | ? | ? | ? |
| DDB0266726 | 264 | DDB_G0291910 | - | DPU_G0063938 | + | PPA_G1314596 | + | DFA_G1501282 | - | ? | ? |
| DDB0266744 | 2251 | DDB_G0278991 | - | DPU_G0072360 | + | PPA_G1282064 | - | DFA_G1536094 | + | ? | ? |
| DDB0266862 | 705 | DDB_G0279967 | - | DPU_G0066320 | + | PPA_G1325282 | + | ? | ? | ? | ? |
| DDB0266864 | 938 | DDB_G0281815 | - | DPU_G0066434 | + | PPA_G1424356 | + | DFA_G1495868 | + | ? | ? |
| DDB0266931 | 1167 | DDB_G0275967 | - | DPU_G0067800 | + | PPA_G1295870 | + | DFA_G1554206 | + | ? | ? |
| DDB0267019 | 369 | DDB_G0278849 | - | DPU_G0074562 | + | PPA_G1313900 | + | DFA_G1564098 | + | ? | ? |
| DDB0267019 | 789 | DDB_G0278849 | - | DPU_G0074562 | + | PPA_G1313900 | + | DFA_G1564098 | - | ? | ? |
| DDB0267034 | 270 | DDB_G0275265 | - | DPU_G0074358 | + | PPA_G1417982 | + | DFA_G1543876 | - | ? | ? |
| DDB0267054 | 306 | DDB_G0291814 | - | DPU_G0068538 | + | PPA_G1382230 | - | DFA_G1587306 | + | ? | ? |
| DDB0267059 | 108 | DDB_G0281937 | - | DPU_G0060704 | + | PPA_G1328888 | + | DFA_G1467130 | - | ? | ? |
| DDB0302367 | 2588 | DDB_G0292252 | - | DPU_G0067556 | + | PPA_G1397688 | + | DFA_G1594846 | + | ? | ? |
| DDB0302378 | 910 | DDB_G0283281 | - | DPU_G0052878 | + | PPA_G1283934 | + | DFA_G1596578 | + | ? | ? |
| DDB0302412 | 140 | DDB_G0278983 | - | DPU_G0054784 | + | PPA_G1326662 | + | DFA_G1544766 | + | EHI_184000 | - |
| DDB0302463 | 548 | DDB_G0275371 | - | DPU_G0057788 | + | PPA_G1287680 | + | ? | ? | ? | ? |
| DDB0302480 | 187 | DDB_G0272710 | - | DPU_G0053420 | + | PPA_G1285060 | + | DFA_G1469144 | + | ? | ? |
| DDB0302532 | 759 | DDB_G0277951 | - | DPU_G0055618 | + | PPA_G1329650 | - | DFA_G1576656 | + | ? | ? |
| DDB0304380 | 493 | DDB_G0288287 | - | DPU_G0053086 | + | PPA_G1423654 | + | DFA_G1576186 | - | ? | ? |
| DDB0304420 | 231 | DDB_G0279375 | - | DPU_G0067384 | + | PPA_G1349156 | - | DFA_G1557654 | + | ? | ? |
| DDB0304559 | 631 | DDB_G0278887 | - | DPU_G0052372 | + | PPA_G1420916 | + | DFA_G1589672 | + | ? | ? |
| DDB0304576 | 651 | DDB_G0285927 | - | DPU_G0058830 | + | PPA_G1332290 | + | DFA_G1494694 | + | ? | ? |
| DDB0304679 | 2700 | DDB_G0292724 | - | DPU_G0054280 | + | PPA_G1281322 | + | DFA_G1463934 | - | ? | ? |
| DDB0304693 | 2280 | DDB_G0288383 | - | DPU_G0071828 | + | PPA_G1343604 | + | DFA_G1548980 | + | ? | ? |
| DDB0304712 | 806 | DDB_G0270196 | - | DPU_G0060804 | + | PPA_G1347258 | + | DFA_G1574240 | - | ? | ? |
| DDB0304822 | 692 | DDB_G0288225 | - | DPU_G0058492 | + | PPA_G1335574 | + | DFA_G1595608 | - | ? | ? |
| DDB0304825 | 179 | DDB_G0288271 | - | DPU_G0072174 | + | PPA_G1297384 | - | DFA_G1457640 | + | ? | ? |
| DDB0304989 | 460 | DDB_G0286521 | - | DPU_G0060474 | + | PPA_G1369970 | + | ? | ? | ? | ? |
| DDB0305011 | 370 | DDB_G0279265 | - | DPU_G0056972 | + | PPA_G1411400 | + | DFA_G1527798 | - | ? | ? |
| DDB0305032 | 695 | DDB_G0278713 | - | DPU_G0066008 | + | PPA_G1333204 | + | DFA_G1483240 | + | EHI_188790 | - |
| DDB0305243 | 138 | DDB_G0286691 | - | DPU_G0067696 | + | PPA_G1285050 | + | DFA_G1556570 | - | ? | ? |
| DDB0305316 | 151 | DDB_G0269722 | - | DPU_G0062636 | + | PPA_G1303952 | + | DFA_G1435792 | + | EHI_029570 | - |
| DDB0305330 | 473 | DDB_G0269890 | - | DPU_G0069636 | + | PPA_G1402836 | + | DFA_G1529992 | + | EHI_136490 | - |
| DDB0305348 | 1094 | DDB_G0292494 | - | DPU_G0057978 | + | PPA_G1373276 | + | DFA_G1491128 | + | ? | ? |
| DDB0305375 | 240 | DDB_G0278649 | - | DPU_G0059202 | + | PPA_G1408266 | - | DFA_G1486612 | + | EHI_050300 | - |
| DDB0305388 | 289 | DDB_G0271910 | - | DPU_G0066826 | + | ? | ? | DFA_G1488226 | + | EHI_016460 | - |
| DDB0305430 | 241 | DDB_G0274663 | - | DPU_G0073140 | + | ? | ? | DFA_G1461986 | + | ? | ? |
| DDB0305434 | 79 | DDB_G0274871 | - | DPU_G0069526 | + | PPA_G1386640 | + | DFA_G1518946 | + | ? | ? |
| DDB0305437 | 269 | DDB_G0279553 | - | DPU_G0058062 | + | ? | ? | DFA_G1526618 | + | ? | ? |
| DDB0305499 | 649 | DDB_G0277149 | - | DPU_G0059242 | + | PPA_G1319152 | - | DFA_G1526248 | + | ? | ? |
| DDB0305657 | 337 | DDB_G0267992 | - | DPU_G0074398 | + | PPA_G1357308 | + | DFA_G1465732 | + | ? | ? |
| DDB0305692 | 272 | DDB_G0278415 | - | DPU_G0057152 | + | PPA_G1366242 | + | ? | ? | ? | ? |
| DDB0305720 | 206 | DDB_G0279451 | - | DPU_G0054942 | + | PPA_G1357360 | + | DFA_G1543282 | + | ? | ? |
| DDB0305730 | 125 | DDB_G0280371 | - | DPU_G0054388 | + | PPA_G1374452 | + | DFA_G1507996 | + | EHI_164850 | - |
| DDB0305743 | 608 | DDB_G0278893 | - | DPU_G0071312 | + | PPA_G1273162 | - | DFA_G1532502 | + | ? | ? |
| DDB0305830 | 194 | DDB_G0290359 | - | DPU_G0067528 | + | PPA_G1307000 | + | DFA_G1489828 | + | ? | ? |
| DDB0306216 | 52 | DDB_G0278499 | - | DPU_G0051342 | + | ? | ? | DFA_G1579416 | + | ? | ? |
| DDB0306240 | 671 | DDB_G0270074 | - | DPU_G0060842 | + | PPA_G1388014 | + | DFA_G1543354 | - | ? | ? |
| DDB0306271 | 2133 | DDB_G0282491 | - | DPU_G0063326 | + | PPA_G1430504 | + | DFA_G1549992 | - | ? | ? |
| DDB0306355 | 2700 | DDB_G0283475 | - | DPU_G0066734 | + | PPA_G1279416 | + | DFA_G1553202 | - | ? | ? |
| DDB0306402 | 410 | DDB_G0287037 | - | DPU_G0053152 | + | PPA_G1362990 | + | DFA_G1593322 | + | ? | ? |
| DDB0306430 | 1161 | DDB_G0288069 | - | DPU_G0066162 | + | PPA_G1339420 | - | DFA_G1458536 | + | ? | ? |
| DDB0306442 | 1220 | DDB_G0285655 | - | DPU_G0054110 | + | PPA_G1313182 | + | DFA_G1473622 | + | ? | ? |
| DDB0306479 | 731 | DDB_G0288385 | - | DPU_G0071832 | + | PPA_G1306606 | + | DFA_G1595224 | + | ? | ? |
| DDB0306566 | 3502 | DDB_G0269288 | - | DPU_G0053772 | + | PPA_G1365672 | + | DFA_G1558604 | + | ? | ? |
| DDB0306577 | 294 | DDB_G0269588 | - | DPU_G0066566 | + | ? | ? | DFA_G1540098 | + | ? | ? |
| DDB0306585 | 627 | DDB_G0269734 | - | DPU_G0060564 | + | PPA_G1398474 | + | DFA_G1569592 | + | ? | ? |
| DDB0306604 | 502 | DDB_G0270018 | - | DPU_G0066590 | + | PPA_G1433452 | + | DFA_G1489756 | + | ? | ? |
| DDB0306615 | 1185 | DDB_G0270112 | - | DPU_G0074614 | + | PPA_G1346146 | + | DFA_G1446310 | + | ? | ? |
| DDB0306627 | 894 | DDB_G0275577 | - | DPU_G0070938 | + | PPA_G1274176 | + | DFA_G1487294 | - | ? | ? |
| DDB0306644 | 2485 | DDB_G0275889 | - | DPU_G0055070 | + | PPA_G1304784 | + | DFA_G1569830 | + | ? | ? |
| DDB0306674 | 1188 | DDB_G0289465 | - | DPU_G0062432 | + | PPA_G1313048 | + | DFA_G1592282 | + | ? | ? |
| DDB0306678 | 970 | DDB_G0289773 | - | DPU_G0061014 | + | ? | ? | DFA_G1560496 | + | ? | ? |
| DDB0306682 | 1353 | DDB_G0289953 | - | DPU_G0052360 | + | PPA_G1331802 | + | DFA_G1565476 | - | ? | ? |
| DDB0306732 | 831 | DDB_G0291554 | - | DPU_G0075086 | + | PPA_G1343954 | - | DFA_G1510210 | + | ? | ? |
| DDB0306734 | 1124 | DDB_G0291694 | - | DPU_G0055528 | + | PPA_G1429280 | + | DFA_G1570636 | - | ? | ? |
| DDB0306751 | 9070 | DDB_G0292230 | - | DPU_G0056210 | + | PPA_G1272422 | + | DFA_G1452686 | + | ? | ? |
| DDB0306752 | 2138 | DDB_G0292234 | - | DPU_G0064886 | + | PPA_G1399252 | - | DFA_G1488010 | + | ? | ? |
| DDB0306787 | 5409 | DDB_G0293090 | - | DPU_G0056010 | + | PPA_G1291992 | + | DFA_G1596838 | - | ? | ? |
| DDB0306812 | 169 | DDB_G0288523 | - | DPU_G0068058 | + | ? | ? | DFA_G1523440 | + | ? | ? |
| DDB0306814 | 4611 | DDB_G0289043 | - | DPU_G0056388 | + | PPA_G1273804 | + | DFA_G1475710 | + | ? | ? |
| DDB0306818 | 1181 | DDB_G0289769 | - | DPU_G0061018 | + | PPA_G1412894 | + | ? | ? | ? | ? |
| DDB0306835 | 7717 | DDB_G0292046 | - | DPU_G0054644 | + | PPA_G1312424 | + | DFA_G1451006 | + | ? | ? |
| DDB0306837 | 2058 | DDB_G0292290 | - | DPU_G0054038 | + | PPA_G1414766 | + | DFA_G1545958 | - | ? | ? |
| DDB0306842 | 1149 | DDB_G0293622 | - | DPU_G0052010 | + | PPA_G1276848 | + | DFA_G1551170 | + | EHI_119610 | - |
| DDB0306869 | 675 | DDB_G0276431 | - | DPU_G0058248 | + | PPA_G1273386 | + | DFA_G1496944 | - | ? | ? |
| DDB0306892 | 1090 | DDB_G0278923 | - | DPU_G0058114 | + | PPA_G1404208 | ? | DFA_G1538022 | + | EHI_049950 | - |
| DDB0306892 | 1416 | DDB_G0278923 | - | DPU_G0058114 | + | PPA_G1404208 | + | DFA_G1538022 | + | EHI_049950 | - |
| DDB0306892 | 1691 | DDB_G0278923 | - | DPU_G0058114 | + | PPA_G1404208 | + | DFA_G1538022 | + | EHI_049950 | - |
| DDB0306916 | 1368 | DDB_G0279435 | - | DPU_G0054138 | + | ? | ? | DFA_G1545854 | + | ? | ? |
| DDB0306929 | 674 | DDB_G0279089 | - | DPU_G0075666 | + | PPA_G1396670 | - | DFA_G1482726 | + | ? | ? |
| DDB0306935 | 347 | DDB_G0279647 | - | DPU_G0060520 | + | PPA_G1426774 | + | DFA_G1571990 | + | EHI_000740 | - |
| DDB0307007 | 2600 | DDB_G0281543 | - | DPU_G0074222 | + | PPA_G1381238 | + | DFA_G1580694 | + | ? | ? |
| DDB0307028 | 430 | DDB_G0284051 | - | DPU_G0072672 | + | PPA_G1381606 | - | DFA_G1533890 | + | ? | ? |
| DDB0307028 | 946 | DDB_G0284051 | - | DPU_G0072672 | + | PPA_G1381606 | + | DFA_G1533890 | + | ? | ? |
| DDB0307110 | 4236 | DDB_G0270432 | - | DPU_G0066046 | + | ? | ? | DFA_G1499706 | + | ? | ? |
| DDB0307110 | 4379 | DDB_G0270432 | - | DPU_G0066046 | + | ? | ? | DFA_G1499706 | + | ? | ? |
| DDB0307139 | 2191 | DDB_G0272000 | - | DPU_G0053932 | + | ? | ? | DFA_G1501514 | + | ? | ? |
| DDB0307164 | 1730 | DDB_G0272470 | - | DPU_G0064320 | + | PPA_G1413426 | + | DFA_G1501242 | + | ? | ? |
| DDB0307168 | 3153 | DDB_G0272502 | - | DPU_G0057654 | + | ? | ? | DFA_G1483936 | + | ? | ? |
| DDB0307251 | 614 | DDB_G0275107 | - | DPU_G0071874 | + | PPA_G1382782 | + | DFA_G1496872 | + | ? | ? |
| DDB0307362 | 2102 | DDB_G0288963 | - | DPU_G0058722 | + | PPA_G1415588 | - | DFA_G1477660 | + | ? | ? |
| DDB0307378 | 3752 | DDB_G0267840 | - | DPU_G0061386 | + | PPA_G1283994 | + | DFA_G1546966 | - | ? | ? |
| DDB0307378 | 4320 | DDB_G0267840 | - | DPU_G0061386 | + | PPA_G1283994 | ? | DFA_G1546966 | + | ? | ? |
| DDB0307393 | 1095 | DDB_G0268230 | - | DPU_G0068630 | + | PPA_G1380652 | + | DFA_G1551402 | - | ? | ? |
| DDB0307409 | 441 | DDB_G0269060 | - | DPU_G0066350 | + | PPA_G1434508 | + | DFA_G1539524 | - | ? | ? |
| DDB0307415 | 506 | DDB_G0269358 | - | DPU_G0074996 | + | PPA_G1303196 | + | ? | ? | ? | ? |
| DDB0307429 | 2178 | DDB_G0269700 | - | DPU_G0059934 | + | PPA_G1414946 | + | DFA_G1495138 | + | ? | ? |
| DDB0307464 | 719 | DDB_G0270278 | - | DPU_G0060330 | + | ? | ? | DFA_G1494528 | + | ? | ? |
| DDB0307502 | 2964 | DDB_G0272206 | - | DPU_G0068754 | + | PPA_G1295412 | - | DFA_G1478348 | + | ? | ? |
| DDB0307569 | 6527 | DDB_G0275287 | - | DPU_G0070934 | + | PPA_G1407640 | + | DFA_G1578182 | + | ? | ? |
| DDB0307642 | 2370 | DDB_G0278229 | - | DPU_G0054016 | + | PPA_G1416520 | - | DFA_G1564700 | + | ? | ? |
| DDB0307748 | 1885 | DDB_G0283403 | - | DPU_G0068516 | + | PPA_G1332834 | + | DFA_G1443336 | + | ? | ? |
| DDB0307803 | 2187 | DDB_G0284569 | - | DPU_G0071918 | + | PPA_G1273036 | + | ? | ? | ? | ? |
| DDB0307839 | 670 | DDB_G0285437 | - | DPU_G0066674 | + | PPA_G1293540 | - | DFA_G1558938 | + | ? | ? |
| DDB0307890 | 2449 | DDB_G0278301 | - | DPU_G0060196 | + | PPA_G1363310 | + | DFA_G1505578 | + | ? | ? |
| DDB0307902 | 535 | DDB_G0277763 | - | DPU_G0066258 | + | ? | ? | DFA_G1527184 | + | ? | ? |
| DDB0307907 | 273 | DDB_G0277757 | - | DPU_G0059732 | + | PPA_G1278634 | + | DFA_G1442108 | - | ? | ? |
| DDB0307968 | 2142 | DDB_G0278757 | - | DPU_G0051350 | + | PPA_G1299250 | + | DFA_G1449698 | + | ? | ? |
| DDB0307981 | 2345 | DDB_G0279105 | - | DPU_G0057034 | + | PPA_G1296696 | + | ? | ? | ? | ? |
| DDB0308127 | 855 | DDB_G0289829 | - | DPU_G0060974 | + | PPA_G1281720 | + | DFA_G1544940 | - | ? | ? |
| DDB0308228 | 413 | DDB_G0272214 | - | DPU_G0066948 | + | PPA_G1404956 | + | DFA_G1499516 | + | ? | ? |
| DDB0308238 | 1250 | DDB_G0278671 | - | DPU_G0067182 | + | ? | ? | DFA_G1474426 | + | ? | ? |
| DDB0308256 | 1489 | DDB_G0275113 | - | DPU_G0058950 | + | ? | ? | DFA_G1507692 | + | ? | ? |
| DDB0308281 | 611 | DDB_G0281705 | - | DPU_G0066388 | + | PPA_G1423462 | + | DFA_G1464932 | - | ? | ? |
| DDB0308301 | 428 | DDB_G0293658 | - | DPU_G0072944 | + | PPA_G1403768 | + | DFA_G1577804 | - | ? | ? |
| DDB0308455 | 389 | DDB_G0272971 | - | DPU_G0053438 | + | PPA_G1432098 | + | DFA_G1523700 | + | ? | ? |
| DDB0308500 | 842 | DDB_G0273617 | - | DPU_G0068762 | + | PPA_G1330290 | + | DFA_G1542892 | + | ? | ? |
| DDB0308562 | 2004 | DDB_G0273869 | - | DPU_G0065960 | + | PPA_G1426856 | + | DFA_G1559554 | - | ? | ? |
| DDB0308629 | 218 | DDB_G0275089 | - | DPU_G0063700 | + | ? | ? | DFA_G1522304 | + | ? | ? |
| DDB0308655 | 2347 | DDB_G0276089 | - | DPU_G0060374 | + | PPA_G1273880 | + | DFA_G1548374 | + | ? | ? |
| DDB0308657 | 403 | DDB_G0276117 | - | DPU_G0059408 | + | PPA_G1376944 | + | ? | ? | ? | ? |
| DDB0309079 | 106 | DDB_G0285831 | - | DPU_G0067450 | + | PPA_G1358212 | + | DFA_G1466968 | + | ? | ? |
| DDB0346518 | 446 | DDB_G0289955 | - | DPU_G0052362 | + | PPA_G1331814 | + | DFA_G1565510 | + | ? | ? |
| DDB0346706 | 1793 | DDB_G0268424 | - | DPU_G0062080 | + | ? | ? | DFA_G1488466 | + | ? | ? |
| DDB0346717 | 1509 | DDB_G0293868 | - | DPU_G0073778 | + | PPA_G1300984 | + | DFA_G1476260 | - | ? | ? |
| DDB0346777 | 1086 | DDB_G0270722 | - | DPU_G0053368 | + | PPA_G1292994 | + | ? | ? | ? | ? |
| DDB0346779 | 4735 | DDB_G0270774 | - | DPU_G0060076 | + | PPA_G1359124 | - | DFA_G1548800 | + | ? | ? |
| DDB0346896 | 715 | DDB_G0274953 | - | DPU_G0074512 | + | PPA_G1330012 | + | DFA_G1465880 | + | ? | ? |
| DDB0346987 | 1660 | DDB_G0279429 | - | DPU_G0060894 | + | PPA_G1281584 | + | DFA_G1558790 | - | ? | ? |
| DDB0347093 | 2759 | DDB_G0292286 | - | DPU_G0067052 | + | PPA_G1382696 | + | DFA_G1473390 | + | ? | ? |
| DDB0347132 | 880 | DDB_G0289019 | - | DPU_G0058716 | + | PPA_G1309202 | + | DFA_G1554542 | + | ? | ? |
| DDB0347369 | 721 | DDB_G0291468 | - | DPU_G0073988 | + | PPA_G1385846 | + | DFA_G1550594 | - | EHI_178090 | - |
| DDB0347369 | 822 | DDB_G0291468 | - | DPU_G0073988 | + | PPA_G1385846 | + | DFA_G1550594 | + | EHI_178090 | - |
| DDB0347474 | 211 | DDB_G0288243 | - | DPU_G0058494 | + | ? | ? | DFA_G1587370 | + | ? | ? |
| DDB0347626 | 1851 | DDB_G0280773 | - | DPU_G0070916 | + | PPA_G1331904 | + | DFA_G1453390 | - | ? | ? |
| DDB0347708 | 652 | DDB_G0291632 | - | DPU_G0075136 | + | ? | ? | DFA_G1540218 | + | ? | ? |
| DDB0347710 | 926 | DDB_G0285991 | - | DPU_G0058842 | + | ? | ? | DFA_G1498614 | + | ? | ? |
| DDB0347893 | 139 | DDB_G0276347 | - | DPU_G0055910 | + | PPA_G1277274 | + | DFA_G1482980 | + | ? | ? |
| DDB0347971 | 2701 | DDB_G0269710 | - | DPU_G0062228 | + | PPA_G1421260 | + | DFA_G1597754 | - | ? | ? |
| DDB0348060 | 1848 | DDB_G0279583 | - | DPU_G0054292 | + | PPA_G1379244 | + | DFA_G1525660 | + | ? | ? |
| DDB0348060 | 2202 | DDB_G0279583 | - | DPU_G0054292 | + | PPA_G1379244 | + | DFA_G1525660 | + | ? | ? |
| DDB0348248 | 4534 | DDB_G0291263 | - | DPU_G0075130 | + | PPA_G1429460 | + | DFA_G1570610 | + | ? | ? |
| DDB0348361 | 808 | DDB_G0278675 | - | DPU_G0054024 | + | PPA_G1391024 | + | DFA_G1519948 | - | ? | ? |
| DDB0348485 | 2016 | DDB_G0276325 | - | DPU_G0055762 | + | PPA_G1293274 | + | DFA_G1544580 | + | ? | ? |
| DDB0348819 | 704 | DDB_G0267658 | - | DPU_G0070514 | + | PPA_G1361424 | - | DFA_G1550956 | + | ? | ? |
| DDB0349186 | 1138 | DDB_G0278629 | - | DPU_G0055608 | + | PPA_G1390608 | + | DFA_G1504760 | - | ? | ? |
| DDB0349233 | 2533 | DDB_G0278143 | - | DPU_G0068282 | + | PPA_G1290760 | - | DFA_G1519258 | + | ? | ? |
| DDB0349235 | 2821 | DDB_G0282421 | - | DPU_G0054550 | + | PPA_G1392974 | + | ? | ? | ? | ? |
| DDB0349652 | 460 | DDB_G0273135 | - | DPU_G0063598 | + | PPA_G1424106 | + | ? | ? | ? | ? |
| DDB0350236 | 589 | DDB_G0350235 | - | DPU_G0075618 | + | PPA_G1359094 | + | ? | ? | ? | ? |
| **The imprecise intron losses in *D. discoideum*** | | | | | | | | | | | |
| DDB0201559 | 1474 | DDB_G0290963 | - | DPU_G0061224 | + | PPA_G1287054 | + | DFA_G1438408 | + | ? | ? |
| DDB0220499 | 2396 | DDB_G0280995 | - | DPU_G0057164 | + | PPA_G1331882 | + | DFA_G1453182 | + | ? | ? |
| **The precise intron losses in *D. purpureum*** | | | | | | | | | | | |
| DPU0051389 | 1350 | DDB_G0279245 | + | DPU_G0051388 | - | PPA_G1416710 | + | DFA_G1564882 | - | ? | ? |
| DPU0051415 | 328 | DDB_G0287517 | + | DPU_G0051414 | - | PPA_G1280718 | + | DFA_G1594046 | + | ? | ? |
| DPU0051467 | 2113 | DDB_G0288121 | + | DPU_G0051466 | - | PPA_G1279242 | + | DFA_G1506418 | - | ? | ? |
| DPU0051537 | 3019 | DDB_G0286665 | + | DPU_G0051536 | - | PPA_G1303520 | + | DFA_G1523932 | - | ? | ? |
| DPU0051877 | 262 | DDB_G0291360 | + | DPU_G0051876 | - | ? | ? | DFA_G1538344 | + | ? | ? |
| DPU0051879 | 417 | DDB_G0291362 | + | DPU_G0051878 | - | PPA_G1434128 | + | DFA_G1588800 | + | ? | ? |
| DPU0051885 | 178 | DDB_G0291894 | + | DPU_G0051884 | - | PPA_G1293394 | + | DFA_G1518646 | + | ? | ? |
| DPU0052169 | 3369 | DDB_G0281585 | + | DPU_G0052168 | - | PPA_G1331376 | + | DFA_G1580934 | - | ? | ? |
| DPU0052571 | 722 | DDB_G0271832 | + | DPU_G0052570 | - | PPA_G1333410 | + | ? | ? | ? | ? |
| DPU0052685 | 788 | DDB_G0284359 | + | DPU_G0052684 | - | PPA_G1293080 | + | DFA_G1470376 | + | ? | ? |
| DPU0052733 | 254 | DDB_G0284195 | + | DPU_G0052732 | - | ? | ? | DFA_G1585628 | + | ? | ? |
| DPU0053019 | 2572 | DDB_G0276229 | + | DPU_G0053018 | - | PPA_G1413722 | - | DFA_G1495224 | + | ? | ? |
| DPU0053103 | 1394 | DDB_G0288267 | + | DPU_G0053102 | - | PPA_G1357486 | - | DFA_G1580314 | + | EHI_103730 | - |
| DPU0053239 | 963 | DDB_G0282271 | + | DPU_G0053238 | - | PPA_G1328050 | + | DFA_G1495614 | + | EHI_131540 | - |
| DPU0053339 | 113 | DDB_G0290483 | + | DPU_G0053338 | - | PPA_G1279598 | + | DFA_G1493586 | - | EHI_156200 | - |
| DPU0053369 | 1116 | DDB_G0270722 | + | DPU_G0053368 | - | PPA_G1292994 | + | ? | ? | ? | ? |
| DPU0053641 | 654 | DDB_G0284735 | + | DPU_G0053640 | - | PPA_G1416386 | + | DFA_G1471904 | + | EHI_088220 | - |
| DPU0053865 | 1052 | DDB_G0268810 | + | DPU_G0053864 | - | PPA_G1305894 | + | DFA_G1457358 | + | ? | ? |
| DPU0053987 | 1157 | DDB_G0291344 | + | DPU_G0053986 | - | PPA_G1380028 | - | DFA_G1580906 | + | ? | ? |
| DPU0054055 | 1145 | DDB_G0278781 | + | DPU_G0054054 | - | PPA_G1348992 | + | DFA_G1503698 | + | ? | ? |
| DPU0054061 | 564 | DDB_G0278779 | + | DPU_G0054060 | - | PPA_G1373468 | - | DFA_G1548462 | + | ? | ? |
| DPU0054577 | 649 | DDB_G0280975 | + | DPU_G0054576 | - | PPA_G1270660 | - | DFA_G1507612 | + | ? | ? |
| DPU0054621 | 751 | DDB_G0280105 | + | DPU_G0054620 | - | PPA_G1409328 | - | DFA_G1529722 | + | ? | ? |
| DPU0054763 | 414 | DDB_G0279159 | + | DPU_G0054762 | - | PPA_G1354164 | + | DFA_G1441024 | + | ? | ? |
| DPU0054777 | 115 | DDB_G0279557 | + | DPU_G0054776 | - | PPA_G1368442 | + | ? | ? | ? | ? |
| DPU0054791 | 302 | DDB_G0292906 | + | DPU_G0054790 | - | PPA_G1289928 | + | DFA_G1486544 | + | ? | ? |
| DPU0054909 | 4719 | DDB_G0270404 | + | DPU_G0054908 | - | PPA_G1281762 | + | DFA_G1527488 | - | ? | ? |
| DPU0054917 | 1527 | DDB_G0270418 | + | DPU_G0054916 | - | PPA_G1405132 | - | DFA_G1513446 | + | EHI_120640 | - |
| DPU0054929 | 311 | DDB_G0278925 | + | DPU_G0054928 | - | PPA_G1307932 | + | DFA_G1478860 | + | ? | ? |
| DPU0054943 | 487 | DDB_G0279451 | + | DPU_G0054942 | - | PPA_G1357360 | + | DFA_G1543282 | - | ? | ? |
| DPU0055011 | 285 | DDB_G0283823 | + | DPU_G0055010 | - | PPA_G1332760 | + | DFA_G1545694 | - | ? | ? |
| DPU0055019 | 4484 | DDB_G0283883 | + | DPU_G0055018 | - | PPA_G1332968 | - | DFA_G1519790 | + | ? | ? |
| DPU0055021 | 121 | DDB_G0268092 | + | DPU_G0055020 | - | PPA_G1283462 | + | ? | ? | ? | ? |
| DPU0055131 | 437 | DDB_G0281337 | + | DPU_G0055130 | - | PPA_G1299930 | - | DFA_G1479652 | + | ? | ? |
| DPU0055153 | 2279 | DDB_G0293538 | + | DPU_G0055152 | - | PPA_G1411346 | + | ? | ? | ? | ? |
| DPU0055317 | 425 | DDB_G0293298 | + | DPU_G0055316 | - | PPA_G1388424 | + | DFA_G1463062 | + | EHI_013760 | - |
| DPU0055351 | 561 | DDB_G0293978 | + | DPU_G0055350 | - | PPA_G1325854 | + | DFA_G1558388 | + | ? | ? |
| DPU0055427 | 499 | DDB_G0283773 | + | DPU_G0055426 | - | PPA_G1278828 | + | DFA_G1441912 | + | ? | ? |
| DPU0055451 | 206 | DDB_G0284037 | + | DPU_G0055450 | - | PPA_G1325050 | + | DFA_G1496024 | + | ? | ? |
| DPU0055461 | 267 | DDB_G0274687 | + | DPU_G0055460 | - | ? | ? | DFA_G1518930 | + | ? | ? |
| DPU0055513 | 339 | DDB_G0271572 | + | DPU_G0055512 | - | PPA_G1413176 | + | DFA_G1543936 | + | EHI_021400 | - |
| DPU0055619 | 466 | DDB_G0277951 | + | DPU_G0055618 | - | PPA_G1329650 | + | ? | ? | ? | ? |
| DPU0055901 | 2793 | DDB_G0277019 | + | DPU_G0055900 | - | PPA_G1336118 | + | DFA_G1584996 | - | ? | ? |
| DPU0056007 | 454 | DDB_G0293086 | + | DPU_G0056006 | - | PPA_G1300670 | + | DFA_G1493998 | + | ? | ? |
| DPU0056169 | 217 | DDB_G0275655 | + | DPU_G0056168 | - | PPA_G1307086 | + | DFA_G1510550 | - | ? | ? |
| DPU0056585 | 1395 | DDB_G0285683 | + | DPU_G0056584 | - | PPA_G1374668 | - | DFA_G1487540 | + | EHI_118720 | - |
| DPU0056585 | 1652 | DDB_G0285683 | + | DPU_G0056584 | - | PPA_G1374668 | - | DFA_G1487540 | + | EHI_118720 | - |
| DPU0056737 | 413 | DDB_G0284489 | + | DPU_G0056736 | - | ? | ? | DFA_G1469888 | + | ? | ? |
| DPU0057345 | 1047 | DDB_G0284833 | + | DPU_G0057344 | - | PPA_G1349572 | - | DFA_G1530936 | + | ? | ? |
| DPU0057345 | 491 | DDB_G0284833 | + | DPU_G0057344 | - | PPA_G1349572 | + | DFA_G1530936 | + | ? | ? |
| DPU0057569 | 2026 | DDB_G0278105 | + | DPU_G0057568 | - | PPA_G1330506 | - | DFA_G1471164 | + | ? | ? |
| DPU0057739 | 370 | DDB_G0276673 | + | DPU_G0057738 | - | PPA_G1309678 | + | DFA_G1465830 | + | ? | ? |
| DPU0057753 | 824 | DDB_G0277013 | + | DPU_G0057752 | - | ? | ? | DFA_G1593284 | + | ? | ? |
| DPU0057995 | 819 | DDB_G0292678 | + | DPU_G0057994 | - | PPA_G1281180 | + | DFA_G1553596 | - | ? | ? |
| DPU0058193 | 65 | DDB_G0272208 | + | DPU_G0058192 | - | PPA_G1322270 | + | ? | ? | ? | ? |
| DPU0058263 | 167 | DDB_G0291976 | + | DPU_G0058262 | - | PPA_G1392726 | + | DFA_G1468898 | - | ? | ? |
| DPU0058293 | 211 | DDB_G0267670 | + | DPU_G0058292 | - | PPA_G1314586 | - | DFA_G1482380 | + | ? | ? |
| DPU0058441 | 411 | DDB_G0282427 | + | DPU_G0058440 | - | PPA_G1360616 | + | DFA_G1465342 | + | ? | ? |
| DPU0058467 | 1199 | DDB_G0287711 | + | DPU_G0058466 | - | ? | ? | DFA_G1446942 | + | ? | ? |
| DPU0058653 | 572 | DDB_G0283663 | + | DPU_G0058652 | - | PPA_G1400198 | + | ? | ? | ? | ? |
| DPU0058673 | 886 | DDB_G0267872 | + | DPU_G0058672 | - | PPA_G1370350 | + | DFA_G1527250 | + | ? | ? |
| DPU0059151 | 1260 | DDB_G0284473 | + | DPU_G0059150 | - | PPA_G1412362 | - | DFA_G1548146 | + | ? | ? |
| DPU0059417 | 56 | DDB_G0279941 | + | DPU_G0059416 | - | PPA_G1339476 | + | DFA_G1593580 | + | ? | ? |
| DPU0059575 | 3210 | DDB_G0280525 | + | DPU_G0059574 | - | PPA_G1412044 | - | DFA_G1447950 | + | ? | ? |
| DPU0059713 | 439 | DDB_G0286455 | + | DPU_G0059712 | - | PPA_G1422824 | + | ? | ? | ? | ? |
| DPU0059719 | 472 | DDB_G0276763 | + | DPU_G0059718 | - | PPA_G1332542 | + | DFA_G1478422 | + | ? | ? |
| DPU0059861 | 1091 | DDB_G0271550 | + | DPU_G0059860 | - | PPA_G1326014 | + | DFA_G1451640 | - | ? | ? |
| DPU0059905 | 545 | DDB_G0275925 | + | DPU_G0059904 | - | PPA_G1271818 | + | DFA_G1592018 | - | EHI_198950 | - |
| DPU0059913 | 806 | DDB_G0275711 | + | DPU_G0059912 | - | PPA_G1330862 | + | DFA_G1451822 | - | ? | ? |
| DPU0059957 | 279 | DDB_G0289455 | + | DPU_G0059956 | - | PPA_G1283056 | + | DFA_G1587274 | + | ? | ? |
| DPU0060041 | 851 | DDB_G0269864 | + | DPU_G0060040 | - | PPA_G1349814 | + | DFA_G1444764 | + | ? | ? |
| DPU0060101 | 2138 | DDB_G0270040 | + | DPU_G0060100 | - | PPA_G1318586 | + | ? | ? | ? | ? |
| DPU0060243 | 675 | DDB_G0270866 | + | DPU_G0060242 | - | PPA_G1315016 | + | DFA_G1471680 | + | ? | ? |
| DPU0060351 | 348 | DDB_G0282921 | + | DPU_G0060350 | - | PPA_G1267936 | - | DFA_G1550066 | + | EHI_108760 | - |
| DPU0060429 | 7719 | DDB_G0288041 | + | DPU_G0060428 | - | PPA_G1360558 | - | DFA_G1556324 | + | ? | ? |
| DPU0060603 | 443 | DDB_G0286335 | + | DPU_G0060602 | - | PPA_G1280320 | + | DFA_G1510508 | + | ? | ? |
| DPU0060745 | 695 | DDB_G0269848 | + | DPU_G0060744 | - | PPA_G1301810 | + | DFA_G1518364 | + | ? | ? |
| DPU0060787 | 1286 | DDB_G0270188 | + | DPU_G0060786 | - | PPA_G1398436 | + | DFA_G1569046 | - | EHI_054230 | - |
| DPU0060797 | 56 | DDB_G0270192 | + | DPU_G0060796 | - | PPA_G1298712 | + | DFA_G1519414 | + | ? | ? |
| DPU0060871 | 2992 | DDB_G0268548 | + | DPU_G0060870 | - | PPA_G1346208 | + | DFA_G1570388 | - | ? | ? |
| DPU0060899 | 530 | DDB_G0279491 | + | DPU_G0060898 | - | PPA_G1301316 | + | DFA_G1504386 | + | ? | ? |
| DPU0060909 | 1903 | DDB_G0279049 | + | DPU_G0060908 | - | PPA_G1402850 | - | DFA_G1530008 | + | ? | ? |
| DPU0061289 | 2577 | DDB_G0291147 | + | DPU_G0061288 | - | PPA_G1364900 | + | DFA_G1524482 | - | ? | ? |
| DPU0061323 | 1556 | DDB_G0282411 | + | DPU_G0061322 | - | ? | ? | DFA_G1452942 | + | ? | ? |
| DPU0061375 | 580 | DDB_G0267852 | + | DPU_G0061374 | - | PPA_G1384348 | + | ? | ? | EHI_146200 | - |
| DPU0061449 | 884 | DDB_G0291095 | + | DPU_G0061448 | - | ? | ? | DFA_G1493322 | + | ? | ? |
| DPU0061639 | 1282 | DDB_G0281445 | + | DPU_G0061638 | - | PPA_G1283138 | + | DFA_G1549452 | - | ? | ? |
| DPU0062073 | 126 | DDB_G0267400 | + | DPU_G0062072 | - | PPA_G1324028 | - | DFA_G1527588 | + | ? | ? |
| DPU0062273 | 446 | DDB_G0268760 | + | DPU_G0062272 | - | PPA_G1376450 | - | DFA_G1576732 | + | ? | ? |
| DPU0062363 | 830 | DDB_G0277401 | + | DPU_G0062362 | - | PPA_G1397308 | - | DFA_G1507352 | + | EHI_189850 | - |
| DPU0062383 | 718 | DDB_G0277503 | + | DPU_G0062382 | - | PPA_G1408430 | + | DFA_G1549828 | + | ? | ? |
| DPU0062557 | 1575 | DDB_G0270786 | + | DPU_G0062556 | - | PPA_G1292332 | + | ? | ? | ? | ? |
| DPU0062931 | 1337 | DDB_G0272190 | + | DPU_G0062930 | - | ? | ? | DFA_G1587172 | + | EHI_015300 | - |
| DPU0062959 | 2353 | DDB_G0281743 | + | DPU_G0062958 | - | ? | ? | DFA_G1470910 | + | ? | ? |
| DPU0063013 | 961 | DDB_G0275299 | + | DPU_G0063012 | - | PPA_G1430790 | + | DFA_G1452306 | + | ? | ? |
| DPU0063131 | 1262 | DDB_G0287447 | + | DPU_G0063130 | - | PPA_G1371398 | + | DFA_G1539584 | - | ? | ? |
| DPU0063139 | 810 | DDB_G0287297 | + | DPU_G0063138 | - | PPA_G1291552 | + | DFA_G1519716 | - | ? | ? |
| DPU0063415 | 365 | DDB_G0268774 | + | DPU_G0063414 | - | PPA_G1427034 | + | DFA_G1507866 | + | ? | ? |
| DPU0063445 | 955 | DDB_G0286525 | + | DPU_G0063444 | - | PPA_G1276188 | + | DFA_G1525008 | + | ? | ? |
| DPU0063545 | 190 | DDB_G0269910 | + | DPU_G0063544 | - | PPA_G1311456 | + | DFA_G1457500 | + | ? | ? |
| DPU0063547 | 90 | DDB_G0270996 | + | DPU_G0063546 | - | PPA_G1311434 | + | DFA_G1457488 | + | ? | ? |
| DPU0063607 | 972 | DDB_G0284603 | + | DPU_G0063606 | - | PPA_G1345668 | - | DFA_G1450990 | + | ? | ? |
| DPU0063611 | 900 | DDB_G0285111 | + | DPU_G0063610 | - | PPA_G1363004 | + | DFA_G1593310 | - | EHI_064680 | - |
| DPU0063673 | 2657 | DDB_G0268394 | + | DPU_G0063672 | - | PPA_G1339060 | + | ? | ? | ? | ? |
| DPU0063693 | 1093 | DDB_G0269830 | + | DPU_G0063692 | - | PPA_G1411826 | + | ? | ? | ? | ? |
| DPU0064057 | 1871 | DDB_G0273445 | + | DPU_G0064056 | - | PPA_G1307596 | + | DFA_G1448974 | - | EHI_125930 | - |
| DPU0064193 | 692 | DDB_G0283115 | + | DPU_G0064192 | - | PPA_G1394940 | + | DFA_G1485566 | - | EHI_130850 | - |
| DPU0064255 | 1320 | DDB_G0271484 | + | DPU_G0064254 | - | PPA_G1345248 | + | DFA_G1498100 | - | ? | ? |
| DPU0064865 | 916 | DDB_G0281315 | + | DPU_G0064864 | - | PPA_G1369638 | + | DFA_G1508594 | - | EHI_182600 | - |
| DPU0064935 | 1218 | DDB_G0283439 | + | DPU_G0064934 | - | PPA_G1341862 | + | DFA_G1464462 | + | ? | ? |
| DPU0065151 | 405 | DDB_G0289571 | + | DPU_G0065150 | - | PPA_G1399614 | + | DFA_G1582082 | + | ? | ? |
| DPU0065167 | 441 | DDB_G0269994 | + | DPU_G0065166 | - | PPA_G1411760 | + | DFA_G1457442 | - | ? | ? |
| DPU0065727 | 113 | DDB_G0289181 | + | DPU_G0065726 | - | PPA_G1270618 | + | DFA_G1547372 | + | ? | ? |
| DPU0065963 | 378 | DDB_G0273177 | + | DPU_G0065962 | - | ? | ? | DFA_G1446626 | + | ? | ? |
| DPU0066021 | 1082 | DDB_G0279453 | + | DPU_G0066020 | - | ? | ? | DFA_G1593758 | + | ? | ? |
| DPU0066073 | 317 | DDB_G0269170 | + | DPU_G0066072 | - | PPA_G1386288 | + | DFA_G1465616 | + | ? | ? |
| DPU0066103 | 236 | DDB_G0280175 | + | DPU_G0066102 | - | PPA_G1397484 | + | DFA_G1568428 | - | ? | ? |
| DPU0066151 | 337 | DDB_G0289203 | + | DPU_G0066150 | - | PPA_G1305198 | + | DFA_G1544424 | - | ? | ? |
| DPU0066181 | 418 | DDB_G0277991 | + | DPU_G0066180 | - | PPA_G1377090 | + | DFA_G1595268 | + | ? | ? |
| DPU0066349 | 764 | DDB_G0268618 | + | DPU_G0066348 | - | PPA_G1396038 | + | DFA_G1583900 | - | EHI_188070 | - |
| DPU0066539 | 882 | DDB_G0270562 | + | DPU_G0066538 | - | PPA_G1313546 | + | DFA_G1441264 | + | ? | ? |
| DPU0066907 | 2304 | DDB_G0280047 | + | DPU_G0066906 | - | PPA_G1299888 | + | DFA_G1584350 | + | ? | ? |
| DPU0067581 | 1278 | DDB_G0281031 | + | DPU_G0067580 | - | PPA_G1368860 | + | DFA_G1544802 | + | ? | ? |
| DPU0067615 | 681 | DDB_G0279213 | + | DPU_G0067614 | - | PPA_G1372980 | - | DFA_G1514752 | + | ? | ? |
| DPU0067645 | 746 | DDB_G0269120 | + | DPU_G0067644 | - | PPA_G1389780 | + | ? | ? | EHI_128100 | - |
| DPU0067823 | 1419 | DDB_G0288875 | + | DPU_G0067822 | - | PPA_G1350564 | - | DFA_G1503892 | + | ? | ? |
| DPU0067897 | 412 | DDB_G0280215 | + | DPU_G0067896 | - | PPA_G1346762 | + | DFA_G1538210 | + | ? | ? |
| DPU0068067 | 69 | DDB_G0289271 | + | DPU_G0068066 | - | ? | ? | DFA_G1439296 | + | ? | ? |
| DPU0068069 | 646 | DDB_G0289269 | + | DPU_G0068068 | - | PPA_G1347758 | + | ? | ? | ? | ? |
| DPU0068113 | 1305 | DDB_G0273419 | + | DPU_G0068112 | - | PPA_G1365942 | ? | DFA_G1468590 | + | ? | ? |
| DPU0068113 | 2980 | DDB_G0273419 | + | DPU_G0068112 | - | PPA_G1365942 | - | DFA_G1468590 | + | ? | ? |
| DPU0068127 | 1608 | DDB_G0273453 | + | DPU_G0068126 | - | PPA_G1280448 | - | DFA_G1476770 | + | ? | ? |
| DPU0068201 | 160 | DDB_G0292892 | + | DPU_G0068200 | - | PPA_G1309704 | + | ? | ? | ? | ? |
| DPU0068767 | 1330 | DDB_G0272202 | + | DPU_G0068766 | - | PPA_G1295378 | + | DFA_G1478320 | - | ? | ? |
| DPU0068777 | 1928 | DDB_G0273209 | + | DPU_G0068776 | - | PPA_G1338526 | - | DFA_G1491016 | + | ? | ? |
| DPU0068777 | 2472 | DDB_G0273209 | + | DPU_G0068776 | - | PPA_G1338526 | - | DFA_G1491016 | + | ? | ? |
| DPU0068851 | 193 | DDB_G0271852 | + | DPU_G0068850 | - | PPA_G1326240 | + | DFA_G1505216 | + | ? | ? |
| DPU0069037 | 242 | DDB_G0291314 | + | DPU_G0069036 | - | PPA_G1327562 | + | DFA_G1502938 | + | EHI_156560 | - |
| DPU0069237 | 1695 | DDB_G0293124 | + | DPU_G0069236 | - | PPA_G1419210 | + | DFA_G1569700 | + | ? | ? |
| DPU0069261 | 67 | DDB_G0282731 | + | DPU_G0069260 | - | PPA_G1347066 | + | ? | ? | ? | ? |
| DPU0069277 | 493 | DDB_G0281983 | + | DPU_G0069276 | - | PPA_G1303036 | + | DFA_G1596466 | + | ? | ? |
| DPU0069383 | 1554 | DDB_G0292124 | + | DPU_G0069382 | - | ? | ? | DFA_G1506118 | + | ? | ? |
| DPU0069451 | 3355 | DDB_G0271844 | + | DPU_G0069450 | - | ? | ? | DFA_G1505752 | + | ? | ? |
| DPU0069637 | 184 | DDB_G0269890 | + | DPU_G0069636 | - | PPA_G1402836 | + | DFA_G1529992 | + | EHI_136490 | - |
| DPU0069785 | 1624 | DDB_G0288589 | + | DPU_G0069784 | - | ? | ? | DFA_G1555768 | + | ? | ? |
| DPU0069809 | 797 | DDB_G0280257 | + | DPU_G0069808 | - | PPA_G1345040 | + | DFA_G1446032 | + | ? | ? |
| DPU0069939 | 440 | DDB_G0271376 | + | DPU_G0069938 | - | PPA_G1398426 | - | DFA_G1451072 | + | ? | ? |
| DPU0069953 | 459 | DDB_G0271106 | + | DPU_G0069952 | - | PPA_G1373640 | + | DFA_G1594716 | + | EHI_159160 | - |
| DPU0070145 | 860 | DDB_G0281051 | + | DPU_G0070144 | - | PPA_G1389528 | + | ? | ? | EHI_136180 | - |
| DPU0070367 | 1856 | DDB_G0286293 | + | DPU_G0070366 | - | PPA_G1316974 | - | DFA_G1532164 | + | EHI_188910 | - |
| DPU0070369 | 153 | DDB_G0286295 | + | DPU_G0070368 | - | PPA_G1345604 | - | DFA_G1548736 | + | ? | ? |
| DPU0070369 | 361 | DDB_G0286295 | + | DPU_G0070368 | - | PPA_G1345604 | + | DFA_G1548736 | + | ? | ? |
| DPU0070519 | 987 | DDB_G0277143 | + | DPU_G0070518 | - | PPA_G1371816 | + | DFA_G1562882 | - | EHI_000240 | - |
| DPU0070837 | 4497 | DDB_G0292262 | + | DPU_G0070836 | - | PPA_G1415542 | + | DFA_G1578104 | + | ? | ? |
| DPU0070843 | 1363 | DDB_G0288753 | + | DPU_G0070842 | - | PPA_G1288990 | - | DFA_G1447216 | + | ? | ? |
| DPU0070963 | 322 | DDB_G0274141 | + | DPU_G0070962 | - | PPA_G1395018 | + | ? | ? | EHI_029370 | - |
| DPU0071141 | 1961 | DDB_G0282941 | + | DPU_G0071140 | - | PPA_G1330824 | + | DFA_G1450492 | - | ? | ? |
| DPU0071155 | 609 | DDB_G0283275 | + | DPU_G0071154 | - | PPA_G1323050 | + | DFA_G1450470 | - | ? | ? |
| DPU0071263 | 116 | DDB_G0284167 | + | DPU_G0071262 | - | PPA_G1306860 | + | DFA_G1510486 | - | ? | ? |
| DPU0071387 | 755 | DDB_G0283267 | + | DPU_G0071386 | - | PPA_G1323612 | + | DFA_G1554464 | + | ? | ? |
| DPU0071725 | 1902 | DDB_G0281343 | + | DPU_G0071724 | - | PPA_G1434952 | + | DFA_G1450284 | + | ? | ? |
| DPU0071729 | 440 | DDB_G0281369 | + | DPU_G0071728 | - | PPA_G1334294 | + | DFA_G1443674 | - | EHI_049740 | - |
| DPU0071811 | 3312 | DDB_G0282115 | + | DPU_G0071810 | - | PPA_G1367816 | + | DFA_G1532646 | - | ? | ? |
| DPU0071821 | 1108 | DDB_G0288375 | + | DPU_G0071820 | - | PPA_G1431514 | + | DFA_G1436506 | + | ? | ? |
| DPU0071961 | 85 | DDB_G0272406 | + | DPU_G0071960 | - | PPA_G1295638 | - | DFA_G1525448 | + | ? | ? |
| DPU0072237 | 1620 | DDB_G0281503 | + | DPU_G0072236 | - | ? | ? | DFA_G1501062 | + | ? | ? |
| DPU0072415 | 1316 | DDB_G0272638 | + | DPU_G0072414 | - | ? | ? | DFA_G1527212 | + | ? | ? |
| DPU0072475 | 519 | DDB_G0269546 | + | DPU_G0072474 | - | PPA_G1365720 | + | ? | ? | ? | ? |
| DPU0072491 | 2015 | DDB_G0269502 | + | DPU_G0072490 | - | PPA_G1286688 | + | DFA_G1472760 | + | ? | ? |
| DPU0072515 | 638 | DDB_G0277881 | + | DPU_G0072514 | - | PPA_G1388210 | + | DFA_G1518982 | + | ? | ? |
| DPU0072535 | 791 | DDB_G0269370 | + | DPU_G0072534 | - | PPA_G1417840 | + | DFA_G1469078 | - | EHI_077110 | - |
| DPU0072607 | 1023 | DDB_G0281213 | + | DPU_G0072606 | - | PPA_G1275064 | - | DFA_G1563132 | + | ? | ? |
| DPU0072663 | 2713 | DDB_G0293932 | + | DPU_G0072662 | - | PPA_G1383986 | + | DFA_G1557936 | - | ? | ? |
| DPU0072837 | 1604 | DDB_G0275467 | + | DPU_G0072836 | - | PPA_G1308500 | + | DFA_G1452452 | + | ? | ? |
| DPU0073081 | 823 | DDB_G0274331 | + | DPU_G0073080 | - | PPA_G1417280 | - | DFA_G1472580 | + | EHI_056390 | - |
| DPU0073175 | 1871 | DDB_G0291650 | + | DPU_G0073174 | - | PPA_G1404316 | + | ? | ? | ? | ? |
| DPU0073177 | 537 | DDB_G0291235 | + | DPU_G0073176 | - | PPA_G1404364 | + | DFA_G1518598 | + | EHI_148190 | - |
| DPU0073445 | 1982 | DDB_G0276719 | + | DPU_G0073444 | - | PPA_G1398886 | + | DFA_G1477990 | - | ? | ? |
| DPU0073497 | 801 | DDB_G0281563 | + | DPU_G0073496 | - | PPA_G1385094 | + | ? | ? | ? | ? |
| DPU0073677 | 1831 | DDB_G0279607 | + | DPU_G0073676 | - | PPA_G1399934 | - | DFA_G1539658 | + | ? | ? |
| DPU0073863 | 808 | DDB_G0280345 | + | DPU_G0073862 | - | PPA_G1371898 | + | DFA_G1522840 | - | ? | ? |
| DPU0074047 | 392 | DDB_G0285071 | + | DPU_G0074046 | - | PPA_G1359202 | - | DFA_G1464028 | + | ? | ? |
| DPU0074051 | 544 | DDB_G0284985 | + | DPU_G0074050 | - | PPA_G1359226 | + | DFA_G1464002 | + | EHI_108610 | - |
| DPU0074067 | 344 | DDB_G0276413 | + | DPU_G0074066 | - | PPA_G1402698 | + | DFA_G1505562 | + | ? | ? |
| DPU0074245 | 56 | DDB_G0267428 | + | DPU_G0074244 | - | PPA_G1283392 | - | DFA_G1563076 | + | EHI_099870 | - |
| DPU0074353 | 243 | DDB_G0282005 | + | DPU_G0074352 | - | PPA_G1401172 | + | DFA_G1523920 | + | ? | ? |
| DPU0074361 | 503 | DDB_G0275205 | + | DPU_G0074360 | - | PPA_G1382606 | + | ? | ? | ? | ? |
| DPU0074365 | 644 | DDB_G0275057 | + | DPU_G0074364 | - | PPA_G1382744 | + | DFA_G1492730 | + | ? | ? |
| DPU0074417 | 611 | DDB_G0267500 | + | DPU_G0074416 | - | PPA_G1426400 | - | DFA_G1575932 | + | ? | ? |
| DPU0074565 | 340 | DDB_G0278841 | + | DPU_G0074564 | - | PPA_G1287878 | + | ? | ? | ? | ? |
| DPU0074589 | 346 | DDB_G0282337 | + | DPU_G0074588 | - | PPA_G1273226 | + | DFA_G1578072 | + | ? | ? |
| DPU0074631 | 730 | DDB_G0280449 | + | DPU_G0074630 | - | PPA_G1398738 | + | ? | ? | ? | ? |
| DPU0074953 | 379 | DDB_G0280423 | + | DPU_G0074952 | - | ? | ? | DFA_G1451306 | + | ? | ? |
| DPU0075095 | 2763 | DDB_G0291994 | + | DPU_G0075094 | - | PPA_G1321228 | + | DFA_G1588874 | - | ? | ? |
| DPU0075207 | 1314 | DDB_G0292876 | + | DPU_G0075206 | - | PPA_G1389184 | + | DFA_G1567036 | + | ? | ? |
| DPU0075329 | 3129 | DDB_G0289755 | + | DPU_G0075328 | - | PPA_G1419580 | + | DFA_G1454974 | + | ? | ? |
| DPU0075371 | 416 | DDB_G0289515 | + | DPU_G0075370 | - | ? | ? | DFA_G1553356 | + | ? | ? |
| DPU0075553 | 138 | DDB_G0287939 | + | DPU_G0075552 | - | PPA_G1352088 | + | ? | ? | ? | ? |
| DPU0075619 | 170 | DDB_G0350235 | + | DPU_G0075618 | - | PPA_G1359094 | + | ? | ? | ? | ? |
| DPU0075743 | 846 | DDB_G0284883 | + | DPU_G0075742 | - | PPA_G1425650 | + | DFA_G1558438 | - | ? | ? |
| DPU0075777 | 292 | DDB_G0275289 | + | DPU_G0075776 | - | PPA_G1296140 | + | DFA_G1578284 | + | ? | ? |
| DPU0075789 | 2653 | DDB_G0276423 | + | DPU_G0075788 | - | PPA_G1330764 | - | DFA_G1461064 | + | ? | ? |
| **The intron gains in *D. discoideum*** | | | | | | | | | | | |
| DDB0191101 | 647-771 | DDB_G0289173 | + | DPU_G0067816 | - | PPA_G1300794 | - | DFA_G1493668 | - | EHI_073470 | - |
| DDB0191133 | 2322-2422 | DDB_G0268632 | + | DPU_G0053758 | - | PPA_G1403094 | - | DFA_G1472416 | - | EHI_199000 | - |
| DDB0191250 | 3120-3196 | DDB_G0284735 | + | DPU_G0053640 | - | PPA_G1416386 | - | DFA_G1471904 | - | EHI_088220 | - |
| DDB0191440 | 490-662 | DDB_G0287693 | + | DPU_G0067232 | - | PPA_G1307458 | - | DFA_G1577588 | - | EHI_147470 | - |
| DDB0191476 | 700-806 | DDB_G0283757 | + | DPU_G0071228 | - | PPA_G1364976 | - | DFA_G1565736 | - | EHI_146510 | - |
| DDB0191497 | 454-544 | DDB_G0269120 | + | DPU_G0067644 | - | PPA_G1389780 | - | DFA_G1536278 | - | EHI_128100 | - |
| DDB0201557 | 511-602 | DDB_G0288361 | + | DPU_G0070722 | - | PPA_G1411166 | - | DFA_G1577216 | - | EHI_124890 | - |
| DDB0201570 | 700-789 | DDB_G0292384 | + | DPU_G0074228 | - | PPA_G1404470 | - | DFA_G1465128 | - | EHI_114390 | - |
| DDB0201653 | 2591-2751 | DDB_G0291980 | + | DPU_G0075090 | - | PPA_G1321202 | - | DFA_G1588858 | - | EHI_197670 | - |
| DDB0201659 | 54-142 | DDB_G0293526 | + | DPU_G0054880 | - | PPA_G1415626 | - | DFA_G1578354 | - | EHI_070730 | - |
| DDB0215363 | 717-792 | DDB_G0293850 | + | DPU_G0056350 | - | PPA_G1284116 | - | DFA_G1458256 | - | EHI_029620 | - |
| DDB0216289 | 630-771 | DDB_G0292244 | + | DPU_G0059430 | - | PPA_G1387368 | - | DFA_G1538244 | - | EHI_178010 | - |
| DDB0216428 | 1609-1691 | DDB_G0280431 | + | DPU_G0052622 | - | PPA_G1317062 | - | DFA_G1500066 | - | EHI_050990 | - |
| DDB0229897 | 2205-2279 | DDB_G0275809 | + | DPU_G0064450 | - | PPA_G1386020 | - | DFA_G1522098 | - | EHI_172750 | - |
| DDB0229918 | 3175-3243 | DDB_G0268614 | + | DPU_G0053566 | - | PPA_G1413102 | - | DFA_G1566934 | - | EHI_193340 | - |
| DDB0229953 | 265-346 | DDB_G0272893 | + | DPU_G0058382 | - | PPA_G1282004 | - | DFA_G1584788 | - | EHI_020300 | - |
| DDB0230104 | 1394-1468 | DDB_G0290451 | + | DPU_G0061708 | - | PPA_G1282128 | - | DFA_G1593212 | - | EHI_128700 | - |
| DDB0230148 | 390-457 | DDB_G0290315 | + | DPU_G0071822 | - | PPA_G1358042 | - | DFA_G1446258 | - | EHI_051710 | - |
| DDB0230171 | 623-770 | DDB_G0287371 | + | DPU_G0075442 | - | PPA_G1352538 | - | DFA_G1534822 | - | EHI_014320 | - |
| DDB0231259 | 663-748 | DDB_G0278959 | + | DPU_G0071164 | - | PPA_G1357010 | - | DFA_G1577938 | - | EHI_193080 | - |
| DDB0231305 | 524-667 | DDB_G0272660 | + | DPU_G0059866 | - | PPA_G1324912 | - | DFA_G1506852 | - | EHI_092640 | - |
| DDB0231333 | 1991-2120 | DDB_G0275263 | + | DPU_G0074370 | - | PPA_G1417096 | - | DFA_G1586942 | - | EHI_126920 | - |
| DDB0232357 | 1408-1494 | DDB_G0272760 | + | DPU_G0062848 | - | PPA_G1433562 | - | DFA_G1457598 | - | EHI_118870 | - |
| DDB0233447 | 1804-1936 | DDB_G0283661 | + | DPU_G0058654 | - | PPA_G1384880 | - | DFA_G1566198 | - | EHI_175030 | - |
| DDB0233743 | 271-377 | DDB_G0267870 | + | DPU_G0056088 | - | PPA_G1346406 | - | DFA_G1580478 | - | EHI_192080 | - |
| DDB0235166 | 2514-2609 | DDB_G0277797 | + | DPU_G0053886 | - | PPA_G1341402 | - | DFA_G1597574 | - | EHI_048310 | - |
| DDB0235212 | 383-480 | DDB_G0271642 | + | DPU_G0066616 | - | PPA_G1331198 | - | DFA_G1538514 | - | EHI_009405 | - |
| DDB0237559 | 1096-1203 | DDB_G0272484 | + | DPU_G0072066 | - | PPA_G1427772 | - | DFA_G1470576 | - | EHI_110510 | - |
| DDB0237791 | 241-468 | DDB_G0276359 | + | DPU_G0070574 | - | PPA_G1400898 | - | DFA_G1562616 | - | EHI_197020 | - |
| DDB0238042 | 471-565 | DDB_G0281725 | + | DPU_G0062952 | - | PPA_G1341124 | - | DFA_G1565282 | - | EHI_003010 | - |
| DDB0252725 | 815-909 | DDB_G0295761 | + | DPU_G0070038 | - | PPA_G1298662 | - | DFA_G1457532 | - | EHI_130870 | - |
| DDB0266801 | 1609-1684 | DDB_G0273471 | + | DPU_G0068128 | - | PPA_G1321598 | - | DFA_G1467190 | - | EHI_110750 | - |
| DDB0267057 | 1799-1876 | DDB_G0292362 | + | DPU_G0073812 | - | PPA_G1406040 | - | DFA_G1473936 | - | EHI_185560 | - |
| DDB0305145 | 360-477 | DDB_G0276319 | + | DPU_G0069668 | - | PPA_G1335070 | - | DFA_G1480766 | - | EHI_148020 | - |
| DDB0305439 | 170-252 | DDB_G0275025 | + | DPU_G0069372 | - | PPA_G1380322 | - | DFA_G1470962 | - | EHI_021560 | - |
| DDB0305695 | 339-407 | DDB_G0278427 | + | DPU_G0054132 | - | PPA_G1422478 | - | DFA_G1589134 | - | EHI_110190 | - |
| DDB0305754 | 798-879 | DDB_G0280805 | + | DPU_G0054616 | - | PPA_G1371756 | - | DFA_G1522548 | - | EHI_142020 | - |
| DDB0307720 | 810-888 | DDB_G0282233 | + | DPU_G0071966 | - | PPA_G1315812 | - | DFA_G1521416 | - | EHI_152680 | - |
| DDB0307777 | 262-412 | DDB_G0271374 | + | DPU_G0069000 | - | PPA_G1425246 | - | DFA_G1593276 | - | EHI_113970 | - |
| DDB0347511 | 670-758 | DDB_G0281991 | + | DPU_G0069266 | - | PPA_G1346522 | - | DFA_G1457674 | - | EHI_130900 | - |
| **The intron gains in *D. purpureum*** | | | | | | | | | | | |
| DPU0051737 | 698-781 | DDB_G0286079 | - | DPU_G0051736 | + | PPA_G1350344 | - | DFA_G1566366 | - | EHI_023360 | - |
| DPU0052127 | 1564-1640 | DDB_G0290103 | - | DPU_G0052126 | + | PPA_G1384834 | - | DFA_G1457970 | - | EHI_152010 | - |
| DPU0053113 | 292-367 | DDB_G0287635 | - | DPU_G0053112 | + | PPA_G1284426 | - | DFA_G1490258 | - | EHI_040300 | - |
| DPU0053407 | 787-856 | DDB_G0267392 | - | DPU_G0053406 | + | PPA_G1288000 | - | DFA_G1538568 | - | EHI_097630 | - |
| DPU0053569 | 1446-1519 | DDB_G0267396 | - | DPU_G0053568 | + | PPA_G1286588 | - | DFA_G1448846 | - | EHI_119530 | - |
| DPU0054613 | 1122-1233 | DDB_G0280815 | - | DPU_G0054612 | + | PPA_G1371768 | - | DFA_G1532368 | - | EHI_155440 | - |
| DPU0054881 | 207-284 | DDB_G0293526 | - | DPU_G0054880 | + | PPA_G1415626 | - | DFA_G1578354 | - | EHI_070730 | - |
| DPU0054917 | 2251-2327 | DDB_G0270418 | - | DPU_G0054916 | + | PPA_G1405132 | - | DFA_G1513446 | - | EHI_120640 | - |
| DPU0055261 | 1871-1937 | DDB_G0290873 | - | DPU_G0055260 | + | PPA_G1432598 | - | DFA_G1538992 | - | EHI_052840 | - |
| DPU0055771 | 622-704 | DDB_G0275881 | - | DPU_G0055770 | + | PPA_G1302960 | - | DFA_G1569924 | - | EHI_017700 | - |
| DPU0055773 | 1489-1562 | DDB_G0275747 | - | DPU_G0055772 | + | PPA_G1302948 | - | DFA_G1560028 | - | EHI_007970 | - |
| DPU0056351 | 461-550 | DDB_G0293850 | - | DPU_G0056350 | + | PPA_G1284116 | - | DFA_G1458256 | - | EHI_029620 | - |
| DPU0056351 | 657-743 | DDB_G0293850 | - | DPU_G0056350 | + | PPA_G1284116 | - | DFA_G1458256 | - | EHI_029620 | - |
| DPU0056467 | 785-875 | DDB_G0292400 | - | DPU_G0056466 | + | PPA_G1276498 | - | DFA_G1485766 | - | EHI_194400 | - |
| DPU0057059 | 1454-1537 | DDB_G0277139 | - | DPU_G0057058 | + | PPA_G1322956 | - | DFA_G1564396 | - | EHI_124560 | - |
| DPU0058127 | 2668-2739 | DDB_G0278741 | - | DPU_G0058126 | + | PPA_G1404224 | - | DFA_G1527752 | - | EHI_136960 | - |
| DPU0058439 | 714-795 | DDB_G0282429 | - | DPU_G0058438 | + | PPA_G1307876 | - | DFA_G1516522 | - | EHI_053060 | - |
| DPU0058895 | 164-279 | DDB_G0285525 | - | DPU_G0058894 | + | PPA_G1317272 | - | DFA_G1436408 | - | EHI_155780 | - |
| DPU0059441 | 1058-1131 | DDB_G0284019 | - | DPU_G0059440 | + | PPA_G1416820 | - | DFA_G1552536 | - | EHI_049750 | - |
| DPU0059651 | 351-426 | DDB_G0286075 | - | DPU_G0059650 | + | PPA_G1391508 | - | DFA_G1574786 | - | EHI_126110 | - |
| DPU0059651 | 506-575 | DDB_G0286075 | - | DPU_G0059650 | + | PPA_G1391508 | - | DFA_G1574786 | - | EHI_126110 | - |
| DPU0059651 | 688-768 | DDB_G0286075 | - | DPU_G0059650 | + | PPA_G1391508 | - | DFA_G1574786 | - | EHI_126110 | - |
| DPU0059883 | 613-676 | DDB_G0272738 | - | DPU_G0059882 | + | PPA_G1296230 | - | DFA_G1516534 | - | EHI_199960 | - |
| DPU0060119 | 889-987 | DDB_G0277165 | - | DPU_G0060118 | + | PPA_G1398176 | - | DFA_G1500204 | - | EHI_110010 | - |
| DPU0060753 | 1148-1209 | DDB_G0270670 | - | DPU_G0060752 | + | PPA_G1311364 | - | DFA_G1457512 | - | EHI_008380 | - |
| DPU0061269 | 605-692 | DDB_G0291055 | - | DPU_G0061268 | + | PPA_G1301750 | - | DFA_G1493308 | - | EHI_000640 | - |
| DPU0061849 | 331-416 | DDB_G0288273 | - | DPU_G0061848 | + | PPA_G1352078 | - | DFA_G1456642 | - | EHI_185010 | - |
| DPU0061905 | 421-512 | DDB_G0270764 | - | DPU_G0061904 | + | PPA_G1289226 | - | DFA_G1519652 | - | EHI_010700 | - |
| DPU0062471 | 296-389 | DDB_G0269786 | - | DPU_G0062470 | + | PPA_G1342614 | - | DFA_G1590944 | - | EHI_050620 | - |
| DPU0062953 | 661-733 | DDB_G0281725 | - | DPU_G0062952 | + | PPA_G1341124 | - | DFA_G1565282 | - | EHI_003010 | - |
| DPU0063105 | 773-852 | DDB_G0288449 | - | DPU_G0063104 | + | PPA_G1311840 | - | DFA_G1440826 | - | EHI_095860 | - |
| DPU0065347 | 358-430 | DDB_G0290919 | - | DPU_G0065346 | + | PPA_G1357580 | - | DFA_G1550132 | - | EHI_106280 | - |
| DPU0065603 | 572-668 | DDB_G0285881 | - | DPU_G0065602 | + | PPA_G1397230 | - | DFA_G1559824 | - | EHI_035600 | - |
| DPU0067021 | 798-874 | DDB_G0277877 | - | DPU_G0067020 | + | PPA_G1302378 | - | DFA_G1555700 | - | EHI_178910 | - |
| DPU0067359 | 1123-1193 | DDB_G0269388 | - | DPU_G0067358 | + | PPA_G1415946 | - | DFA_G1457214 | - | EHI_082080 | - |
| DPU0067365 | 8777-8855 | DDB_G0279083 | - | DPU_G0067364 | + | PPA_G1279548 | - | DFA_G1591838 | - | EHI_148770 | - |
| DPU0067657 | 1380-1461 | DDB_G0267854 | - | DPU_G0067656 | + | PPA_G1329372 | - | DFA_G1536524 | - | EHI_127420 | - |
| DPU0067811 | 5841-5927 | DDB_G0276105 | - | DPU_G0067810 | + | PPA_G1418214 | - | DFA_G1474176 | - | EHI_085980 | - |
| DPU0068761 | 923-988 | DDB_G0272296 | - | DPU_G0068760 | + | PPA_G1295352 | - | DFA_G1478298 | - | EHI_031410 | - |
| DPU0069655 | 1194-1267 | DDB_G0269046 | - | DPU_G0069654 | + | PPA_G1393036 | - | DFA_G1494662 | - | EHI_014030 | - |
| DPU0069891 | 1006-1082 | DDB_G0289473 | - | DPU_G0069890 | + | PPA_G1327690 | - | DFA_G1556730 | - | EHI_030830 | - |
| DPU0070121 | 2869-2932 | DDB_G0292302 | - | DPU_G0070120 | + | PPA_G1410326 | - | DFA_G1578494 | - | EHI_103420 | - |
| DPU0071229 | 815-888 | DDB_G0283757 | - | DPU_G0071228 | + | PPA_G1364976 | - | DFA_G1565736 | - | EHI_146510 | - |
| DPU0071985 | 1189-1262 | DDB_G0275145 | - | DPU_G0071984 | + | PPA_G1424546 | - | DFA_G1450222 | - | EHI_122760 | - |
| DPU0072067 | 408-474 | DDB_G0272484 | - | DPU_G0072066 | + | PPA_G1427772 | - | DFA_G1470576 | - | EHI_110510 | - |
| DPU0072761 | 1226-1299 | DDB_G0268034 | - | DPU_G0072760 | + | PPA_G1269762 | - | DFA_G1535892 | - | EHI_167060 | - |
| DPU0073043 | 660-740 | DDB_G0274113 | - | DPU_G0073042 | + | PPA_G1316836 | - | DFA_G1527732 | - | EHI_127200 | - |
| DPU0073415 | 198-276 | DDB_G0277157 | - | DPU_G0073414 | + | PPA_G1293410 | - | DFA_G1498586 | - | EHI_000610 | - |
| DPU0073895 | 1981-2046 | DDB_G0289117 | - | DPU_G0073894 | + | PPA_G1344426 | - | DFA_G1589900 | - | EHI_110810 | - |
| DPU0074371 | 1477-1575 | DDB_G0275263 | - | DPU_G0074370 | + | PPA_G1417096 | - | DFA_G1586942 | - | EHI_126920 | - |
| DPU0074371 | 1907-1982 | DDB_G0275263 | - | DPU_G0074370 | + | PPA_G1417096 | - | DFA_G1586942 | - | EHI_126920 | - |
| DPU0074371 | 2025-2090 | DDB_G0275263 | - | DPU_G0074370 | + | PPA_G1417096 | - | DFA_G1586942 | - | EHI_126920 | - |
| DPU0074415 | 1293-1377 | DDB_G0267436 | - | DPU_G0074414 | + | PPA_G1268044 | - | DFA_G1503280 | - | EHI_047760 | - |
| DPU0074469 | 925-999 | DDB_G0290055 | - | DPU_G0074468 | + | PPA_G1289952 | - | DFA_G1588508 | - | EHI_039830 | - |
| DPU0075443 | 84-188 | DDB_G0287371 | - | DPU_G0075442 | + | PPA_G1352538 | - | DFA_G1534822 | - | EHI_014320 | - |
| DPU0075543 | 653-726 | DDB_G0287607 | - | DPU_G0075542 | + | PPA_G1426606 | - | DFA_G1562682 | - | EHI_128450 | - |
| DPU0075771 | 714-810 | DDB_G0275049 | - | DPU_G0075770 | + | PPA_G1321990 | - | DFA_G1580794 | - | EHI_163540 | - |
| DPU0075799 | 317-428 | DDB_G0276473 | - | DPU_G0075798 | + | PPA_G1321042 | - | DFA_G1510038 | - | EHI_098800 | - |
