# Supplementary material for: Evaluation of the mechanisms of intron loss and gain in the social amoebae Dictyostelium
Source: BMC Evol Biol. 2015 Dec 18;15:286. doi: 10.1186/s12862-015-0567-y (PMC4683709; doi:10.1186/s12862-015-0567-y)
Supplement: Additional file 2: Table S2. — Results of gene ontology enrichment analysis. (DOC 203 kb) [file 12862_2015_567_MOESM2_ESM.doc]

Additional File 2 for

**Evaluation of the mechanisms of intron loss and gain in the social amoebae *Dictyostelium***

Ming-Yue Ma, Xun-Ru Che, Andrea Porceddu, and Deng-Ke Niu

Table S2. Enrichment of GO terms among the intron-lost genes and putative intron-gained genes. Only *P*-values lower than 0.01 are shown in this table.

| Gene Ontology terms | Number of target genes | Number of all genes | *P*-value |
| --- | --- | --- | --- |
| **Intron lost genes (N = 586)** |  |  |  |
| Cellular process | 347 (59.2%) | 4281 (35.4%) | 1.9 × 10^-30^ |
| Intracellular | 304 (51.9%) | 3699 (30.6%) | 6.6 × 10^-26^ |
| Cell part | 309 (52.7%) | 3872 (32.%) | 3.9 × 10^-24^ |
| Cell | 310 (52.9%) | 3906 (32.3%) | 8.2 × 10^-24^ |
| Intracellular part | 280 (47.8%) | 3460 (28.6%) | 1.4 × 10^-21^ |
| Metabolic process | 325 (55.5%) | 4336 (35.8%) | 2.7 × 10^-20^ |
| Single-organism cellular process | 225 (38.4%) | 2687 (22.2%) | 7.5 × 10^-17^ |
| Primary metabolic process | 238 (40.6%) | 2935 (24.3%) | 2.2 × 10^-16^ |
| Single-organism process | 255 (43.5%) | 3238 (26.8%) | 2.3 × 10^-16^ |
| Catalytic activity | 253 (43.2%) | 3238 (26.8%) | 3.8 × 10^-16^ |
| Organic substance metabolic process | 246 (42.0%) | 3166 (26.2%) | 1.1 × 10^-14^ |
| Cellular metabolic process | 230 (39.2%) | 2883 (23.8%) | 1.2 × 10^-14^ |
| Cytoplasm | 203 (34.6%) | 2496 (20.6%) | 1.1 × 10^-13^ |
| Intracellular Organelle | 215 (36.7%) | 2751 (22.7%) | 8.2 × 10^-13^ |
| Organelle | 216 (36.9%) | 2774 (0.229) | 1.0 × 10^-12^ |
| Protein Complex | 103 (17.6%) | 1000 (0.083) | 1.6 × 10^-11^ |
| Intracellular Membrane-Bounded Organelle | 192 (32.8%) | 2485 (0.205) | 1.9 × 10^-10^ |
| Membrane-Bounded Organelle | 192 (32.8%) | 2502 (0.207) | 3.9 × 10^-10^ |
| Ion binding | 180 (30.7%) | 2292 (18.9%) | 4.9 × 10^-10^ |
| Anion binding | 120 (20.5%) | 1329 (11.0%) | 1.5 × 10^-9^ |
| Cytoplasmic part | 151 (25.8%) | 1850 (15.3%) | 2.7 × 10^-9^ |
| Binding | 249 (42.5%) | 3588 (29.7%) | 3.0 × 10^-9^ |
| Macromolecular Complex | 115 (19.6%) | 1273 (10.5%) | 3.3 × 10^-9^ |
| Intracellular signal transduction | 56 (9.6%) | 437 (3.6%) | 1.8 × 10^-8^ |
| Cellular macromolecule metabolic process | 168 (28.7%) | 2155 (17.8%) | 2.2 × 10^-8^ |
| Purine ribonucleoside triphosphate binding | 102 (17.4%) | 1109 (9.2%) | 3.0 × 10^-8^ |
| Purine nucleoside binding | 102 (17.4%) | 1110 (9.2%) | 3.2 × 10^-8^ |
| Purine ribonucleoside binding | 102 (17.4%) | 1110 (9.2%) | 3.2 × 10^-8^ |
| Ribonucleotide binding | 104 (17.7%) | 1143 (9.4%) | 3.6 × 10^-8^ |
| Ribonucleoside binding | 102 (17.4%) | 1113 (9.2%) | 3.7 × 10^-8^ |
| Intracellular Organelle Part | 120 (20.5%) | 1413 (11.7%) | 6.1 × 10^-8^ |
| Macromolecule Metabolic Process | 182 (31.1%) | 2435 (20.1%) | 8.7 × 10^-8^ |
| Organelle Part | 120 (20.5%) | 1430 (11.8%) | 1.3 × 10^-7^ |
| Regulation of gtpase activity | 34 (5.8%) | 208 (1.7%) | 3.5 × 10^-7^ |
| Signal transduction | 66 (11.3%) | 613 (5.1%) | 5.8 × 10^-7^ |
| Single organism signaling | 66 (11.3%) | 613 (5.1%) | 5.8 × 10^-7^ |
| Signaling | 66 (11.3%) | 614 (5.1%) | 6.2 × 10^-7^ |
| Small gtpase mediated signal transduction | 40 (6.8%) | 284 (2.3%) | 9.7 × 10^-7^ |
| Positive regulation of gtpase activity | 32 (5.5%) | 196 (1.6%) | 1.2 × 10^-6^ |
| Localization | 200 (34.1%) | 2844 (23.5%) | 1.5 × 10^-6^ |
| Cellular response to stimulus | 103 (17.6%) | 1184 (9.8%) | 1.7 × 10^-6^ |
| Cell communication | 87 (14.8%) | 944 (7.8%) | 2.5 × 10^-6^ |
| Response to stimulus | 117 (20.0%) | 1423 (11.8%) | 3.1 × 10^-6^ |
| Positive regulation of hydrolase activity | 32 (5.5%) | 204 (1.7%) | 3.3 × 10^-6^ |
| Regulation of hydrolase activity | 35 (6.0%) | 239 (2.0%) | 3.9 × 10^-6^ |
| Establishment of localization | 194 (33.1%) | 2779 (23.0%) | 5.9 × 10^-6^ |
| Regulation of catalytic activity | 45 (7.7%) | 364 (3.0%) | 5.9 × 10^-6^ |
| Regulation of cellular process | 106 (18.1%) | 1266 (10.5%) | 7.8 × 10^-6^ |
| Transport | 193 (32.9%) | 2774 (22.9%) | 8.7 × 10^-6^ |
| Protein metabolic process | 113 (19.3%) | 1388 (11.5%) | 1.1 × 10^-5^ |
| Regulation of molecular function | 45 (7.7%) | 373 (3.1%) | 1.3 × 10^-5^ |
| Biological regulation | 122 (20.8%) | 1543 (12.8%) | 1.4 × 10^-5^ |
| Positive regulation of catalytic activity | 37 (6.3%) | 283 (2.3%) | 3.2 × 10^-5^ |
| Positive regulation of molecular function | 37 (6.3%) | 289 (2.4%) | 5.6 × 10^-5^ |
| Macromolecule localization | 49 (8.4%) | 446 (3.7%) | 5.8 × 10^-5^ |
| Phosphate-containing compound metabolic process | 72 (12.3%) | 779 (6.4%) | 6.4 × 10^-5^ |
| Cellular protein metabolic process | 101 (17.2%) | 1237 (10.2%) | 6.8 × 10^-5^ |
| Single-organism metabolic process | 119 (20.3%) | 1537 (12.7%) | 7.2 × 10^-5^ |
| Regulation of biological process | 113 (19.3%) | 1440 (11.9%) | 8.3 × 10^-5^ |
| Phosphorus metabolic process | 72 (12.3%) | 786 (6.5%) | 9.1 × 10^-5^ |
| Vesicle-mediated transport | 165 (28.2%) | 2359 (19.5%) | 1.3 × 10^-4^ |
| Regulation of response to stimulus | 31 (5.3%) | 226 (1.9%) | 1.4 × 10^-4^ |
| Protein localization | 44 (7.5%) | 394 (3.3%) | 1.7 × 10^-4^ |
| Regulation of Ras protein signal transduction | 16 (2.7%) | 72 (0.6%) | 2.4 × 10^-4^ |
| Regulation of Rho protein signal transduction | 13 (2.2%) | 48 (0.4%) | 2.7 × 10^-4^ |
| Positive regulation of biological process | 48 (8.2%) | 457 (3.8%) | 3.1 × 10^-4^ |
| Regulation of small gtpase mediated signal transduction | 17 (2.9%) | 83 (0.7%) | 3.5 × 10^-4^ |
| Cellular protein modification process | 75 (12.8%) | 861 (7.1%) | 3.6 × 10^-4^ |
| Macropinocytosis | 153 (26.1%) | 2179 (18.0%) | 3.6 × 10^-4^ |
| Protein modification process | 75 (12.8%) | 861 (7.1%) | 3.6 × 10^-4^ |
| Macromolecule modification | 81 (13.8%) | 959 (7.9%) | 4.2 × 10^-4^ |
| Cellular component organization or biogenesis | 86 (14.7%) | 1044 (8.6%) | 5.2 × 10^-4^ |
| Cellular localization | 47 (8.0%) | 451 (3.7%) | 5.2 × 10^-4^ |
| Cellular component organization | 77 (13.1%) | 904 (7.5%) | 6.1 × 10^-4^ |
| Pinocytosis | 153 (26.1%) | 2203 (18.2%) | 7.3 × 10^-4^ |
| Rho protein signal transduction | 13 (2.2%) | 52 (0.4%) | 7.7 × 10^-4^ |
| Endocytosis | 156 (26.6%) | 2269 (18.8%) | 0.0010 |
| Positive regulation of metabolic process | 41 (7.0%) | 382 (3.2%) | 0.0013 |
| Endomembrane System | 50 (8.5%) | 536 (4.4%) | 0.0014 |
| Organic substance catabolic process | 47 (8.0%) | 468 (3.9%) | 0.0015 |
| Ras protein signal transduction | 23 (3.9%) | 157 (1.3%) | 0.0019 |
| Nitrogen compound metabolic process | 124 (21.2%) | 1725 (14.3%) | 0.0021 |
| Regulation of cell communication | 27 (4.6%) | 207 (1.7%) | 0.0025 |
| Cellular lipid metabolic process | 29 (4.9%) | 232 (1.9%) | 0.0025 |
| Lipid catabolic process | 12 (2.0%) | 49 (0.4%) | 0.0026 |
| Intracellular Organelle Lumen | 43 (7.3%) | 448 (3.7%) | 0.0033 |
| Membrane-Enclosed Lumen | 44 (7.5%) | 467 (3.9%) | 0.0042 |
| Cellular biosynthetic process | 89 (15.2%) | 1148 (9.5%) | 0.0042 |
| Biosynthetic process | 93 (15.9%) | 1215 (10.0%) | 0.0043 |
| Organelle Lumen | 43 (7.3%) | 453 (3.7%) | 0.0043 |
| Organic substance biosynthetic process | 90 (15.4%) | 1166 (9.6%) | 0.0044 |
| Organic substance transport | 45 (7.7%) | 464 (3.8%) | 0.0064 |
| Organelle organization | 57 (9.7%) | 644 (5.3%) | 0.0064 |
| Lipid metabolic process | 33 (5.6%) | 297 (2.5%) | 0.0070 |
| Establishment of protein localization | 37 (6.3%) | 353 (2.9%) | 0.0077 |
| Phosphorylation | 46 (7.8%) | 483 (4.0%) | 0.0081 |
| Small molecule metabolic process | 53 (9.0%) | 590 (4.9%) | 0.0091 |
| Regulation of signal transduction | 22 (3.8%) | 161 (1.3%) | 0.0099 |
|  |  |  |  |
| **Putative intron gained genes (N = 86)** |  |  |  |
| Intracellular Part | 61 (70.9%) | 3460 (28.6%) | 4.4 × 10^-14^ |
| Metabolic process | 67 (77.9%) | 4336 (35.8%) | 5.3 × 10^-13^ |
| Intracellular | 61 (70.9%) | 3699 (30.6%) | 1.3 × 10^-12^ |
| Cell Part | 62 (72.1%) | 3872 (32.0%) | 2.5 × 10^-12^ |
| Cell | 62 (72.1%) | 3906 (32.3%) | 3.9 × 10^-12^ |
| Vesicle-mediated transport | 47 (54.7%) | 2359 (19.5%) | 1.4 × 10^-10^ |
| Intracellular Organelle | 50 (58.1%) | 2751 (22.7%) | 1.4 × 10^-10^ |
| Organelle | 50 (58.1%) | 2774 (22.9%) | 1.9 × 10^-10^ |
| Binding | 57 (66.3%) | 3588 (29.7%) | 2.3 × 10^-10^ |
| Cellular process | 63 (73.3%) | 4281 (35.4%) | 2.7 × 10^-10^ |
| Localization | 51 (59.3%) | 2844 (23.5%) | 3.8 × 10^-10^ |
| Transport | 50 (58.1%) | 2774 (22.9%) | 6.6 × 10^-10^ |
| Establishment of localization | 50 (58.1%) | 2779 (23.0%) | 7.1 × 10^-10^ |
| Endocytosis | 45 (52.3%) | 2269 (18.8%) | 8.1 × 10^-10^ |
| Cytoplasm | 46 (53.5%) | 2496 (20.6%) | 1.7 × 10^-9^ |
| Macropinocytosis | 43 (50.0%) | 2179 (18.0%) | 4.4 × 10^-9^ |
| Macromolecular Complex | 32 (37.2%) | 1273 (10.5%) | 5.1 × 10^-9^ |
| Primary metabolic process | 50 (58.1%) | 2935 (24.3%) | 6.2 × 10^-9^ |
| Pinocytosis | 43 (50.0%) | 2203 (18.2%) | 6.4 × 10^-9^ |
| Ion binding | 42 (48.8%) | 2292 (18.9%) | 3.4 × 10^-8^ |
| Non-Membrane-Bounded Organelle | 22 (25.6%) | 656 (5.4%) | 6.6 × 10^-8^ |
| Intracellular Non-Membrane-Bounded Organelle | 22 (25.6%) | 656 (5.4%) | 6.6 × 10^-8^ |
| Organic substance metabolic process | 50 (58.1%) | 3166 (26.2%) | 1.2 × 10^-7^ |
| Ribonucleoside binding | 28 (32.6%) | 1113 (9.2%) | 1.4 × 10^-7^ |
| Nucleoside binding | 28 (32.6%) | 1114 (9.2%) | 1.4 × 10^-7^ |
| Cellular nitrogen compound metabolic process | 34 (39.5%) | 1587 (13.1%) | 2.5 × 10^-7^ |
| Catalytic activity | 49 (57.0%) | 3238 (26.8%) | 3.3 × 10^-7^ |
| Carbohydrate derivative binding | 28 (32.6%) | 1166 (9.6%) | 3.9 × 10^-7^ |
| Purine ribonucleoside triphosphate binding | 27 (31.4%) | 1109 (9.2%) | 6.1 × 10^-7^ |
| Purine nucleoside binding | 27 (31.4%) | 1110 (9.2%) | 6.3 × 10^-7^ |
| Purine ribonucleoside binding | 27 (31.4%) | 1110 (9.2%) | 6.3 × 10^-7^ |
| Purine ribonucleotide binding | 27 (31.4%) | 1129 (9.3%) | 9.0 × 10^-7^ |
| Purine nucleotide binding | 27 (31.4%) | 1131 (9.3%) | 9.4 × 10^-7^ |
| Ribonucleotide binding | 27 (31.4%) | 1143 (9.4%) | 1.2 × 10^-6^ |
| Intracellular Membrane-Bounded Organelle | 41 (47.7%) | 2485 (20.5%) | 1.6 × 10^-6^ |
| Membrane-Bounded Organelle | 41 (47.7%) | 2502 (20.7%) | 1.9 × 10^-6^ |
| Nitrogen compound metabolic process | 34 (39.5%) | 1725 (14.3%) | 2.3 × 10^-6^ |
| Macromolecule metabolic process | 41 (47.7%) | 2435 (20.1%) | 2.8 × 10^-6^ |
| Cellular metabolic process | 45 (52.3%) | 2883 (23.8%) | 3.3 × 10^-6^ |
| Cellular macromolecule metabolic process | 38 (44.2%) | 2155 (17.8%) | 4.1 × 10^-6^ |
| Small molecule binding | 29 (33.7%) | 1398 (11.6%) | 5.2 × 10^-6^ |
| ATP binding | 22 (25.6%) | 866 (7.2%) | 1.1 × 10^-5^ |
| Nucleotide binding | 28 (32.6%) | 1367 (11.3%) | 1.3 × 10^-5^ |
| Cytoplasmic Part | 33 (38.4%) | 1850 (15.3%) | 1.5 × 10^-5^ |
| Nucleoside phosphate binding | 28 (32.6%) | 1367 (11.3%) | 1.3 × 10^-5^ |
| Adenyl ribonucleotide binding | 22 (25.6%) | 884 (7.3%) | 1.6 × 10^-5^ |
| Adenyl nucleotide binding | 22 (25.6%) | 885 (7.3%) | 1.6 × 10^-5^ |
| Organic cyclic compound binding | 37 (43.0%) | 2257 (18.7%) | 1.7 × 10^-5^ |
| Heterocyclic compound binding | 37 (43.0%) | 2257 (18.7%) | 1.7 × 10^-5^ |
| Anion binding | 27 (31.4%) | 1329 (11.0%) | 2.8 × 10^-5^ |
| Protein metabolic process | 28 (32.6%) | 1388 (11.5%) | 5.4 × 10^-5^ |
| Peptide Metabolic Process | 13 (15.1%) | 299 (2.5%) | 5.8 × 10^-5^ |
| Intracellular Organelle Part | 27 (31.4%) | 1413 (11.7%) | 8.8 × 10^-5^ |
| Structural Molecule Activity | 10 (11.6%) | 189 (1.6%) | 9.5 × 10^-5^ |
| Organelle Part | 27 (31.4%) | 1430 (11.8%) | 1.1 × 10^-4^ |
| Hydrolase Activity | 25 (29.1%) | 1260 (10.4%) | 1.3 × 10^-4^ |
| Translation | 12 (14.0%) | 272 (2.2%) | 1.5 × 10^-4^ |
| Atpase activity | 11 (12.8%) | 251 (2.1%) | 1.6 × 10^-4^ |
| Single-organism cellular process | 40 (46.5%) | 2687 (22.2%) | 1.6 × 10^-4^ |
| Peptide biosynthetic process | 12 (14.0%) | 278 (2.3%) | 1.9 × 10^-4^ |
| Nucleoside-triphosphatase activity | 14 (16.3%) | 435 (3.6%) | 2.2 × 10^-4^ |
| Cellular amide metabolic process | 13 (15.1%) | 338 (2.8%) | 2.3 × 10^-4^ |
| Structural constituent of ribosome | 8 (9.3%) | 122 (1.0%) | 2.5 × 10^-4^ |
| Cellular nitrogen compound biosynthetic process | 20 (23.3%) | 821 (6.8%) | 2.9 × 10^-4^ |
| Pyrophosphatase activity | 14 (16.3%) | 446 (3.7%) | 3.0 × 10^-4^ |
| Hydrolase activity, acting on acid anhydrides, in phosphorus-containing anhydrides | 14 (16.3%) | 446 (3.7%) | 3.0 × 10^-4^ |
| Hydrolase activity, acting on acid anhydrides | 14 (16.3%) | 449 (3.7%) | 3.3 × 10^-4^ |
| Amide biosynthetic process | 12 (14.0%) | 297 (2.5%) | 3.9 × 10^-4^ |
| Single-organism process | 44 (51.2%) | 3238 (26.8%) | 4.0 × 10^-4^ |
| Organonitrogen Compound Biosynthetic Process | 15 (17.4%) | 480 (4.0%) | 4.2 × 10^-4^ |
| Protein Complex | 21 (24.4%) | 1000 (8.3%) | 4.8 × 10^-4^ |
| Regulation Of DNA Metabolic Process | 4 (4.7%) | 13 (0.1%) | 5.5 × 10^-4^ |
| Ribosome | 8 (9.3%) | 138 (1.1%) | 6.0 × 10^-4^ |
| Cellular macromolecule biosynthetic process | 19 (22.1%) | 796 (6.6%) | 7.9 × 10^-4^ |
| Biosynthetic process | 24 (27.9%) | 1215 (10.0%) | 8.1 × 10^-4^ |
| Macromolecule biosynthetic process | 19 (22.1%) | 807 (6.7%) | 9.6 × 10^-4^ |
| Cytoplasmic vesicle | 14 (16.3%) | 497 (4.1%) | 9.9 × 10^-4^ |
| Cellular biosynthetic process | 23 (26.7%) | 1148 (9.5%) | 0.0011 |
| Cellular protein metabolic process | 24 (27.9%) | 1237 (10.2%) | 0.0011 |
| Vesicle | 14 (16.3%) | 508 (4.2%) | 0.0013 |
| Organic substance biosynthetic process | 23 (26.7%) | 1166 (9.6%) | 0.0014 |
| Organonitrogen compound metabolic process | 17 (19.8%) | 684 (5.7%) | 0.0017 |
| Intracellular Ribonucleoprotein complex | 11 (12.8%) | 336 (2.8%) | 0.0025 |
| Ribonucleoprotein complex | 11 (12.8%) | 336 (2.8%) | 0.0025 |
| Nucleobase-containing compound metabolic process | 24 (27.9%) | 1305 (10.8%) | 0.0028 |
| Vesicle Coat | 4 (4.7%) | 26 (0.2%) | 0.0033 |
| Cytoplasmic membrane-Bounded Vesicle | 13 (15.1%) | 487 (4.0%) | 0.0038 |
| Response to stimulus | 25 (29.1%) | 1423 (11.8%) | 0.0039 |
| Membrane-bounded vesicle | 13 (15.1%) | 489 (4.0%) | 0.0039 |
| Mutsalpha complex | 2 (2.3%) | 2 (0.0%) | 0.0051 |
| Single base insertion or deletion binding | 2 (2.3%) | 2 (0.0%) | 0.0055 |
| Heterocycle metabolic process | 24 (27.9%) | 1361 (11.2%) | 0.0058 |
| Cellular aromatic compound metabolic process | 24 (27.9%) | 1373 (11.3%) | 0.0067 |
| Movement of cell or subcellular component | 7 (8.1%) | 119 (1.0%) | 0.0072 |
| DNA repair | 8 (9.3%) | 165 (1.4%) | 0.0074 |
| Endocytic vesicle | 11 (12.8%) | 386 (3.2%) | 0.0089 |
| Organic cyclic compound metabolic process | 24 (27.9%) | 1404 (11.6%) | 0.0097 |
