# Supplementary material for: Evaluation of the mechanisms of intron loss and gain in the social amoebae Dictyostelium
Source: BMC Evol Biol. 2015 Dec 18;15:286. doi: 10.1186/s12862-015-0567-y (PMC4683709; doi:10.1186/s12862-015-0567-y)
Supplement: Additional file 3: Figure S1. — Comparison of the intron sizes between Dictyostelium discoideum and Dictyostelium purpureum. All the introns annotated in these two genomes were compared, including 15,510 in Dictyostelium discoideum and 18,412 in Dictyostelium purpureum; Table S3. The abundance of repetitive sequences and introns in Dictyostelium discoideum and Dictyostelium purpureum. Table S4. Exonic sequences flanking lost introns have higher GC contents than those flanking conserved introns in most previously studied organisms; Table S5. At discordant intron positions, the relative GC content of exonic sequences flanking lost introns compared with the exonic sequences flanking extant introns of the sister species; Table S6. The GC content of exonic sequences flanking extant introns at discordant intron positions compared with those flanking conserved intron sites of the same species; Table S7. Version numbers and source databases of the plant and animal genomes used in this study. (DOC 540 kb) [file 12862_2015_567_MOESM3_ESM.doc]

Additional File 3 for

**Evaluation of the mechanisms of intron loss and gain in the social amoebae *Dictyostelium***

Ming-Yue Ma, Xun-Ru Che, Andrea Porceddu, and Deng-Ke Niu


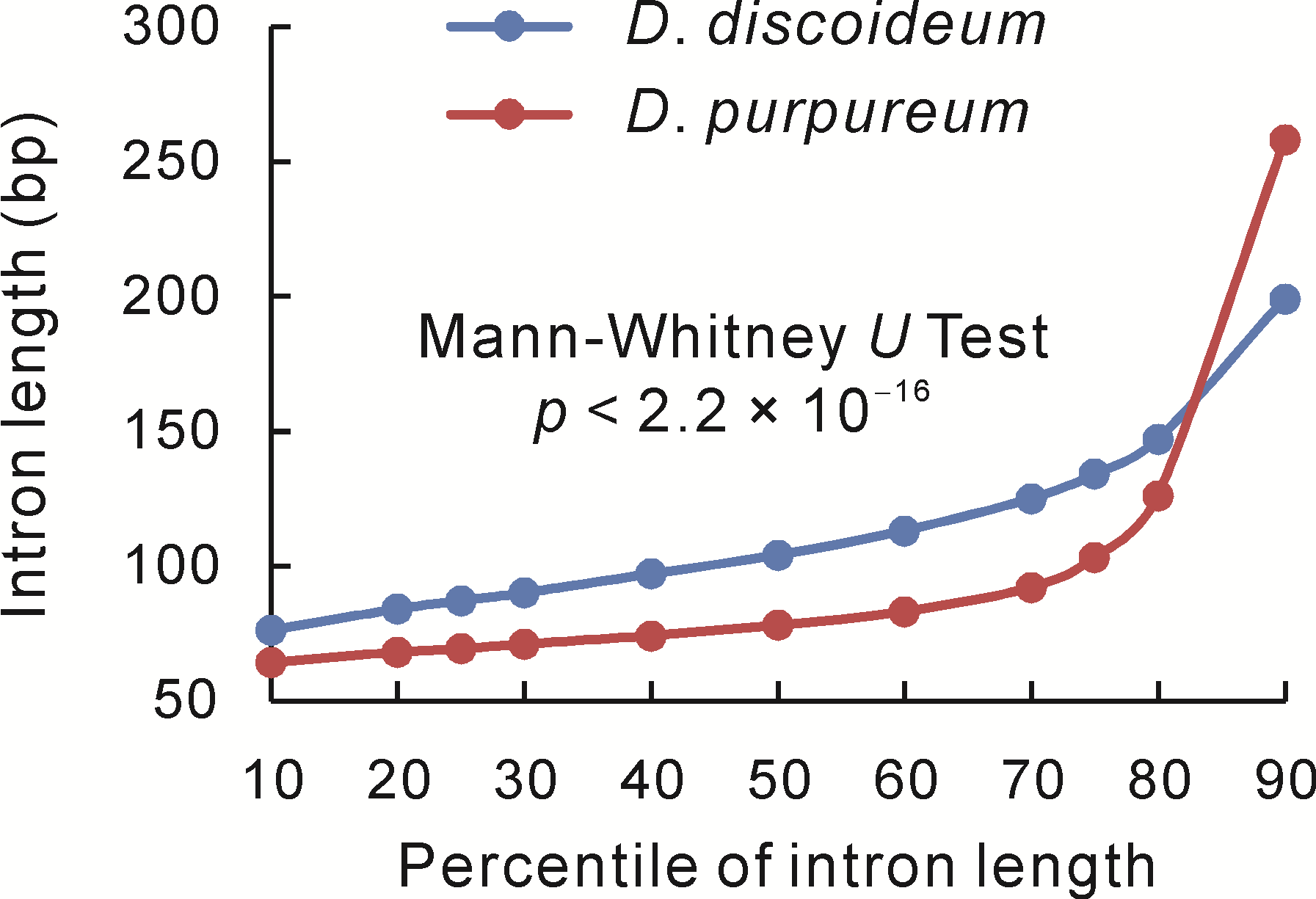


Figure S1. Comparison of the intron sizes between *D. discoideum* and *D. purpureum*. All the introns annotated in these two genomes were compared, including 15,510 in *D. discoideum* and 18,412 in *D. purpureum*.

Table S3. The abundance of repetitive sequences and introns in *D. discoideum* and *D. purpureum*.

|  |  | Number | Total Length | Percentage (%)^e^ |
| --- | --- | --- | --- | --- |
| All Repeats^a^ | *D. discoideum* | 126,206 | 7,733,867 bp | 22.53 |
|  | *D. purpureum* | 73,752 | 4,239,406 bp | 12.75 |
| Retrotransposon^b^ | *D. discoideum* | 343 | 57,155 bp | 0.17 |
|  | *D. purpureum* | 150 | 20,550 bp | 0.06 |
| Non-retrotransposon^c^ | *D. discoideum* | 125,863 | 7,676,712 bp | 22.36 |
|  | *D. purpureum* | 73,602 | 4,218,658 bp | 12.69 |
| Extant Intron^d^ | *D. discoideum* | 15,510 | 2,040,230 bp |  |
|  | *D. purpureum* | 18,412 | 2,987,579 bp |  |

^a^All repetitive sequences were detected by RepeatMasker (version open-4.0.0, default mode, and RepBase update 20140131, RM database version 20140131).

^b^Only SINEs, LINEs and LTR elements were counted.

^c^Non-retrotransposon includes DNA elements, satellites, simple repeats, and low-complexity repeats.

^d^All annotated introns.

^e^Percentage = total length of repeats/genome length.

Table S4. Exonic sequences flanking lost introns have higher GC contents than those flanking conserved introns.

| Species | Intron type | Number^a^ | Median (%) | Mann-Whitney *U* test |
| --- | --- | --- | --- | --- |
| *Arabidopsis thaliana* | Lost | 101 | 44.5 | 0.023 |
|  | Conserved | 72,966 | 43.5 |  |
| *Brassica rapa* | Lost | 148 | 45.5 | 3 × 10^-5^ |
|  | Conserved | 7,034 | 44.5 |  |
| *Drosophila willistoni* | Lost | 85 | 46.5 | 0.043 |
|  | Conserved | 22,663 | 46.0 |  |
| *Caenorhabditis briggsae* | Lost | 1,048 | 44.5 | 2 × 10^-7^ |
|  | Conserved | 48,468 | 44.0 |  |
| *Caenorhabditis remanei* | Lost | 620 | 44.0 | 0.011 |
|  | Conserved | 48,468 | 43.0 |  |
| *Rattus norvegicus* | Lost | 49 | 53.5 | 0.193 |
|  | Conserved | 123,036 | 51.5 |  |

^a^The numbers of intron losses and conserved introns are slightly smaller than those in the previous publications that we cited. This difference is the result of updates to the genome sequences and annotations and the requirement of 100 bp coding sequences both upstream and downstream of the positions used in the calculation of GC content.

Table S5. At discordant intron positions, the relative GC content of exonic sequences flanking lost introns compared with the exonic sequences flanking extant introns of the sister species^a^.

| Species | Number | Ratio^b^ | Ratio^c^ | Wilcoxon signed rank test |
| --- | --- | --- | --- | --- |
| *Arabidopsis thaliana* | 92 | 1.005650439 | 1 | 0.185 |
| *Brassica rapa* | 120 | 1.021505376 | 1 | 0.001 |
| *Drosophila willistoni* | 71 | 1.010869565 | 0.989473684 | 0.010 |
| *Caenorhabditis briggsae* | 864 | 0.994832007 | 1 | 0.041 |
| *Caenorhabditis remanei* | 531 | 1 | 1 | 0.041 |
| *Rattus norvegicus* | 43 | 1.049180328 | 1.009259259 | 8 × 10^-5^ |

^a^For each intron-lost gene, we determined whether the intron loss position in a sister specie also has a higher GC content. The sister species of the above species are *Arabidopsis lyrata*, *Thellungiella parvula*, *Drosophila melanogaster*, *Caenorhabditis remanei*, *Caenorhabditis briggsae*, and *Mus musculus*, respectively.

^b^Median values of relative GC content for lost introns.

^c^Median values of relative GC content for extant introns.

Table S6. The GC content of exonic sequences flanking extant introns at discordant intron positions compared with those flanking conserved intron sites of the same species.

| Related species | Intron type | Number | Median (%) | Mann-Whitney *U* test |
| --- | --- | --- | --- | --- |
| *Arabidopsis lyrata* | Discordant | 101 | 44.0 | 0.014 |
|  | Conserved | 72,966 | 43.5 |  |
| *Thellungiella parvula* | Discordant | 148 | 44.5 | 0.358 |
|  | Conserved | 7,034 | 44.0 |  |
| *Drosophila melanogaster* | Discordant | 85 | 53.0 | 0.341 |
|  | Conserved | 22,663 | 53.5 |  |
| *Caenorhabditis remanei* | Discordant | 1,048 | 43.5 | 0.0046 |
|  | Conserved | 48,468 | 43.0 |  |
| *Caenorhabditis briggsae* | Discordant | 620 | 44.5 | 4 × 10^-8^ |
|  | Conserved | 48,468 | 44.0 |  |
| *Mus musculus* | Discordant | 49 | 51.0 | 0.576 |
|  | Conserved | 123,036 | 51.5 |  |

Table S7. Version numbers and source databases of the plant and animal genomes used in this study.

| Species | Database | version |
| --- | --- | --- |
| *Arabidopsis thaliana* | Phytozome | V9 |
| *Arabidopsis lyrata* | Phytozome | V9 |
| *Brassica rapa* | Phytozome | V9 |
| *Thellungiella parvula* | Phytozome | V9 |
| *Drosophila willistoni* | FlyBase | R1.3 |
| *Drosophila melanogaster* | FlyBase | R6.04 |
| *Caenorhabditis briggsae* | Ensembl Metazoa | Release 27 |
| *Caenorhabditis remanei* | Ensembl Metazoa | Release 27 |
| *Caenorhabditis elegans* | Ensembl Metazoa | Release 27 |
| *Caenorhabditis japonica* | Ensembl Metazoa | Release 27 |
| *Rattus norvegicus* | Ensembl | Release 79 |
| *Mus musculus* | Ensembl | Release 79 |
